# One-pot, three-component, iron-catalyzed synthesis of benzimidazoles *via* domino C–N bond formation†

**DOI:** 10.1039/d3ra04450e

**Published:** 2023-08-21

**Authors:** Jasem Aboonajmi, Masoumeh Mohammadi, Farhad Panahi, Mahdi Aberi, Hashem Sharghi

**Affiliations:** a Department of Chemistry, College of Sciences, Shiraz University Shiraz 71454 Iran Panahi@shirazu.ac.ir +98 7132280926 +98 7136137136; b Institut für Organische Chemie, Albert-Ludwigs-Universität Freiburg Albertstraße 21 79104 Freiburg im Breisgau Germany; c Department of Chemical and Materials Engineering, Faculty of Shahid Rajaee, Technical and Vocational University (TVU) Shiraz Branch Shiraz Iran

## Abstract

An efficient one-pot, three-component process for the synthesis of benzimidazole derivatives using a catalytic amount of Fe(iii) porphyrin has been developed. The reaction proceeds *via* domino C–N bond formation and cyclization reactions of benzo-1,2-quinone, aldehydes and ammonium acetate as a nitrogen source to selectively produce benzimidazole. A number of benzimidazole derivatives have been synthesized using this method in high yields under mild reaction conditions.

## Abstract 2

A one-pot multicomponent procedure for the efficient synthesis of benzimidazole derivatives *via* coupling of benzo-1,2-quinone, aldehydes, and ammonium acetate using an Fe(iii)-porphyrin catalyst system is presented.

## Graphical abstract

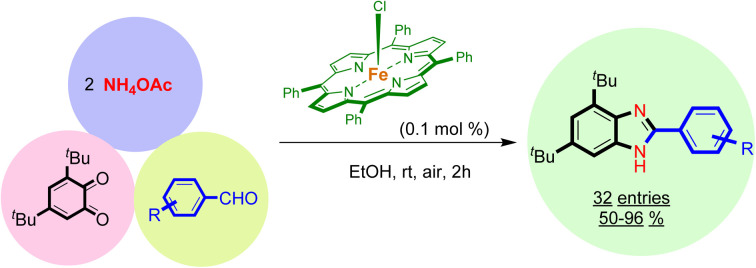

## Introduction

**Scheme 1:**
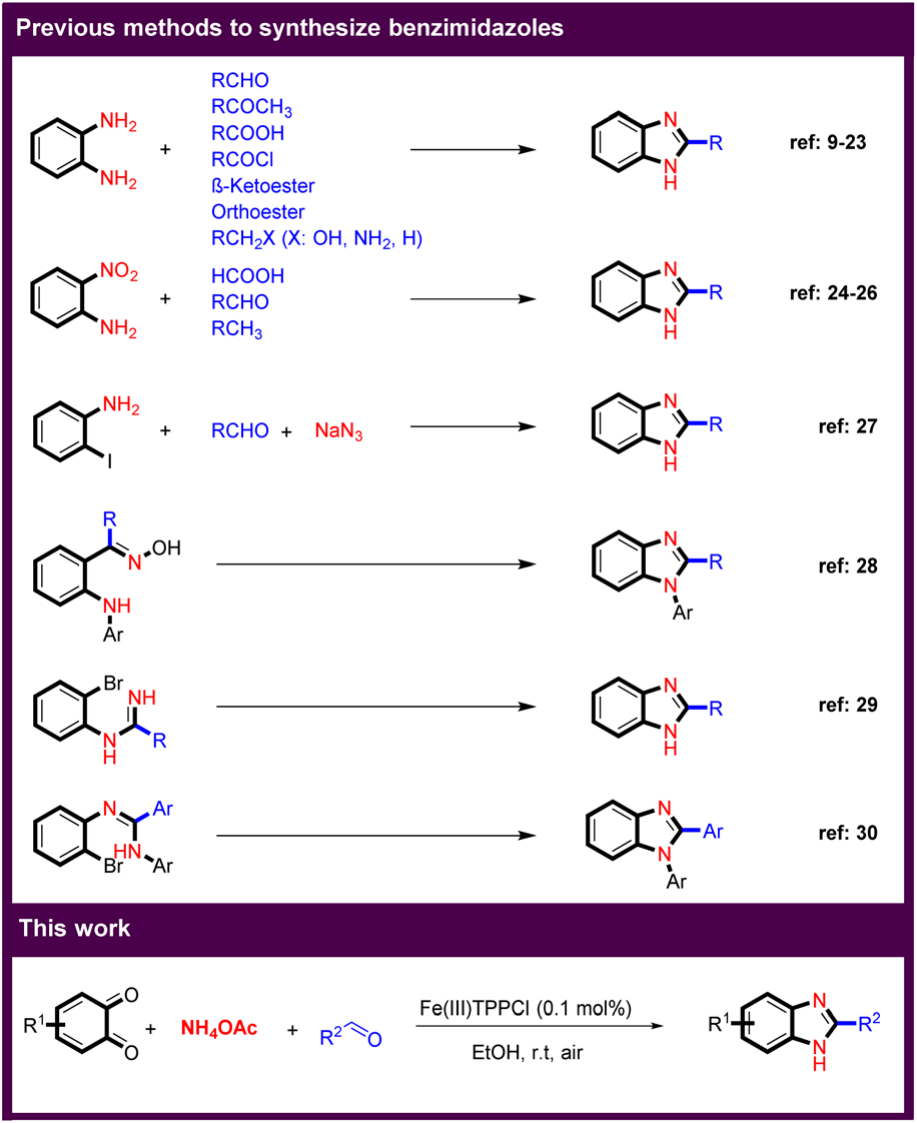
Different methods for the synthesis of benzimidazole scaffolds.

Multicomponent reactions (MCRs) play an essential role in the pharmaceutical industry and in modern organic synthesis, as they provide a powerful tool for the cost and time-efficient synthesis of advanced drugs and target compounds by generating a complex structure from multiple reactants in a single step.^1–5^ Imidazole derivatives are one of the most important groups in heterocyclic compounds that have attracted much attention because they are found in a variety of natural products. In addition, benzimidazoles are the core structure of many pharmaceuticals, ionic liquids (ILs) as more environmentally friendly solvents, and *N*-heterocyclic carbenes as valuable ligands in transition-metal catalysis.^3,6–8^ There are several known routes to benzimidazole derivatives (Scheme 1). Reaction between *o*-phenylenediamine and carbonyl compounds such as aldehydes,^9–13^ ketones,^14^ acids,^15^ acyl chlorides,^16^ and as well as β-ketoesters,^17,18^ or orthoesters,^19,20^ or and benzylic including benzyl alcohols,^21^ benzyl amines,^22^ and toluene derivatives^23^ are the main methods for the synthesis of a large number of benzimidazole derivatives. Another important approach is the reaction of 2-aminonitrobenzenes with acids,^24^ aldehydes,^25^ and activated methyl groups.^26^ In another strategy, benzimidazoles were obtained by coupling of 2-iodoaniline with aldehydes.^27^ Arylamino oximes also led to benzimidazoles in the presence of a base.^28^ The synthesis of benzimidazoles by intramolecular *N*-arylation using copper catalysts was developed.^29^ The intramolecular cyclization of *o*-bromoaryl derivatives also resulted in benzimidazoles.^30^

Despite numerous advances in the synthesis of benzimidazoles, the development of new synthetic methods is strongly considered because the existing methods have many drawbacks, such as low yields, complicated reaction conditions, use of toxic reagents and solvents. On the other hand, there is a high demand for efficient and clean synthesis of benzimidazole derivatives from other sources. Continuing our program on the synthesis of benzoxazoles,^4,5,31,32^ we would like to present here a novel and efficient protocol for the synthesis of benzimidazole derivatives. In this work, MCR of benzo-1,2-quinones, aryl aldehydes and ammonium acetate in the presence of catalytic amount of an Fe(iii)-porphyrin catalyst at room temperature resulted in a benzimidazole core.

Metalloporphyrins have been used as catalyst in many organic reactions such as the hydroxylation and epoxidation of hydrocarbon compounds,^33,34^ the hydroxylation of aromatic compounds,^35^ the aziridination of olefins,^36^ oxidation of sulfides to sulfones,^37^ the ring-opening of epoxides,^38^ and the synthesis of heterocyclic compounds.^10,39,40^

## Results and discussion

To optimize the new method for the synthesis of benzimidazoles, the reaction of 3,5-di-*tert*-butylcyclohexa-3,5-diene-1,2-dione (1), ammonium acetate (2), and 4-methoxybenzaldehyde (3a) was selected as a model reaction (Table 1). In the absence of catalyst, in ethanol as solvent and at 80 °C, only 5% product was observed (Table 1, entry 1). In an attempt to improve the reaction yield, some transition metals such as Fe, Zn, Ni, Cu, Cd and Mn were tested as catalysts (Table 1, entries 2–7). Among the catalysts tested, an increase of the reaction yield to 65% was observed using FeCl_3_. However, a benzoxazole by-product was also isolated using these catalysts (Table 1, entries 2–7). To enhance the yield and selectivity, different iron sources were tested (Table 1, entries 8–10). Surprisingly, the maximum product was obtained with a Fe^III^-porphyrin complex (Fe^III^TPPCl) at room temperature after only 2 h without the formation of the benzoxazole 5a side-product (Table 1, entry 11).

**Table 1:** Optimization of the reaction conditions for the synthesis of benzimidazoles *via* a one-pot multicomponent reaction^a^

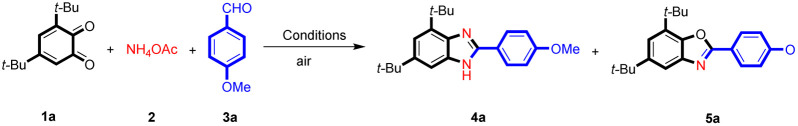					
#	Cat. (mol%)	Solv.	*T* (°C)	Time (h)	Yield 4a/5a^b^ (%)
1	—	EtOH	80	12	5/—
2	FeCl_3_ (5)	EtOH	rt	12	65/15
3	ZnCl_2_ (5)	EtOH	rt	12	28/5
4	NiCl_2_ (5)	EtOH	rt	12	36/10
5	CuCl_2_ (5)	EtOH	rt	12	45/8
6	CdCl_2_ (5)	EtOH	rt	12	25/5
7	MnCl_2_ (5)	EtOH	rt	12	50/20
8	Fe(NO_3_)_3_ (5)	EtOH	rt	12	45/20
9	FeBr_3_ (5)	EtOH	rt	12	22/18
10	Fe(acac)_3_ (5)	EtOH	rt	12	20/10
**11**	**Fe** ^**III**^ **TPPCl (0.1)**	**EtOH**	**rt**	**2**	**96/0**
12	Sn^II^TPP (0.1)	EtOH	rt	7	65/0
13	Pb^II^TPP (0.1)	EtOH	rt	8	60/0
14	Zn^II^TPP (0.1)	EtOH	rt	5	70/0
15	Cd^II^TPP (0.1)	EtOH	rt	9	60/0
16	Ni^II^TPP (0.1)	EtOH	rt	3	77/0
17	Cu^II^TPP (0.1)	EtOH	rt	4	80/0
18	Fe^III^TPPCl (0.05)	EtOH	rt	12	78/0
19	Fe^III^TPPCl (0.15)	EtOH	rt	2	94/0
20	Fe^III^TPPCl (0.1)	MeCN	rt	3	70/10
21	Fe^III^TPPCl (0.1)	H_2_O	rt	9	45/5
22	Fe^III^TPPCl (0.1)	MeOH	rt	3	86/5
23	Fe^III^TPPCl (0.1)	PhMe	rt	7	67/0
24	Fe^III^TPPCl (0.1)	EtOH	rt	5	c55/0

a Reaction conditions: 1a (1.0 mmol), 2a (2.2 mmol), 3a (1.0 mmol), and solvent (5.0 mL) at r. t.

b Isolated yield.

c Under nitrogen atmosphere.

Since the porphyrin complex was found to be the best catalyst for this reaction, the porphyrin complexes of other metals were also synthesized^10,40^ and tested to verify that iron was the best catalyst for this reaction (Table 1, entries 12–17). In the presence of Sn^II^TPP, about 65% of the product was isolated after 7 hours at room temperature (Table 1, entry 12). With Pb^II^TPP, 4a was prepared in 60% yield after 8 hours (Table 1, entry 13). Good yield was obtained with Zn^II^TPP (Table 1, entry 14). Cd^II^TPP worked the same way as Pb^II^TPP and 60% of the product was isolated (Table 1, entry 15). Interestingly, Ni^II^TPP and Cu^II^TPP gave comprisable yields of product (Table 1, entries 16 and 17). The interesting thing is that with the use of porphyrin complexes as catalysts, we have high selectivity for the formation of the benzimidazole product. Among the tested metal complexes, Fe^III^TPPCl showed high catalytic activity and was therefore selected as catalyst for this multicomponent synthesis of benzimidazoles.

For further optimization, different catalyst loadings were used for the reaction, and no further improvements were found (Table 1, entries 18 and 19). Different solvents were also investigated and no superiority was found (Table 1, entries 20–23). For example, in the solvents acetonitrile and methanol, 70% and 86% of the product were isolated respectively, albeit with a slight loss of selectivity. In addition, the reaction was carried out under N_2_ and a yield of 55% was obtained (Table 1, entry 24).

**Scheme 2:**
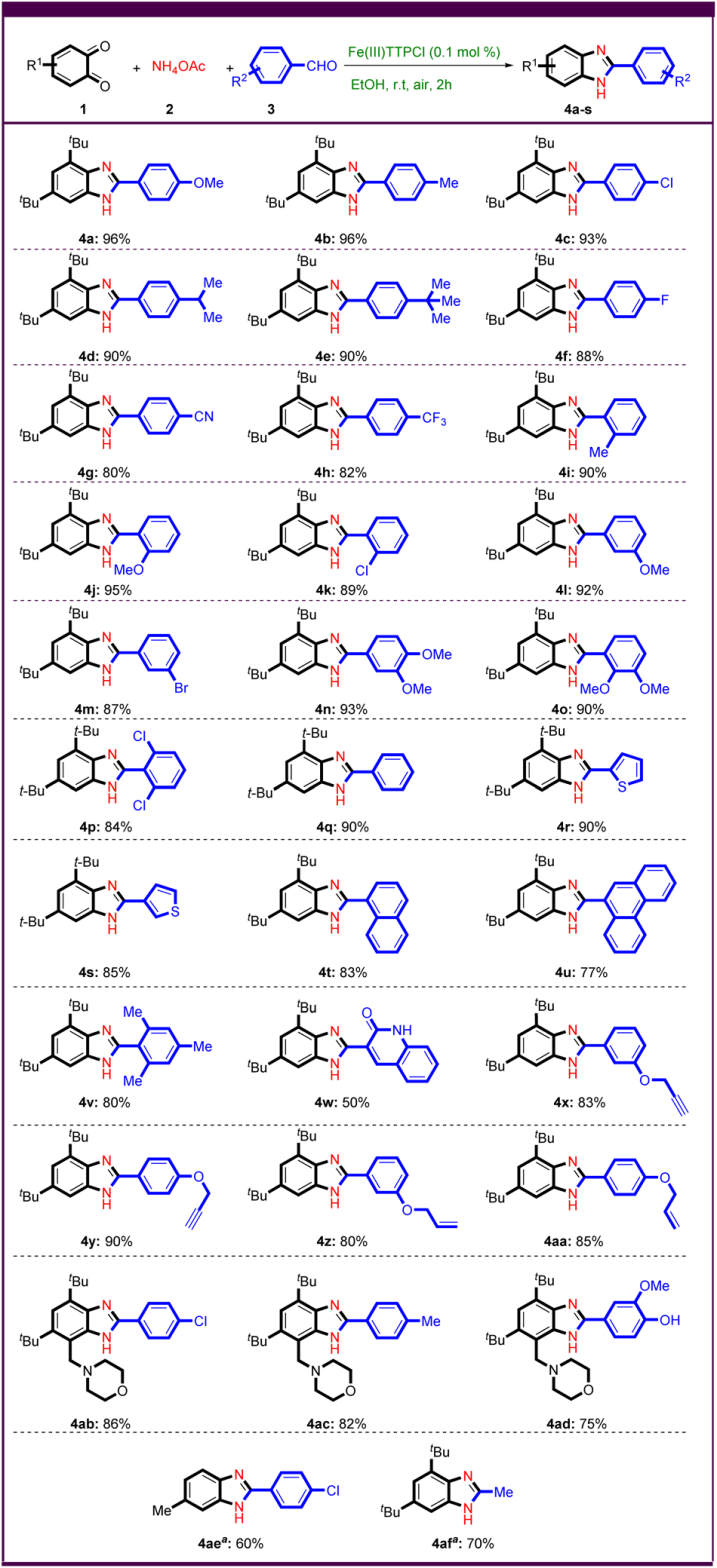
Synthesis of benzimidazolederivatives. Reaction conditions: 1 (1.0 mmol), 2 (2.2 mmol) and 3 (1.0 mmol) in the presence of FeTTPCl (0.1 mol%) in EtOH (5.0 mL) at room temperature under air condition for 2 h. All yields are isolated. ^a^ The reaction was performed at 60 °C, 6 h.

After optimizing the reaction conditions, various aryl aldehydes with electron donor and electron withdrawing groups were first investigated under optimized conditions to test the generality and scope of the method (Scheme 2).

Aryl aldehydes with electron-donating groups in *para* position such as –OMe, –Me, –Cl, *–*iPr, and -^*t*^Bu gave benzimidazoles 4a–e in more than 90% yield. Electron withdrawing groups such as –F, –CN, and –CF_3_ were tested in this reaction and gave good yields of benzimidazoles 4f–h. The *ortho*- or *meta*-substituted benzaldehyde worked well and provided the desired products in good to excellent yields, regardless of their electronic nature (Scheme 2, 4i–m).

To further investigate the reaction possibilities, aryl aldehydes with two substituents such as 3,4-dimethoxybenzaldehyde, 2,3-dimethoxybenzaldehyde and 2,6-dichlorobenzaldehyde were tested (Scheme 2, 4n–p). Remarkably, various heteroaryl aldehydes such as 2-thiophene carboxaldehyde and 3-thiophene carboxaldehyde gave good yields of the desired products (Scheme 2, 4r,s). Encouragingly, 1-naphthaldehyde, and phenanthrene-9-carbaldehyde were subjected to Fe(iii)-catalyzed domino C–N bond formation as polyaromatic substrates, resulting in the corresponding benzimidazoles in 83%, and 77% yields, respectively (Scheme 2, 4t,u). Sterically hindered substrates were found to lead to the corresponding benzimidazoles in good yields (Scheme 2, 4p,v).

The use of quinoline-3-carbaldehyde as substrate gave a bis-heterocyclic product 4w in 50% yield.

Alkene and alkyne functional groups tolerated the reaction conditions well, giving allyl- and propargyl-functionalized benzimidazoles in high yields (Scheme 2, 4x–aa). To expand the scope of this method, we attempted to synthesize poly-substituted benzo-1,2-quinone derivative with heterocycle.^41^ The synthetic benzo-1,2-quinone derivative derived from morpholine worked quite well and yielded benzimidazoles in good yields (Scheme 2, 4ab–ad). The desired product 4ae was also successfully prepared from other benzo-1,2-quinones. Remarkably, acetaldehyde as an aliphatic substrate led to the synthesis of benzimidazole 4af in 70% yield.

**Scheme 3:**
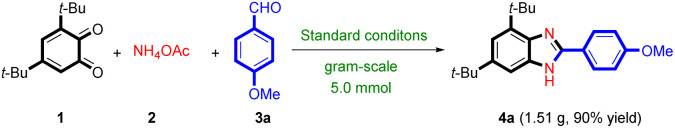
Gram-scale Synthesis.

To demonstrate the efficiency of the synthesis, we performed a large-scale reaction producing compound 4a in 90% yield (Scheme 3).

**Scheme 4:**
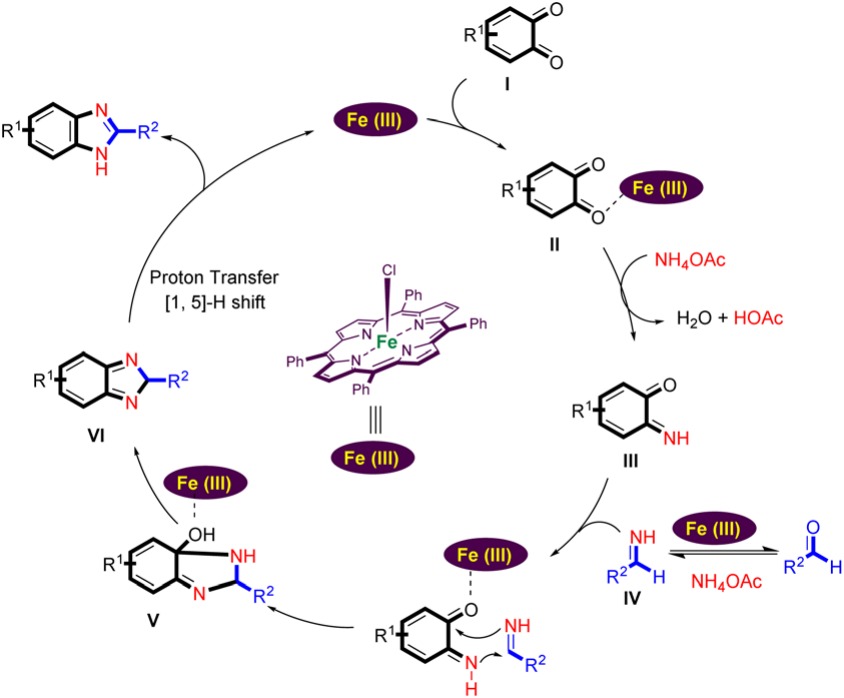
The proposed mechanism.

A plausible reaction mechanism for the Fe(iii)-porphyrin-catalyzed formation of the benzimidazoles from benzo-1,2-quinone, ammonium acetate, and aldehydes is shown in Scheme 4.

Coordination of the carbonyl group of benzo-1,2-quinone (I) with the Lewis acid site of Fe(iii) porphyrin leads to its activation. It now reacts with ammonium acetate to form the imine intermediate (III).^42,43^ At the same time, aldehyde can react with an excess of ammonium acetate to form a Schiff base IV.^44–48^ Schiff base IV is able to react with intermediate (III) by intermolecular cyclization to form intermediate V. When the intermediate V is formed, a dehydration process could take place to obtain the intermediate (VI). Finally, the desired benzimidazole is generated by [1,5]-*H* shift (VI) and the Fe(iii) porphyrin catalyst is regenerated to start the next cycle.^2,49^

## Conclusions

In summary, we have demonstrated an efficient, novel, green and simple procedure for the multicomponent one-pot synthesis of benzimidazoles in the presence of Fe(iii) porphyrin complexes of benzo-1,2-quinone, NH_4_OAc as nitrogen source and aldehydes. Key features of this process include mild reaction conditions, large-scale synthesis and the use of environmentally friendly organic solvents in the reaction process, providing an efficient method for the preparation of benzimidazoles.

## Conflicts of interest

There are no conflicts to declare.

## Supplementary Material

RA-013-D3RA04450E-s001
