# Supplementary material for: One-pot, three-component, iron-catalyzed synthesis of benzimidazoles via domino C–N bond formation
Source: RSC Adv. 2023 Aug 21;13(35):24789–94. doi: 10.1039/d3ra04450e (PMC10440634; doi:10.1039/d3ra04450e)
Supplement: RA-013-D3RA04450E-s001 [file RA-013-D3RA04450E-s001.pdf]

Electronic Supplementary Material (ESI) for RSC Advances.
This journal is © The Royal Society of Chemistry 2023

Supporting Information

One-pot, Three-component, Iron-Catalyzed Synthesis of Benzimidazoles via Domino C–N Bond Formation

Jasem Aboonajmi,[a] Masoumeh Mohammadi,[a] Farhad Panahi,*[a,b] Mahdi Aberi,[c] and Hashem Sharghi[a]

[a] Department of Chemistry, College of Sciences, Shiraz University, Shiraz 71454, Iran

[b]Institut für Organische Chemie, Albert-Ludwigs-Universität Freiburg, Albertstraße 21, 79104 Freiburg im Breisgau, Germany

[c] Department of Chemical and Materials Engineering, Faculty of ShahidRajaee, Shiraz Branch, Technical and Vocational University (TVU), Shiraz, Iran

Corresponding author: Email: Panahi@shirazu.ac.ir

Table of Contents

Contents Page No.

Experimental Section. **S4**

General procedure for the synthesis of benzimidazoles. **S4**

Product characterization data. **S5-8**

Figure S1. ^{1}H NMR spectrum of compound 4a in DMSO-d_6 (250 MHz). **S9**

Figure S2. ^{13}C NMR spectrum of compound 4a in DMSO-d_6 (100 MHz). **S9**

Figure S3. ^{1}H NMR spectrum of compound 4b in DMSO-d_6 (400 MHz). **S10**

Figure S4. ^{13}C NMR spectrum of compound 4b in DMSO-d_6 (100 MHz). **S10**

Figure S5. ^{1}H NMR spectrum of compound 4c in DMSO-d_6 (250 MHz). **S11**

Figure S6. ^{13}C NMR spectrum of compound 4c in DMSO-d_6 (100 MHz). **S11**

Figure S7. ^{1}H NMR spectrum of compound 4d in DMSO-d_6 (250 MHz). **S12**

Figure S8. ^{13}C NMR spectrum of compound 4d in DMSO-d_6 (100 MHz). **S12**

Figure S9. ^{1}H NMR spectrum of compound 4e in DMSO-d_6 (400 MHz). **S13**

Figure 10. ^{13}C NMR spectrum of compound 4e in DMSO-d_6 (100 MHz). **S13**

Figure S11. ^{1}H NMR spectrum of compound 4f in DMSO-d_6 (250 MHz). **S10**

Figure S12. ^{13}C NMR spectrum of compound 4f in DMSO-d_6 (100 MHz). **S14**

Figure S13. ^{1}H NMR spectrum of compound 4g in DMSO-d_6 (400 MHz). **S14**

Figure S14. ^{13}C NMR spectrum of compound 4g in DMSO-d_6 (100 MHz). **S15**

Figure S15. ^{1}H NMR spectrum of compound 4h in DMSO-d_6 (400 MHz). **S16**

Figure S16. ^{13}C NMR spectrum of compound 4h in DMSO-d_6 (100 MHz). **S16**

Figure S17. ^{1}H NMR spectrum of compound 4i in DMSO-d_6 (250 MHz). **S17**

Figure S18. ^{13}C NMR spectrum of compound 4i in DMSO-d_6 (100 MHz). **S17**

Figure S19. ^{1}H NMR spectrum of compound 4j in DMSO-d_6 (400 MHz). **S18**

Figure S20. ^{13}C NMR spectrum of compound 4j in DMSO-d_6 (100 MHz). **S18**

Figure S21. ^{1}H NMR spectrum of compound 4k in DMSO-d_6 (250 MHz). **S19**

Figure S22. ^{13}C NMR spectrum of compound 4k in DMSO-d_6 (100 MHz). **S19**

Figure S23. ^{1}H NMR spectrum of compound 4l in DMSO-d_6 (250 MHz). **S20**

Figure S24. ^{13}C NMR spectrum of compound 4l in DMSO-d_6 (100 MHz). **S20**

Figure S25. ^{1}H NMR spectrum of compound 4m in DMSO-d_6 (250 MHz). **S21**

Figure S26. ^{13}C NMR spectrum of compound 4m in DMSO-d_6 (100 MHz). **S21**

Figure S27. ^{1}H NMR spectrum of compound 4n in DMSO-d_6 (250 MHz). **S22**

Figure S28. ^{13}C NMR spectrum of compound 4n in DMSO-d_6 (100 MHz). **S22**

Figure S29. ^{1}H NMR spectrum of compound 4o in DMSO-d_6 (250 MHz). **S23**

Figure S30. ^{13}C NMR spectrum of compound 4o in DMSO-d_6 (100 MHz). **S23**

Figure S31. ^{1}H NMR spectrum of compound 4p in DMSO-d_6 (250 MHz). **S24**

Figure S32. ^{13}**C NMR spectrum of compound 4p in DMSO-*d*$_6$ (100 MHz).** **S24**
Figure S33. ^{1}**H NMR spectrum of compound 4q in DMSO-*d*$_6$ (250 MHz).** **S25**
Figure S34. ^{13}**C NMR spectrum of compound 4q in DMSO-*d*$_6$ (100 MHz).** **S25**
Figure S35. ^{1}**H NMR spectrum of compound 4r in DMSO-*d*$_6$ (250 MHz).** **S26**
Figure S36. ^{13}**C NMR spectrum of compound 4r in DMSO-*d*$_6$ (100 MHz).** **S26**
Figure S37. ^{1}**H NMR spectrum of compound 4s in DMSO-*d*$_6$ (250 MHz).** **S27**
Figure S38. ^{13}**C NMR spectrum of compound 4s in DMSO-*d*$_6$ (100 MHz).** **S27**
Figure S39. ^{1}**H NMR spectrum of compound 4t in DMSO-*d*$_6$ (250 MHz).** **S28**
Figure S40. ^{13}**C NMR spectrum of compound 4t in DMSO-*d*$_6$ (100 MHz).** **S28**
Figure S41. ^{1}**H NMR spectrum of compound 4u in DMSO-*d*$_6$ (400 MHz).** **S29**
Figure S42. ^{13}**C NMR spectrum of compound 4u in DMSO-*d*$_6$ (100 MHz).** **S29**
Figure S43. ^{1}**H NMR spectrum of compound 4vin DMSO-*d*$_6$ (400 MHz).** **S30**
Figure S44. ^{13}**C NMR spectrum of compound 4v in DMSO-*d*$_6$ (100 MHz).** **S30**
Figure S45. ^{1}**H NMR spectrum of compound 4w in DMSO-*d*$_6$ (400 MHz).** **S31**
Figure S46. ^{13}**C NMR spectrum of compound 4w in** $CDCl_3$**(100 MHz).** **S31**
Figure S47. ^{1}**H NMR spectrum of compound 4x in DMSO-*d*$_6$ (400 MHz).** **S32**
Figure S48. ^{13}**C NMR spectrum of compound 4x in DMSO-*d*$_6$ (100 MHz).** **S32**
Figure S49. ^{1}**H NMR spectrum of compound 4y in DMSO-*d*$_6$ (400 MHz).** **S33**
Figure S50. ^{1}**H NMR spectrum of compound 4z in DMSO-*d*$_6$ (400 MHz).** **S34**
Figure S51. ^{13}**C NMR spectrum of compound 4z in DMSO-*d*$_6$ (100 MHz).** **S34**
Figure S52. ^{1}**H NMR spectrum of compound 4aa in DMSO-*d*$_6$ (400 MHz).** **S35**
Figure S53. ^{13}**C NMR spectrum of compound 4aa in DMSO-*d*$_6$ (100 MHz).** **S35**
Figure S54. ^{1}**H NMR spectrum of compound 4ab in DMSO-*d*$_6$ (400 MHz).** **S36**
Figure S55. ^{13}**C NMR spectrum of compound 4ab in DMSO-*d*$_6$ (100 MHz).** **S36**
Figure S56. ^{1}**H NMR spectrum of compound 4ac in DMSO-*d*$_6$ (400 MHz).** **S37**
Figure S57. ^{13}**C NMR spectrum of compound 4ac in** $CDCl_3$ **(100 MHz).** **S37**
Figure S58. ^{1}**H NMR spectrum of compound 4ad in DMSO-*d*$_6$ (400 MHz).** **S38**
Figure S59. ^{13}**C NMR spectrum of compound 4ad in DMSO-*d*$_6$ (100 MHz).** **S38**
Figure S60. ^{1}**H NMR spectrum of compound 4ae in DMSO-*d*$_6$ (400 MHz).** **S39**
Figure S61. ^{13}**C NMR spectrum of compound 4ae in DMSO-*d*$_6$ (100 MHz).** **S39**
Figure S62. ^{1}**H NMR spectrum of compound 4af in DMSO-*d*$_6$ (400 MHz).** **S40**
Figure S63. ^{13}**C NMR spectrum of compound 4af in DMSO-*d*$_6$ (100 MHz).** **S40**

Experimental Section

General Information. The spectral data of known products were comprised with literature. Reagents and solvents were purchased from chemical companies. Melting points were determined in capillary tubes. The progress of the reaction and the purity of compounds were monitored by thin layer chromatography (TLC) analytical silica gel plates (Merck 60 F250). The ^{1}H-NMR (250 and 400 MHz) and ^{13}C-NMR (100 MHz) were run on 250 and 400 NMR spectrometers. The elemental analysis was performed on a microanalyzer.

General procedure for the synthesis of benzimidazoles

In a 25 mL round-bottom flask contained a suspension of Fe(III)-porphyrin complex (0.1 mol %) in ethanol (5.0 mL), benzo-1,2-quinone (1.0 mmol), NH_4OAc (2.2 mmol), and aromatic aldehyde (1.0 mmol) were added and the resulting mixture was stirred at room temperature under the air atmosphere. The progress of the reaction was monitored by thin-layer chromatography (TLC). After completion of the reaction, water was added to the solution and products was extracted with EtOAc dried over anhydrous Na_2SO_4 and concentrated under reduced pressure. The crude products were purified by column chromatography on silica gel to give the pure products.

Product characterization data:

4,6-di-*tert*-butyl-2-(4-methoxyphenyl)-1*H*-benzo[*d*]imidazole (4a). Purified by column chromatography on silica gel and eluted with ethyl acetate/petroleum ether (5:100). Isolated yield: 323 mg, 96%. Colorless liquid; ^{1}H NMR (250 MHz, DMSO-d_6): δ (ppm) 1.33 (s, 9H), 1.55 (s, 9H), 3.81 (s, 3H), 7.05 (d, *J*= 2.5 Hz, 1H), 7.09 (d, *J*= 7.5 Hz, 2H), 7.25 (d, *J*= 2.5 Hz, 1H), 8.06 (d, *J*= 10.0 Hz, 2H), 12.48 (s, NH); ^{13}C NMR (100 MHz, DMSO-d_6): δ (ppm) 35.4, 36.8, 39.8, 40.3, 60.5, 110.3, 119.5, 120.1, 132.9, 140.4, 140.5, 145.0, 149.6, 154.1, 154.2, 165.4. Anal. Calcd for $C_{22}H_{28}N_2O$: C, 78.53; H, 8.39; N, 8.33; Found: C, 78.41; H, 8.30; N, 8.29.

4,6-di-*tert*-butyl-2-(*p*-tolyl)-1*H*-benzo[*d*]imidazole (4b). Purified by column chromatography on silica gel and eluted with ethyl acetate/petroleum ether (5:100). Isolated yield: 307 mg, 96%. Cream solid, mp: 224-225 ^{o}C; ^{1}H NMR (400 MHz, DMSO-d_6): δ (ppm) 1.36 (s, 9H), 1.59 (s, 9H), 2.38 (s, 3H), 7.09 (s, 1H), 7.30 (s, 1H), 7.34 (d, *J*= 8.0 Hz, 2H), 8.05 (d, *J*= 8.0 Hz, 2H), 12.67 (s, NH); ^{13}C NMR (100 MHz, DMSO-d_6): δ (ppm) 20.9, 30.2, 31.6, 34.6, 35.1, 105.1, 114.9, 126.1, 127.8, 129.3, 135.3, 138.8, 139.9, 140.0, 144.4, 149.0. Anal. Calcd for $C_{22}H_{28}N_2$: C, 82.45; H, 8.81; N, 8.74; Found: C, 82.14; H, 8.68; N, 8.65.

4,6-di-*tert*-butyl-2-(4-chlorophenyl)-1*H*-benzo[*d*]imidazole (4c). Purified by column chromatography on silica gel and eluted with ethyl acetate/petroleum ether (5:100). Isolated yield: 317 mg, 93%. Colorless liquid; ^{1}H NMR (250 MHz, DMSO-d_6): δ (ppm) 1.33 (s, 9H), 1.55 (s, 9H), 7.09 (d, *J*= 2.5 Hz, 1H), 7.29 (d, *J*= 2.5 Hz, 1H), 7.59 (d, *J*= 10.0 Hz, 2H), 8.16 (d, *J*= 7.5 Hz, 2H), 12.88 (s, NH); ^{13}C NMR (100 MHz, DMSO-d_6): δ (ppm) 30.2, 31.6, 34.6, 35.2, 105.2, 115.2, 127.8, 128.9, 129.4, 133.9, 135.4, 139.8, 140.2, 145.0, 147.7. Anal. Calcd for $C_{21}H_{25}ClN_2$: C, 73.99; H, 7.39; N, 8.22; Found: C, 73.81; H, 7.30; N, 8.31.

4,6-di-*tert*-butyl-2-(4-isopropylphenyl)-1*H*-benzo[*d*]imidazole (4d). Purified by column chromatography on silica gel and eluted with ethyl acetate/petroleum ether (5:100). Isolated yield: 313 mg, 90%. Colorless liquid; ^{1}H NMR (250 MHz, DMSO-d_6): δ (ppm) 1.22 (d, *J*= 7.5 Hz, 6H), 1.32 (s, 9H), 1.55 (s, 9H), 2.84-3.00 (m, 1H), 7.06 (d, *J*= 2.5 Hz, 1H), 7.28 (d, *J*= 2.5 Hz, 1H), 7.37 (d, *J*= 10.0 Hz, 2H), 8.06 (d, *J*= 7.5 Hz, 2H), 12.76 (s, NH); ^{13}C NMR (100 MHz, DMSO-d_6): δ (ppm) 23.7, 29.6, 30.2, 31.5, 31.6, 33.3, 34.5, 105.1, 114.8, 126.2, 126.7, 128.2, 135.3, 139.9, 144.4, 149.0, 149.7. Anal. Calcd for $C_{24}H_{32}N_2$: C, 82.71; H, 9.25; N, 8.04; Found: C, 82.66; H, 9.13; N, 7.97.

4,6-di-*tert*-butyl-2-(4-(*tert*-butyl)phenyl)-1*H*-benzo[*d*]imidazole (4e). Purified by column chromatography on silica gel and eluted with ethyl acetate/petroleum ether (5:100). Isolated yield: 326 mg, 90%. Colorless liquid; ^{1}H NMR (400 MHz, DMSO-d_6): δ (ppm) 1.33 (s, 9H), 1.36 (s, 9H), 1.59 (s, 9H), 7.09 (d, *J* = 4.0 Hz, 1H), 7.30 (d, *J* = 4.0 Hz, 1H), 7.56 (d, *J* = 8.0 Hz, 2H), 8.08 (d, *J* = 8.0 Hz, 2H), 12.60 (s, NH); ^{13}C NMR (100 MHz, DMSO-d_6): δ (ppm) 30.2, 30.9, 31.6, 34.5, 34.6, 35.2, 105.1, 114.9, 125.6, 125.9, 127.8, 128.3, 135.3, 139.9, 144.5, 148.9, 151.9. Anal. Calcd for $C_{25}H_{34}N_2$: C, 82.82; H, 9.45; N, 7.73; Found: C, 82.69; H, 9.37; N, 7.80.

4,6-di-*tert*-butyl-2-(4-fluorophenyl)-1*H*-benzo[*d*]imidazole (4f). Purified by column chromatography on silica gel and eluted with ethyl acetate/petroleum ether (5:100). Isolated yield: 285 mg, 88%. Cream solid, mp: 145-146 ^{o}C; ^{1}H NMR (250 MHz, DMSO-d_6): δ (ppm) 1.33 (s, 9H), 1.56 (s, 9H), 7.08 (d, *J*= 2.5 Hz, 1H), 7.28 (d,*J*= 2.5 Hz, 1H), 7.36 (t, *J*= 8.7 Hz, 3H), 8.15-8.21 (m, 2H), 12.72 (s, NH); ^{13}C NMR (100 MHz, DMSO-d_6): δ (ppm) 30.1, 31.5, 34.5, 35.1, 106.2, 115.1, 115.85 (d, J_{C-F}= 22.0 Hz), 127.0 (d, J_{C-F}= 3.0 Hz), 128.3 (d, J_{C-F}= 8.0 Hz), 135.4, 139.9 (d, J_{C-F}= 36.0 Hz), 144.8, 148.0, 161.5, 163.9. Anal. Calcd for $C_{21}H_{25}FN_2$: C, 77.74; H, 7.77; N, 8.63; Found: C, 77.60; H, 7.68; N, 8.57.

4-(4,6-di-*tert*-butyl-1*H*-benzo[*d*]imidazol-2-yl)benzonitrile (4g). Purified by column chromatography on silica gel and eluted with ethyl acetate/petroleum ether (5:100). Isolated yield: 265 mg, 80%. Colorless liquid; ^{1}H NMR (400 MHz, DMSO-d_6): δ (ppm) 1.42 (s, 9H), 1.64 (s, 9H), 7.20 (d, *J* = 4.0 Hz, 1H), 7.39 (d, *J* = 4.0 Hz, 1H), 8.09 (d, *J* = 8.0 Hz, 2H), 8.38 (d, *J* = 8.0 Hz, 2H), 13.11 (s, NH); ^{13}C NMR (100 MHz, DMSO-d_6): δ (ppm) 30.2, 31.5, 34.7, 35.2, 105.4, 111.2, 115.6, 118.7, 126.6, 132.9, 134.6, 135.6, 140.0, 140.6, 145.7, 147.0. Anal. Calcd for $C_{22}H_{25}N_3$: C, 79.72; H, 7.60; N, 12.68; Found: C, 79.51; H, 7.38; N, 12.76.

4,6-di-*tert*-butyl-2-(4-(trifluoromethyl)phenyl)-1*H*-benzo[*d*]imidazole (4h). Purified by column chromatography on silica gel and eluted with ethyl acetate/petroleum ether (5:100). Isolated yield: 307 mg, 82%. Cream solid, mp: 182-183 ^{o}C; ^{1}H NMR (400 MHz, DMSO-d_6): δ (ppm) 1.36 (s, 9H), 1.59 (s, 9H), 7.13 (d, *J*= 4.0 Hz, 1H), 7.34 (d, *J*= 4.0 Hz, 1H), 7.92 (d, *J*= 8.0 Hz, 2H), 8.37 (d, *J*= 8.0 Hz, 2H), 12.97 (s, NH); ^{13}C NMR (100 MHz, DMSO-d_6): δ (ppm) 30.2, 31.5, 34.6, 35.2, 105.4, 115.4, 122.8, 125.8 (q, J_{C-F}= 4.0 Hz), 126.7, 128.6 (q, J_{C-F}= 32.0 Hz), 134.3, 135.5, 139.9, 140.5, 145.4, 147.2. Anal. Calcd for $C_{22}H_{25}F_3N_2$: C, 70.57; H, 6.73; N, 7.48; Found: C, 70.45; H, 6.61; N, 7.39.

4,6-di-*tert*-butyl-2-(*o*-tolyl)-1*H*-benzo[*d*]imidazole (4i). Purified by column chromatography on silica gel and eluted with ethyl acetate/petroleum ether (5:100). Isolated yield: 288 mg, 90%. Colorless liquid; ^{1}H NMR (250 MHz, DMSO-d_6): δ (ppm) 1.33 (s,

9H), 1.54 (s, 9H), 2.64 (s, 3H), 7.08 (d, *J*= 2.5 Hz, 1H), 7.29 (d, *J*= 2.5 Hz, 1H), 7.33-7.36 (m, 3H), 7.71-7.75 (m, 1H), 12.44 (s, NH); ^{13}C NMR (100 MHz, DMSO-d_6): δ (ppm) 26.3, 35.3, 36.9, 39.8, 40.3, 110.5, 120.1, 131.3, 134.0, 134.2, 135.3, 136.6, 139.8, 142.1, 144.9, 145.4, 150.0, 154.7. Anal. Calcd for $C_{22}H_{28}N_2$: C, 82.45; H, 8.81; N, 8.74; Found: C, 82.37; H, 8.69; N, 8.71.

4,6-di-*tert*-butyl-2-(2-methoxyphenyl)-1*H*-benzo[*d*]imidazole (4j). Purified by column chromatography on silica gel and eluted with ethyl acetate/petroleum ether (5:100). Isolated yield: 319 mg, 95%. Colorless liquid; ^{1}H NMR (400 MHz, DMSO-d_6): δ (ppm) 1.36 (s, 9H), 1.59 (s, 9H), 4.02 (s, 3H), 7.09 (d, *J*= 4.0 Hz, 1H), 7.13 (d, *J*= 4.0 Hz, 1H), 7.22 (d, *J*= 8.0 Hz, 1H), 7.43-7.48 (m, 2H), 8.34 (dd, *J*= 4.0, 8.0 Hz, 1H), 11.92 (s, NH); ^{13}C NMR (100 MHz, DMSO-d_6): δ (ppm) 30.3, 31.7, 34.6, 35.2, 55.6, 99.5, 105.9, 112.0, 114.9, 118.596, 120.798, 129.6, 130.7, 135.1, 139.6, 144.1, 146.6, 156.5. Anal. Calcd for $C_{22}H_{28}N_2O$: C, 78.53; H, 8.39; N, 8.33; Found: C, 78.39; H, 8.24; N, 8.22.

4,6-di-*tert*-butyl-2-(2-chlorophenyl)-1*H*-benzo[*d*]imidazole (4k). Purified by column chromatography on silica gel and eluted with ethyl acetate/petroleum ether (5:100). Isolated yield: 303 mg, 89%. Colorless liquid; ^{1}H NMR (250 MHz, DMSO-d_6): δ (ppm) 1.34 (s, 9H), 1.55 (s, 9H), 7.11 (d, *J*= 2.5 Hz, 1H), 7.33 (d, *J*= 2.5 Hz, 1H), 7.49-7.52 (m, 2H), 7.61-7.64 (m, 1H), 7.86-7.90 (m, 1H), 12.46 (s, NH); ^{13}C NMR (100 MHz, DMSO-d_6): δ (ppm) 30.2, 31.64 34.6, 35.18, 105.55 115.1, 127.4, 130.2, 130.4, 130.8, 131.5, 131.9, 135.0, 139.4, 140.4, 145.0, 146.6. Anal. Calcd for $C_{21}H_{25}ClN_2$: C, 73.99; H, 7.39; N, 8.22; Found: C, 74.05.41; H, 7.48; N, 8.35.

4,6-di-*tert*-butyl-2-(3-methoxyphenyl)-1*H*-benzo[d]imidazole (4l). Purified by column chromatography on silica gel and eluted with ethyl acetate/petroleum ether (5:100). Isolated yield: 309 mg, 92%. Colorless liquid; ^{1}H NMR (250 MHz, DMSO-d_6): δ (ppm) 1.34 (s, 9H), 1.57 (s, 9H), 3.84 (s, 3H), 7.01 (d, *J*= 7.5 Hz, 1H), 7.09 (d, *J*= 2.5 Hz, 1H), 7.30 (d, *J*= 2.5 Hz, 1H), 7.43 (t, *J*= 8.7 Hz, 1H), 7.75 (t, *J*= 7.5 Hz, 2H), 12.69 (s, NH); ^{13}C NMR (100 MHz, DMSO-d_6): δ (ppm) 30.2, 31.6, 34.6, 35.2, 55.1, 105.1, 111.5, 114.7, 115.0, 118.6, 129.9, 131.9, 135.3, 139.9, 140.1, 144.7, 148.7, 159.5. Anal. Calcd for $C_{22}H_{28}N_2O$: C, 78.53; H, 8.39; N, 8.33; Found: C, 78.45; H, 8.30; N, 8.40.

2-(3-bromophenyl)-4,6-di-*tert*-butyl-1*H*-benzo[*d*]imidazole(4m). Purified by column chromatography on silica gel and eluted with ethyl acetate/petroleum ether (5:100). Isolated yield: 335 mg, 87%. Colorless liquid; ^{1}H NMR (250 MHz, DMSO-d_6): δ (ppm) 1.32 (s, 9H), 1.55 (s, 9H), 7.09 (s, 1H), 7.29 (s, 1H), 7.47 (t, *J*= 7.5 Hz, 1H), 7.64 (d, *J*= 7.5 Hz, 1H), 8.17 (d, *J*= 7.5 Hz, 1H), 8.33 (s, 1H), 12.98 (s, NH); ^{13}C NMR (100 MHz, DMSO-d_6): δ (ppm) 30.2, 31.6, 34.6, 35.2, 105.3, 115.3, 122.1, 125.1, 128.4, 131.0, 131.9, 132.8, 135.4, 139.8, 140.3, 145.1, 147.2. Anal. Calcd for $C_{21}H_{25}BrN_2$: C, 65.46; H, 6.54; N, 7.27; Found: C, 65.35; H, 6.41; N, 7.12.

4,6-di-*tert*-butyl-2-(3,4-dimethoxyphenyl)-1*H*-benzo[*d*]imidazole (4n). Purified by column chromatography on silica gel and eluted with ethyl acetate/petroleum ether (5:100). Isolated yield: 340 mg, 93%. Colorless liquid; ^{1}H NMR (250 MHz, DMSO-d_6): δ (ppm) 1.34 (s, 9H), 1.56 (s, 9H), 3.81 (s, 3H), 3.86 (s, 3H), 7.06-7.11 (m, 2H), 7.27 (s, 1H), 7.73 (d, *J*= 5.0 Hz, 2H), 12.50 (s, NH); ^{13}C NMR (100 MHz, DMSO-d_6): δ (ppm) 35.0, 36.4, 39.3, 39.9, 60.3, 60.4, 109.6, 114.3, 116.5, 119.5, 123.9, 128.0, 140.0, 144.5, 144.7, 148.9, 153.5, 154.6. Anal. Calcd for $C_{23}H_{30}N_2O_2$: C, 75.37; H, 8.25; N, 7.64; Found: C, 75.08; H, 8.51; N, 7.58.

4,6-di-*tert*-butyl-2-(2,3-dimethoxyphenyl)-1*H*-benzo[*d*]imidazole (4o). Purified by column chromatography on silica gel and eluted with ethyl acetate/petroleum ether (5:100). Isolated yield: 329 mg, 90%. Cream solid, mp: 123-125 ^{o}C; ^{1}H NMR (250 MHz, DMSO-d_6): δ (ppm) 1.33 (s, 9H), 1.55 (s, 9H), 3.84, 3.86 (2s, 6H), 7.07 (d, *J*= 2.5 Hz, 1H), 7.11-7.22 (m, 2H), 7.45 (d, *J*= 2.5 Hz, 1H), 7.82 (d, *J*= 7.5 Hz, 1H), 11.97 (s, NH); ^{13}C NMR (100 MHz, DMSO-d_6): δ (ppm) 35.5, 36.9, 39.9, 40.4, 61.1, 65.9, 111.3, 118.9, 120.2, 126.3, 129.1, 129.5, 140.4, 144.2, 145.0, 149.6, 151.4, 151.8, 158.1. Anal. Calcd for $C_{23}H_{30}N_2O_2$: C, 75.37; H, 8.25; N, 7.64; Found: C, 75.23; H, 8.13; N, 7.52.

4,6-di-*tert*-butyl-2-(2,6-dichlorophenyl)-1*H*-benzo[*d*]imidazole (4p). Purified by column chromatography on silica gel and eluted with ethyl acetate/petroleum ether (5:100). Isolated yield: 315 mg, 84%. White solid, mp: 243-244 ^{o}C; ^{1}H NMR (250 MHz, DMSO-d_6): δ (ppm) 1.34 (s, 9H), 1.51 (s, 9H), 7.11 (d, *J*= 2.5 Hz, 1H), 7.28 (d, *J*= 2.5 Hz, 1H), 7.53-7.66 (m, 3H), 12.38 (s, NH); ^{13}C NMR (100 MHz, DMSO-d_6): δ (ppm) 30.1, 31.6, 34.6, 35.1, 105.4, 114.9, 128.3, 130.8, 132.0, 134.4, 135.1, 139.3, 140.4, 144.0, 144.9. Anal. Calcd for $C_{21}H_{24}Cl_2N_2$: C, 67.20; H, 6.45; N, 7.46; Found: C, 66.80; H, 6.14; N, 8.26.

4,6-di-*tert*-butyl-2-phenyl-1*H*-benzo[*d*]imidazole (4q). Purified by column chromatography on silica gel and eluted with ethyl acetate/petroleum ether (5:100). Isolated yield: 275 mg, 90%. Colorless liquid; ^{1}H NMR (250 MHz, DMSO-d_6): δ (ppm) 1.11 (s, 9H), 1.34 (s, 9H), 6.85 (d, *J*= 2.5 Hz, 1H), 7.07 (d, *J*= 2.5 Hz, 1H), 7.20-7.31 (m, 3H), 7.93 (d, *J*= 7.5 Hz, 2H), 12.47 (s, NH); ^{13}C NMR (100 MHz, DMSO-d_6): δ (ppm) 30.7, 32.1, 35.1, 35.7, 105.7, 115.5, 126.6, 129.3, 129.7, 131.0, 135.9, 140.4, 140.6, 145.2, 149.3. Anal. Calcd for $C_{21}H_{26}N_2$: C, 82.31; H, 8.55; N, 9.14; Found: C, 82.42; H, 8.67; N, 9.28.

4,6-di-*tert*-butyl-2-(thiophen-2-yl)-1*H*-benzo[*d*]imidazole (4r). Purified by column chromatography on silica gel and eluted with ethyl acetate/petroleum ether (10:100). Isolated yield: 281 mg, 90%. White solid, mp: 182-183 °C; ^{1}H NMR (250 MHz, DMSO-d_6): δ (ppm) 1.33 (s, 9H), 1.53 (s, 9H), 7.07 (d, *J*= 2.5 Hz, 1H), 7.17-7.20 (m, 1H), 7.24 (d, *J*= 2.5 Hz, 1H), 7.65 (d, *J*= 5.0 Hz, 1H), 7.73 (d, *J*= 2.5 Hz, 1H), 12.69 (s, NH); ^{13}C NMR (100 MHz, DMSO-d_6): δ (ppm) 30.2, 31.6, 34.6, 35.1, 105.0, 115.1, 125.8, 127.9, 128.0, 134.2, 135.0, 139.7, 139.9, 144.7, 144.8. Anal. Calcd for $C_{19}H_{24}N_2S$: C, 73.03; H, 7.74; N, 8.97; Found: C, 72.81; H, 7.56; N, 8.85.

4,6-di-*tert*-butyl-2-(thiophen-3-yl)-1*H*-benzo[*d*]imidazole (4s). Purified by column chromatography on silica gel and eluted with ethyl acetate/petroleum ether (10:100). Isolated yield: 265 mg, 85%. White solid, mp: 200-201 °C; ^{1}H NMR (250 MHz, DMSO-d_6): δ (ppm) 1.33 (s, 9H), 1.55 (s, 9H), 7.07 (d, *J*= 2.5 Hz, 1H), 7.26 (d, *J*= 2.5 Hz, 1H), 7.67 (d, *J*= 7.5 Hz, 1H), 7.74 (d, *J*= 5.0 Hz, 1H), 8.12 (s, 1H), 12.55 (s, NH); ^{13}C NMR (100 MHz, DMSO-d_6): δ (ppm) 30.2, 31.6, 34.6, 35.1, 105.0, 114.9, 123.9, 126.3, 127.2, 132.9, 134.8, 139.7, 140.0, 144.5, 145.7. Anal. Calcd for $C_{19}H_{24}N_2S$: C, 73.03; H, 7.74; N, 8.97; Found: C, 73. 15; H, 7.80; N, 9.14.

4,6-di-*tert*-butyl-2-(naphthalen-1-yl)-1*H*-benzo[*d*]imidazole (4t). Purified by column chromatography on silica gel and eluted with ethyl acetate/petroleum ether (5:100). Isolated yield: 295 mg, 83%. Colorless liquid; ^{1}H NMR (250 MHz, DMSO-d_6): δ (ppm) 1.35 (s, 9H), 1.60 (s, 9H), 7.14 (s, 1H), 7.35 (s, 1H), 7.57-7.69 (m, 3H), 7.96-8.07 (m, 3H), 9.14 (d, *J*= 10.0 Hz, 1H), 12.79 (s, NH); ^{13}C NMR (100 MHz, DMSO-d_6): δ (ppm) 35.5, 36.9, 39.9, 40.4, 110.5, 120.2, 130.5, 131.4, 132.1, 132.7, 133.6, 134.9, 135.7, 138.9, 140.0, 145.1, 145.4, 150.1, 154.1. Anal. Calcd for $C_{25}H_{28}N_2$: C, 84.23; H, 7.92; N, 7.86; Found: C, 84.01; H, 7.70; N, 7.72.

4,6-di-*tert*-butyl-2-(phenanthren-9-yl)-1*H*-benzo[*d*]imidazole (4u). Purified by column chromatography on silica gel and eluted with ethyl acetate/petroleum ether (5:100). Isolated yield: 313 mg, 77%. Colorless liquid; ^{1}H NMR (400 MHz, DMSO-d_6): δ (ppm) 1.40 (s, 9H), 1.64 (s, 9H), 7.19 (d, *J*= 4.0 Hz, 1H), 7.39 (d, *J*= 4.0 Hz, 1H), 7.71-7.81 (m, 4H), 8.10-87.13 (m, 1H), 8.33 (s, 1H), 8.92-8.99 (m, 2H), 9.15-9.18 (m, 1H), 12.84 (s, NH); ^{13}C NMR (100 MHz, DMSO-d_6): δ (ppm) 30.3, 31.7, 34.7, 35.2, 105.2, 115.0, 122.9, 123.2, 126.8, 127.0, 127.1, 127.3, 127.9, 128.7, 129.0, 129.2, 130.0, 130.3, 130.4, 134.7, 139.9, 140.3, 144.9, 148.8. Anal. Calcd for $C_{29}H_{30}N_2$: C, 85.67; H, 7.44; N, 6.89; Found: C, 85.47; H, 7.31; N, 6.80.

4,6-di-*tert*-butyl-2-mesityl-1*H*-benzo[*d*]imidazole (4v). Purified by column chromatography on silica gel and eluted with ethyl acetate/petroleum ether (5:100). Isolated yield: 278 mg, 80%. Colorless liquid; ^{1}H NMR (400 MHz, DMSO-d_6): δ (ppm) 1.36 (s, 9H), 1.54 (s, 9H), 2.11 (s, 6H), 2.32 (s, 3H), 7.01 (s, 2H), 7.09 (d, *J*= 4.0 Hz, 1H), 7.26 (d, *J*= 4.0 Hz, 1H), 12.19 (s, NH); ^{13}C NMR (100 MHz, DMSO-d_6): δ (ppm) 19.7, 19.8, 20.7, 30.2, 31.7, 34.5, 35.1, 105.0, 114.5, 128.0, 129.1, 134.4, 137.1, 138.1, 139.5, 139.9, 144.0, 148.5. Anal. Calcd for $C_{24}H_{32}N_2$: C, 82.71; H, 9.25; N, 8.04; Found: C, 82.55; H, 9.11; N, 8.13.

3-(4,6-di-*tert*-butyl-1*H*-benzo[*d*]imidazol-2-yl)quinolin-2(1*H*)-one (4w). Purified by column chromatography on silica gel and eluted with ethyl acetate/petroleum ether (20:100). Isolated yield: 186 mg, 50%. Yellow solid; mp: 217-220 °C; ^{1}H NMR (400 MHz, DMSO-d_6): δ (ppm) 1.14 (s, 9H), 1.28 (s, 9H), 7.03 (t, *J*= 8.0 Hz, 1H), 7.09 (s, 1H), 7.11-7.19 (m, 2H), 7.40 (t, *J*= 8.0 Hz, 2H), 7.70 (d, *J*= 8.0 Hz, 1H), 8.59 (s, 1H), 11.97 (s, NH); ^{13}C NMR (100 MHz, $CDCl_3$): δ (ppm) 30.0, 31.7, 34.5, 35.0, 114.6, 116.5, 119.0, 120.1, 123.0, 128.7, 130.4, 132.4, 133.6, 133.7, 139.5, 142.0, 143.0, 147.9, 159.3, 189.5. Anal. Calcd for $C_{24}H_{27}N_3O$: C, 77.18; H, 7.29; N, 11.25; Found: C, 77.01; H, 7.17; N, 11.16.

4,6-di-*tert*-butyl-2-(3-(prop-2-yn-1-yloxy)phenyl)-1*H*-benzo[*d*]imidazole (4x). Purified by column chromatography on silica gel and eluted with ethyl acetate/petroleum ether (10:100). Isolated yield: 299 mg, 83%. Colorless liquid; ^{1}H NMR (400 MHz, DMSO-d_6): δ(ppm) 1.35 (s, 9H), 1.57 (s, 9H), 3.44 (t, *J*= 6.0 Hz, 1H), 4.89 (d,*J*= 4.0 Hz, 2H), 7.09-7.11 (m, 2H), 7.31 (d, *J*= 4.0 Hz, 1H), 7.48 (t, *J*= 8.0 Hz, 1H), 7.74-7.79 (m, 2H), 12.72 (s, NH); ^{13}C NMR (100 MHz, DMSO-d_6): δ (ppm) 30.2, 31.6, 34.6, 35.1, 55.5, 78.3, 79.0, 105.2, 112.7, 115.1, 115.4, 119.2, 130.0, 131.8, 135.3, 139.8, 140.2, 144.9, 148.5, 157.4. Anal. Calcd for $C_{24}H_{28}N_2O$: C, 79.96; H, 7.83; N, 7.77; Found: C, 80.08; H, 7.98; N, 7.60.

4,6-di-*tert*-butyl-2-(4-(prop-2-yn-1-yloxy)phenyl)-1*H*-benzo[*d*]imidazole (4y). Purified by column chromatography on silica gel and eluted with ethyl acetate/petroleum ether (10:100). Isolated yield: 324 mg, 90%. Colorless liquid; ^{1}H NMR (400 MHz, DMSO-d_6): δ (ppm) 1.36 (s, 9H), 1.58 (s, 9H), 3.63 (t, *J*= 4.0 Hz, 1H), 4.89 (d, *J*= 4.0 Hz, 2H), 7.08 (d, *J*= 4.0 Hz, 1H), 7.16 (d, *J*= 8.0 Hz, 2H), 7.28 (d, *J*= 4.0 Hz, 1H), 8.09 (d, *J*= 8.0 Hz, 2H), 12.57 (s, NH). Anal. Calcd for $C_{24}H_{28}N_2O$: C, 79.96; H, 7.83; N, 7.77; Found: C, 79.88; H, 7.62; N, 7.85.

2-(3-(allyloxy)phenyl)-4,6-di-*tert*-butyl-1*H*-benzo[*d*]imidazole (4z). Purified by column chromatography on silica gel and eluted with ethyl acetate/petroleum ether (10:100). Isolated yield: 290 mg, 80%.Colorless liquid; ^{1}H NMR (400 MHz, DMSO-d_6): δ (ppm) 1.36 (s, 9H), 1.58 (s, 9H), 4.67 (d, *J*= 8.0 Hz, 2H), 5.31 (dd, *J*= 4.0, 12.0 Hz, 1H), 5.46 (dd, *J*= 4.0, 16.0 Hz, 1H), 6.06-6.15 (m, 1H), 7.04-7.07 (m, 1H), 7.10 (d, *J*= 4.0 Hz, 1H), 7.31 (d, *J*= 4.0 Hz, 1H), 7.45 (t, *J*= 8.0 Hz, 1H), 7.74 (d, *J*= 4.0

Hz, 1H), 7.77 (s, 1H), 12.71 (s, NH); ^{13}C NMR (100 MHz, DMSO-d_6): δ (ppm) 30.24, 31.63, 35.20, 36.17, 68.30, 105.21, 112.41, 115.09, 115.44, 117.60, 118.72, 130.01, 131.87, 133.56, 135.35, 139.87, 140.19, 144.79, 148.67, 158.46. Anal. Calcd for $C_{24}H_{30}N_2O$: C, 79.52; H, 8.34; N, 7.73; Found: C, 79.34; H, 8.16; N, 7.62.

2-(4-(allyloxy)phenyl)-5,7-di-*tert*-butyl-1*H*-benzo[*d*]imidazole (4aa). Purified by column chromatography on silica gel and eluted with ethyl acetate/petroleum ether (10:100). Isolated yield: 308 mg, 85%. Colorless liquid; ^{1}H NMR (400 MHz, DMSO-d_6): δ (ppm) 1.35 (s, 9H), 1.58 (s, 9H), 4.64 (d, J= 4.0 Hz, 2H), 5.30 (dd, J= 4.0, 12.0 Hz, 1H), 5.44 (dd, J= 4.0, 16.0 Hz, 1H), 6.03-6.13 (m, 1H), 7.08 (d, J= 4.0 Hz, 1H), 7.11 (d, J= 8.0 Hz, 2H), 7.28 (d, J= 4.0 Hz, 1H), 8.09 (d, J= 8.0 Hz, 2H), 12.58 (s, NH); ^{13}C NMR (100 MHz, DMSO-d_6): δ (ppm) 30.2, 31.6, 34.5, 35.1, 68.2, 105.0, 114.8, 114.9, 117.6, 123.3, 127.6, 133.4, 135.3, 139.7, 139.9, 144.2, 148.9, 159.1. Anal. Calcd for $C_{24}H_{30}N_2O$: C, 79.52; H, 8.34; N, 7.73; Found: C, 79.38; H, 8.40; N, 7.78.

4-((4,6-di-*tert*-butyl-2-(4-chlorophenyl)-1*H*-benzo[*d*]imidazol-7-yl)methyl)morpholine (4ab). Purified by column chromatography on silica gel and eluted with ethyl acetate/petroleum ether (10:100). Isolated yield: 378 mg, 86%. Colorless liquid; ^{1}H NMR (400 MHz, DMSO-d_6): δ (ppm) 1.47 (s, 9H), 1.57 (s, 9H), 2.47 (t, J= 6.0 Hz, 4H), 3.59 (t, J= 6.0 Hz, 4H), 4.07 (s, 2H), 7.16 (s, 1H), 7.65 (d, J= 8.0 Hz, 2H), 8.21 (d, J= 8.0 Hz, 2H), 12.05 (s, NH); ^{13}C NMR (100 MHz, DMSO-d_6): δ (ppm) 30.2, 32.2, 35.1, 36.0, 52.5, 56.7, 66.6, 116.7, 117.0, 128.2, 128.9, 129.2, 134.0, 136.8, 138.5, 139.9, 142.0, 147.3. Anal. Calcd for $C_{26}H_{34}ClN_3O$: C, 70.97; H, 7.79; N, 9.55; Found: C, 70.85; H, 7.62; N, 9.64.

4-((4,6-di-*tert*-butyl-2-(*p*-tolyl)-1*H*-benzo[*d*]imidazol-7-yl)methyl)morpholine (4ac). Purified by column chromatography on silica gel and eluted with ethyl acetate/petroleum ether (10:100). Isolated yield: 344 mg, 82%. Colorless liquid; ^{1}H NMR (400 MHz, DMSO-d_6): δ (ppm) 1.39 (s, 9H), 1.49 (s, 9H), 2.33 (s, 3H), 2.41 (s, 4H), 3.52 (t, J= 4.0 Hz, 4H), 4.00 (s, 2H), 7.06 (s, 1H), 7.31 (d, J= 8.0 Hz, 2H), 7.99 (d, J= 8.0 Hz, 2H), 11.82 (s, NH); ^{13}C NMR (100 MHz, $CDCl_3$): δ (ppm) 21.6, 30.0, 32.5, 34.4, 37.5, 53.2, 54.7, 67.3, 121.5, 125.0, 125.0, 127.4, 129.5, 132.1, 141.4, 144.9, 145.7, 145.8, 161.4. Anal. Calcd for $C_{27}H_{37}N_3O$: C, 77.28; H, 8.89; N, 10.01; Found: C, 77.03; H, 8.81; N, 9.94.

4-(4,6-di-*tert*-butyl-7-(morpholinomethyl)-1*H*-benzo[*d*]imidazol-2-yl)-2-methoxyphenol (4ad). Purified by column chromatography on silica gel and eluted with ethyl acetate/petroleum ether (15:100). Isolated yield: 338 mg, 75%. Colorless liquid; ^{1}H NMR (400 MHz, DMSO-d_6): δ (ppm) 1.46 (s, 9H), 1.57 (s, 9H), 2.48 (s, 4H), 3.60 (t, J= 6.0 Hz, 4H), 3.91 (s, 3H), 4.07 (s, 2H), 6.96 (d, J= 8.0 Hz, 1H), 7.11 (s, 1H), 7.61 (d, J= 8.0 Hz, 1H), 7.66 (s, 1H), 9.54 (s, OH), 11.79 (s, NH); ^{13}C NMR (100 MHz, DMSO-d_6): δ (ppm) 30.2, 32.2, 35.1, 35.8, 52.6, 55.6, 57.1, 66.7, 110.4, 115.8, 116.0, 116.4, 119.7, 136.5, 137.9, 140.1, 141.0, 147.7, 148.9. Anal. Calcd for $C_{27}H_{37}N_3O_3$: C, 71.81; H, 8.26; N, 9.30; Found: C, 71.90; H, 8.10; N, 9.38.

2-(4-chlorophenyl)-5-methyl-1*H*-benzo[*d*]imidazole (4ae). Purified by column chromatography on silica gel and eluted with ethyl acetate/petroleum ether (5:100). Isolated yield: 145 mg, 60%. White solid, mp: 229-230 ^{o}C; ^{1}H NMR (400 MHz, DMSO-d_6): δ (ppm) 2.42 (s, 3H), 7.03 (d, J= 8.0 Hz, 1H), 7.39 (s, 1H), 7.50 (d, J= 8.0 Hz, 1H), 7.60 (d, J= 8.0 Hz, 2H), 8.19 (d, J= 8.0 Hz, 2H), 12.87 (s, NH); ^{13}C NMR (100 MHz, DMSO-d_6): δ (ppm) 21.9, 110.8, 117.7, 123.7, 127.9, 128.9, 129.1, 131.5, 134.2, 149.8. Anal. Calcd for $C_{14}H_{11}ClN_2$: C, 69.28; H, 4.57; N, 11.54; Found: C, 69.42; H, 4.40; N, 11.47.

4,6-di-*tert*-butyl-2-methyl-1*H*-benzo[*d*]imidazole (4af). Purified by column chromatography on silica gel and eluted with ethyl acetate/petroleum ether (5:100). Isolated yield: 171 mg, 70%. brown liquid; ^{1}H NMR (400 MHz, DMSO-d_6): δ (ppm) 1.32 (s, 9H), 1.48 (s, 9H), 2.47 (s, 3H), 7.02 (d, J= 4.0 Hz, 1H), 7.21 (d, J= 4.0 Hz, 1H), 11.99 (s, NH); ^{13}C NMR (100 MHz, DMSO-d_6): δ (ppm) 13.9, 30.2, 31.7, 34.8, 34.9, 109.3, 117.6, 131.1, 135.9, 138.2, 146.1, 149.8. Anal. Calcd for $C_{16}H_{24}N_2$: C, 78.64; H, 9.90; N, 11.46; Found: C, 78.57; H, 9.98; N, 11.50.

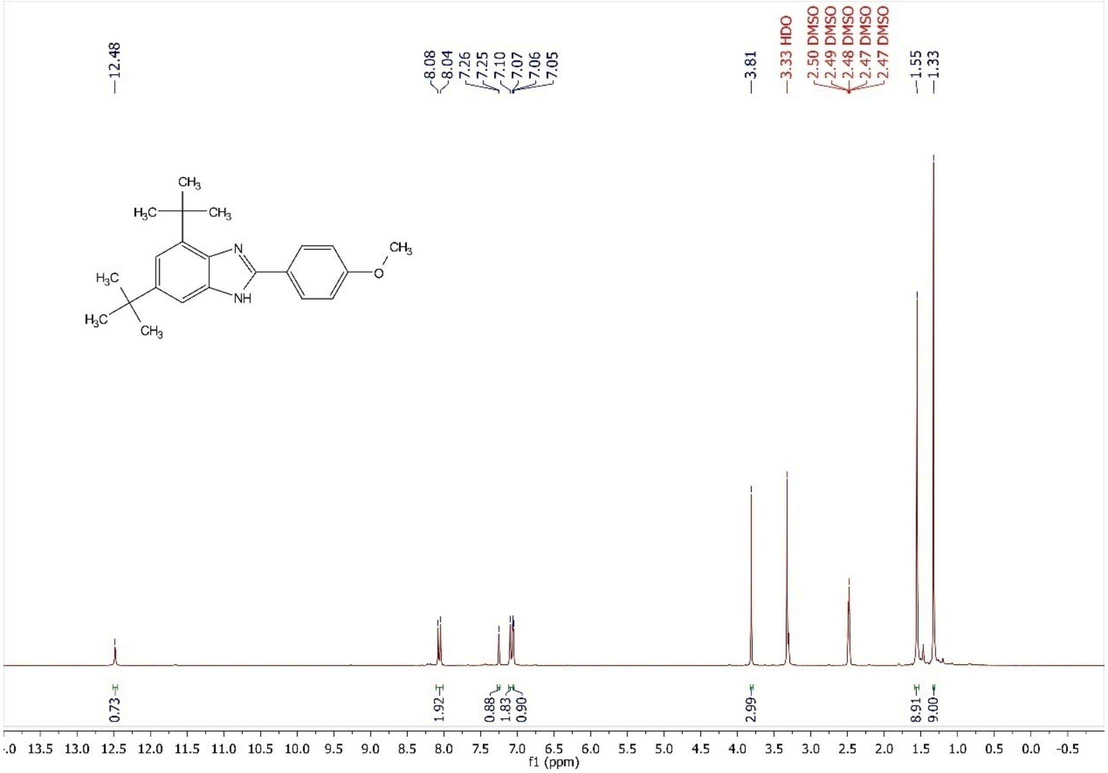

Figure S1. ^{1}H **NMR spectrum of compound 4a in DMSO-d_6 (250 MHz).**

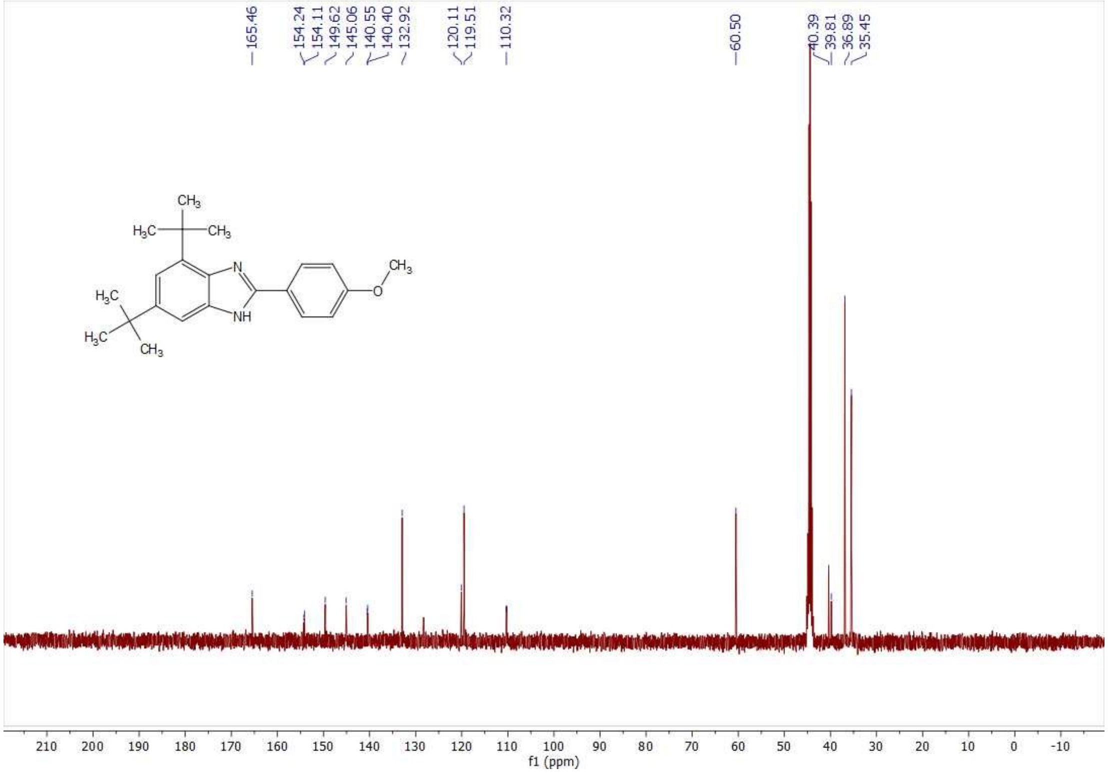

Figure S2. ^{13}C **NMR spectrum of compound 4a in DMSO-d_6 (100 MHz).**

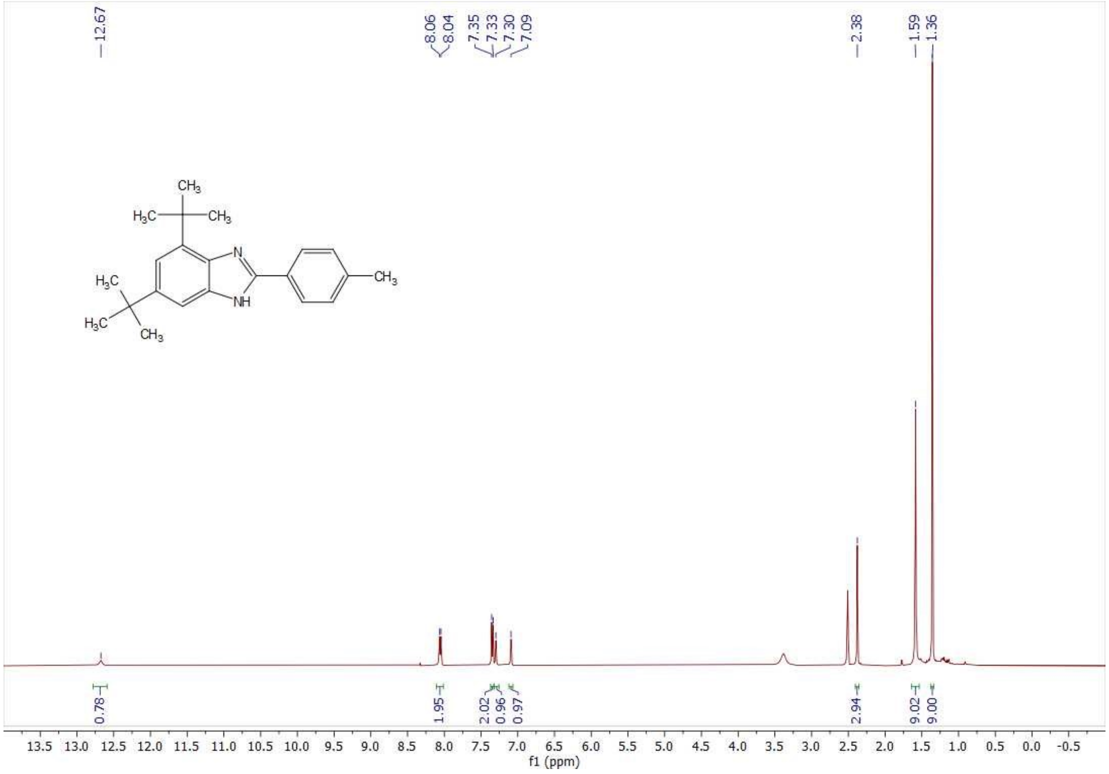

Figure S3. ^{1}H NMR spectrum of compound 4b in DMSO-d_6 (400MHz).

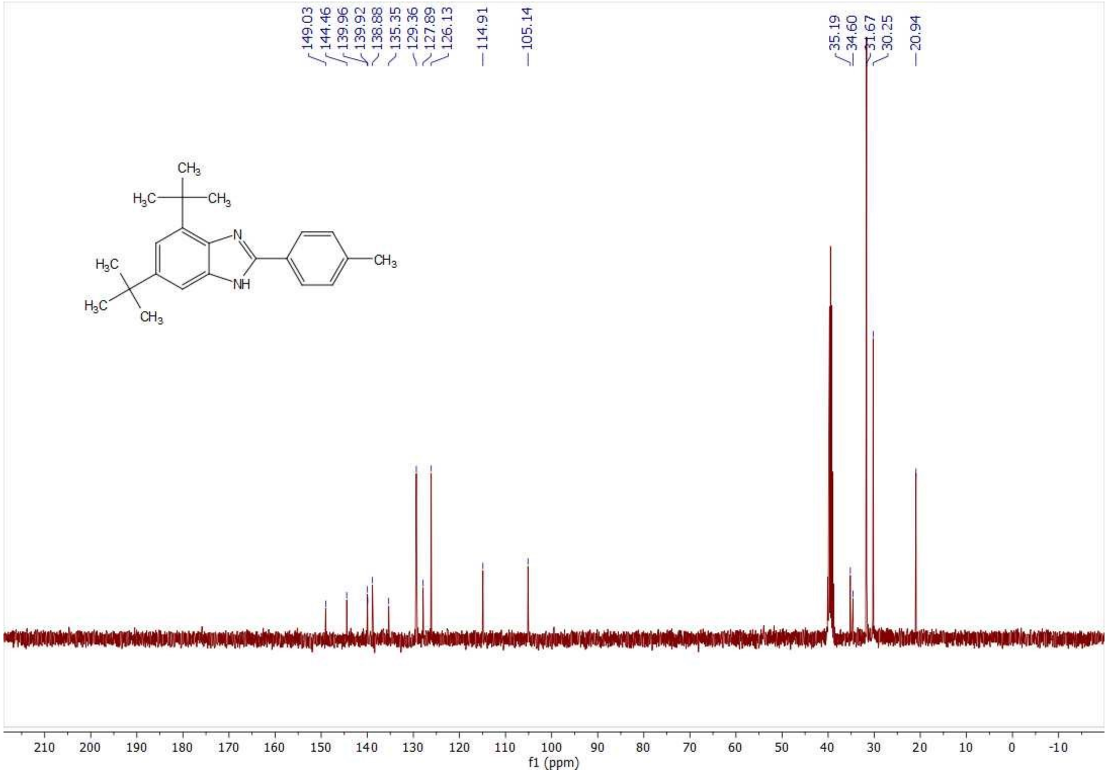

Figure S4. ^{13}C NMR spectrum of compound 4b in DMSO-d_6 (100 MHz).

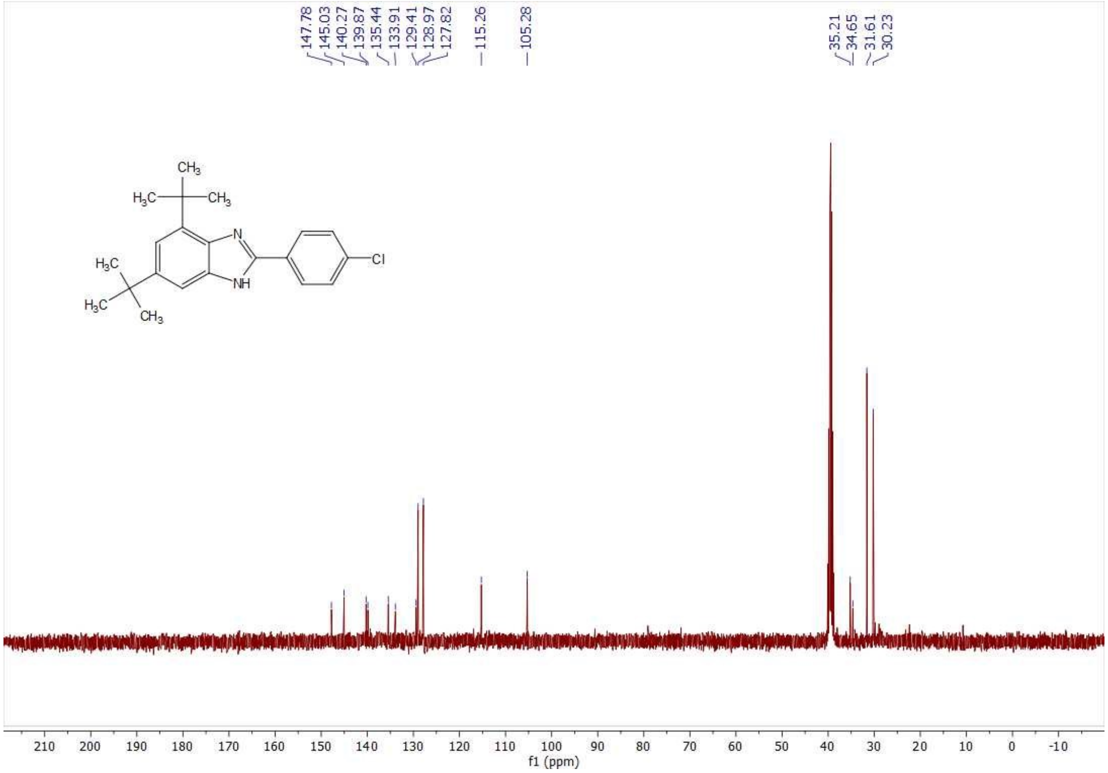

Figure S5. ^{1}H NMR spectrum of compound 4c in DMSO-d_6 (250 MHz).

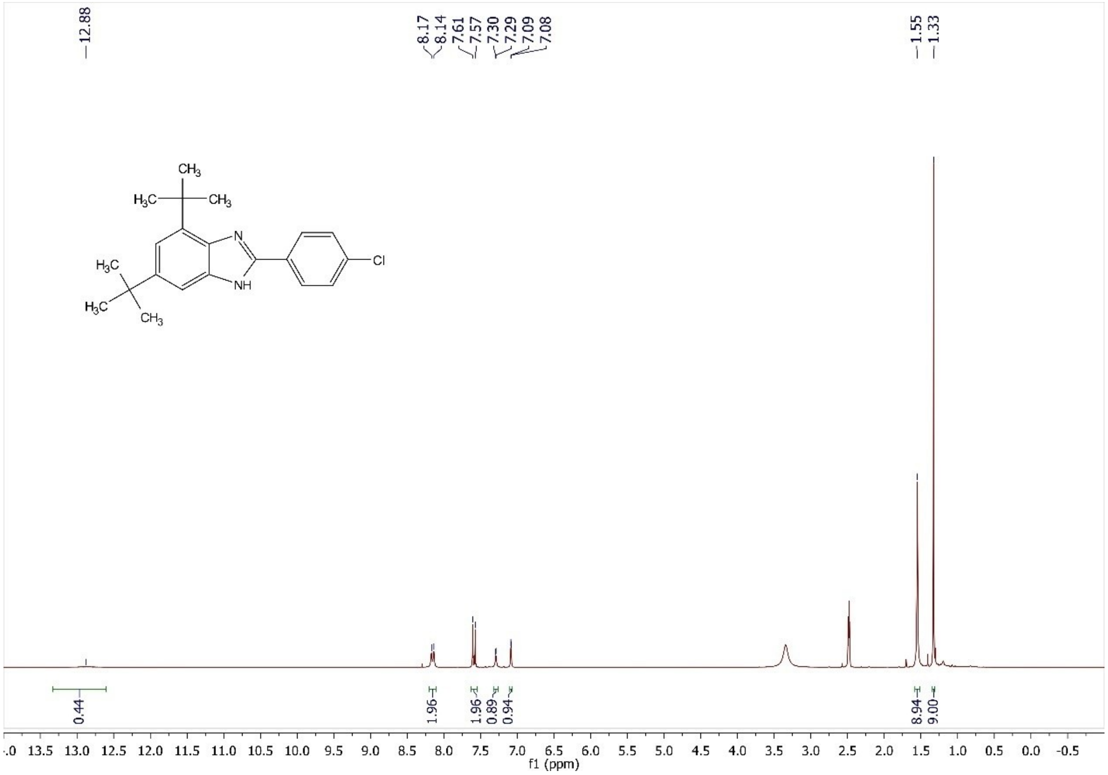

Figure S6. ^{13}C NMR spectrum of compound 4c in DMSO-d_6 (100 MHz).

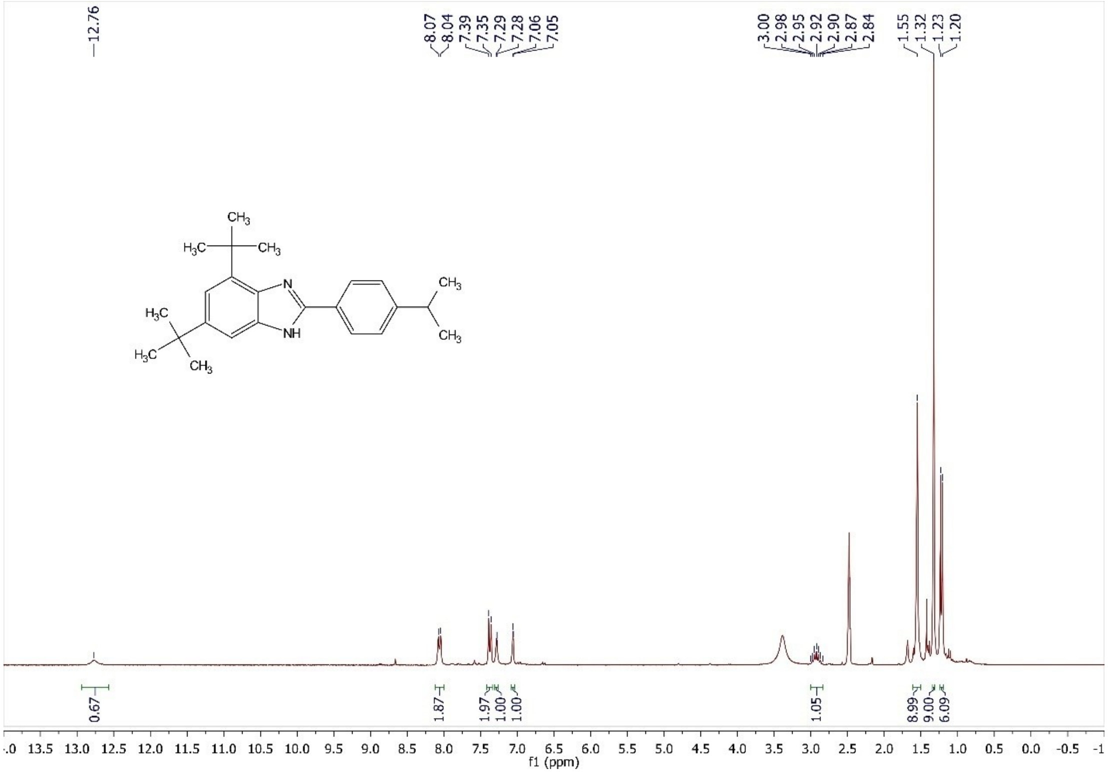

Figure S7. ^{1}H NMR spectrum of compound 4d in DMSO-d_6 (250 MHz).

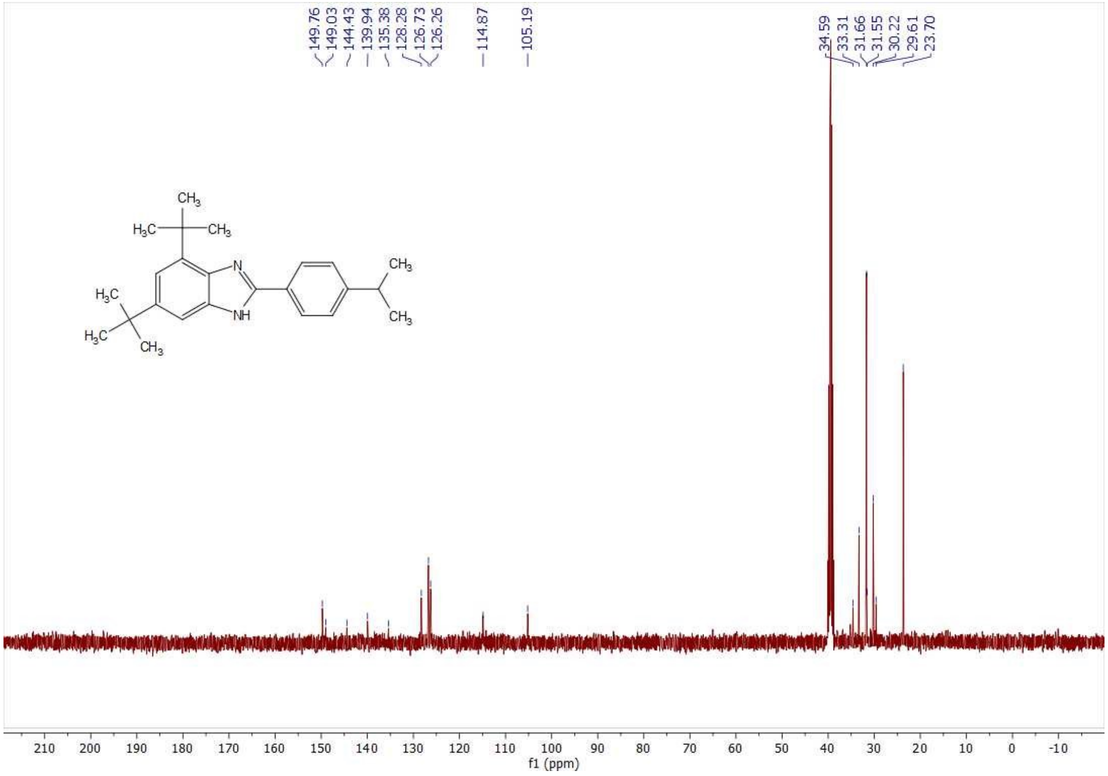

Figure S8. ^{13}C NMR spectrum of compound 4d in DMSO-d_6 (100 MHz).

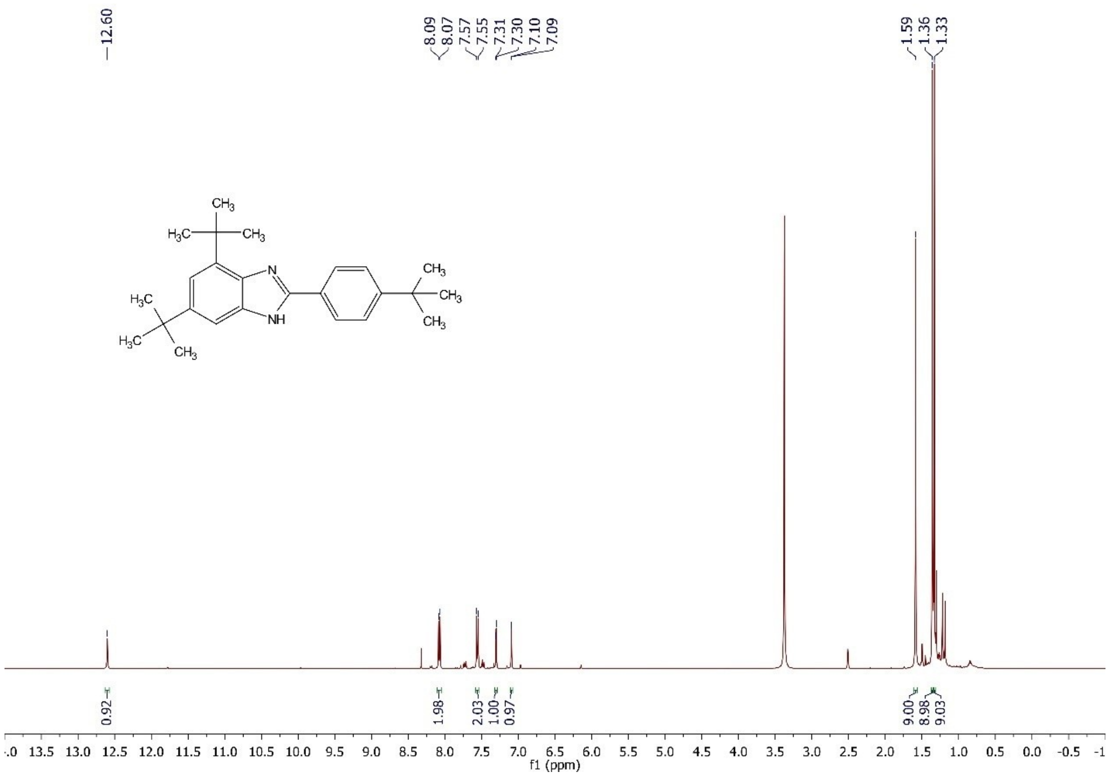

Figure S9. ^{1}H NMR spectrum of compound 4e in DMSO-d_6 (400MHz).

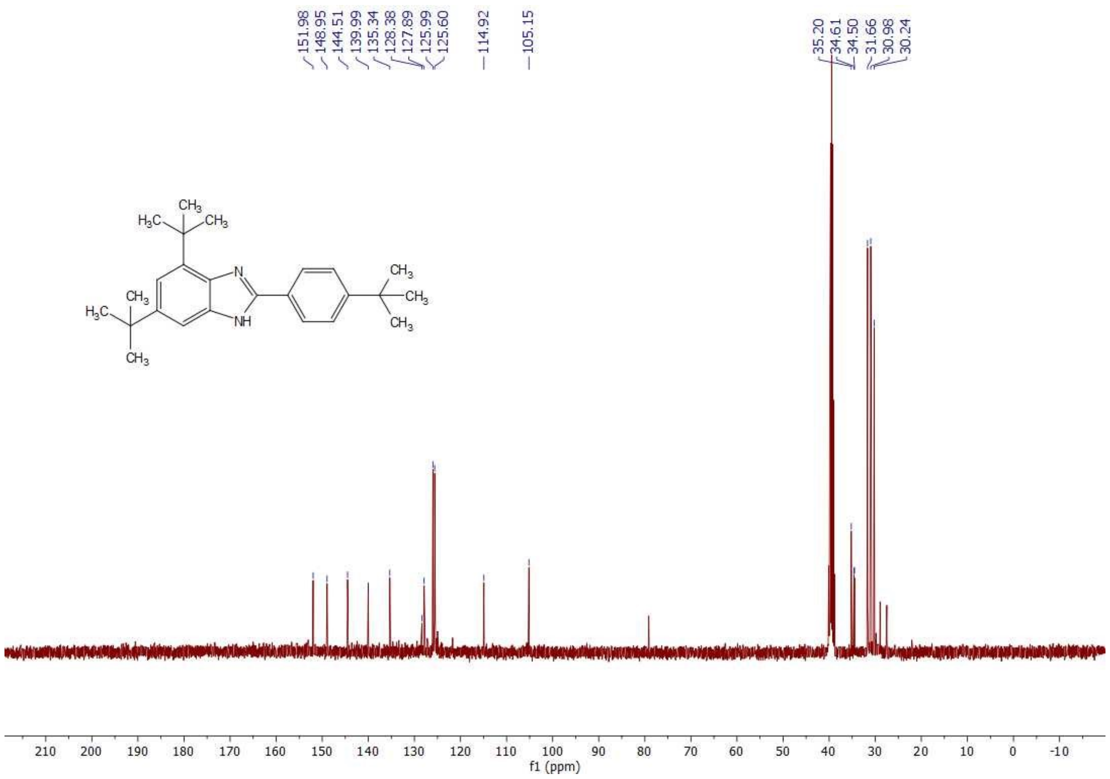

Figure S10. ^{13}C NMR spectrum of compound 4e in DMSO-d_6 (100 MHz).

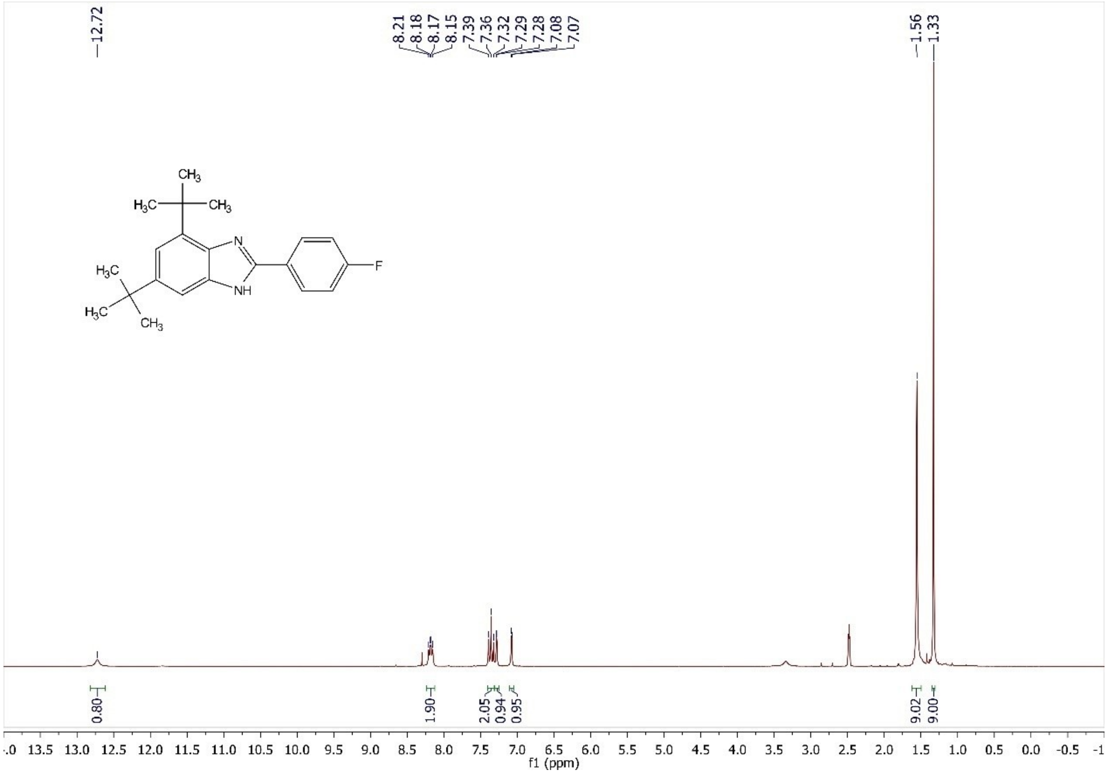

Figure S11. ^{1}H NMR spectrum of compound 4f in DMSO-d_6 (250 MHz).

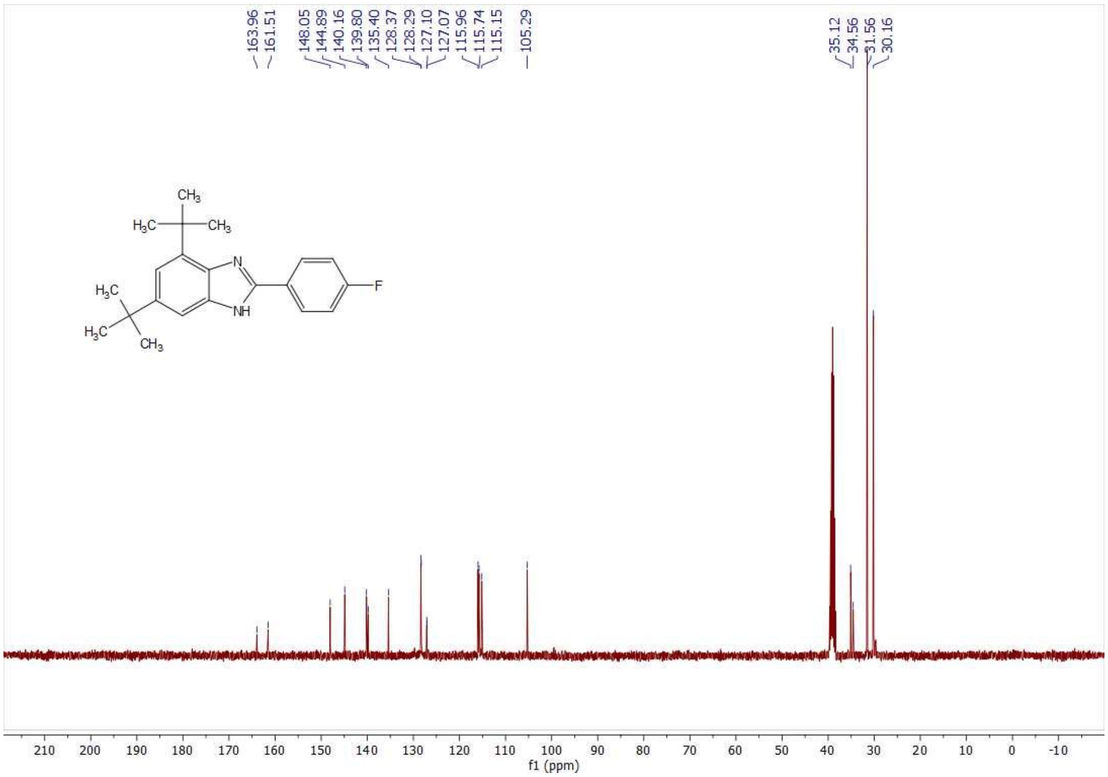

Figure S12. ^{13}C NMR spectrum of compound 4f in DMSO-d_6 (100 MHz).

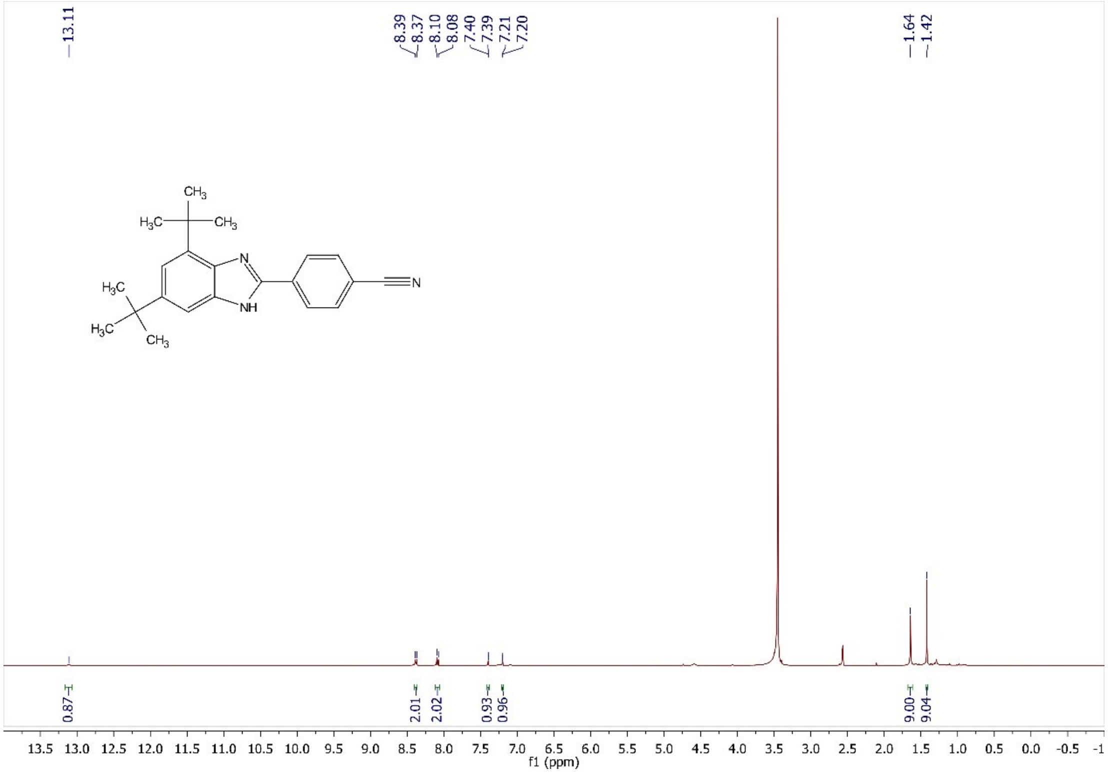

Figure S13. ^{1}H NMR spectrum of compound 4g in DMSO-d_6 (400 MHz).

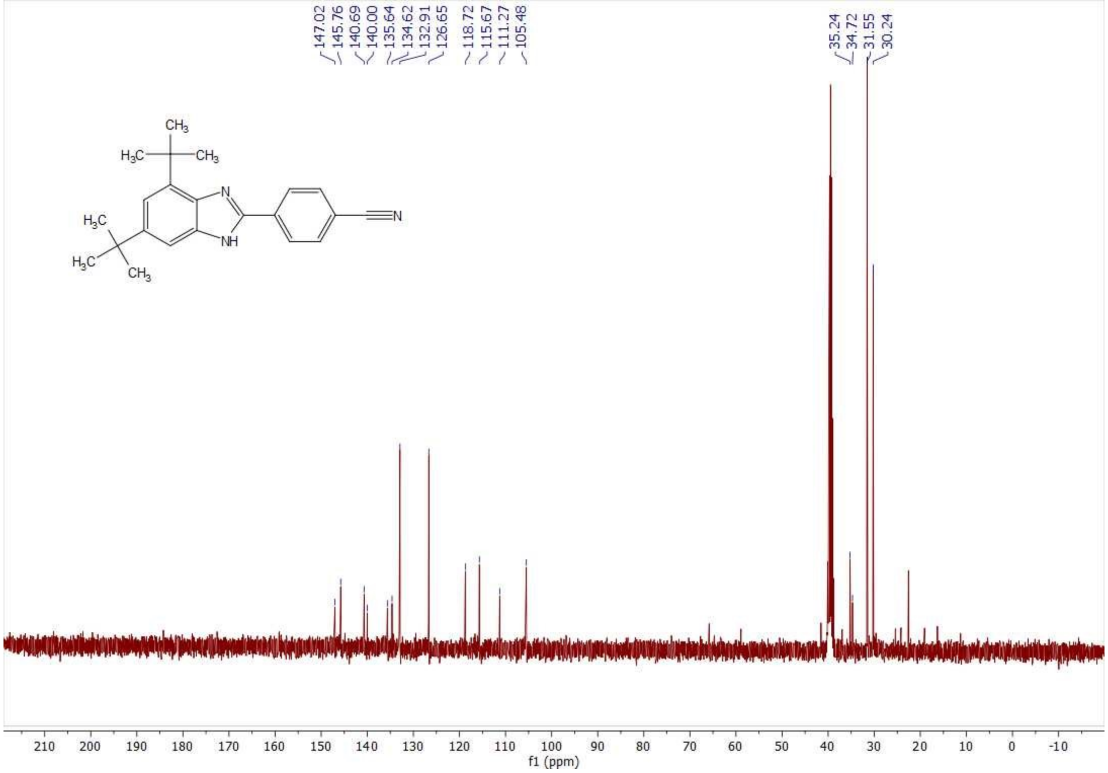

Figure S14. ^{13}C NMR spectrum of compound 4g in DMSO-d_6 (100 MHz).

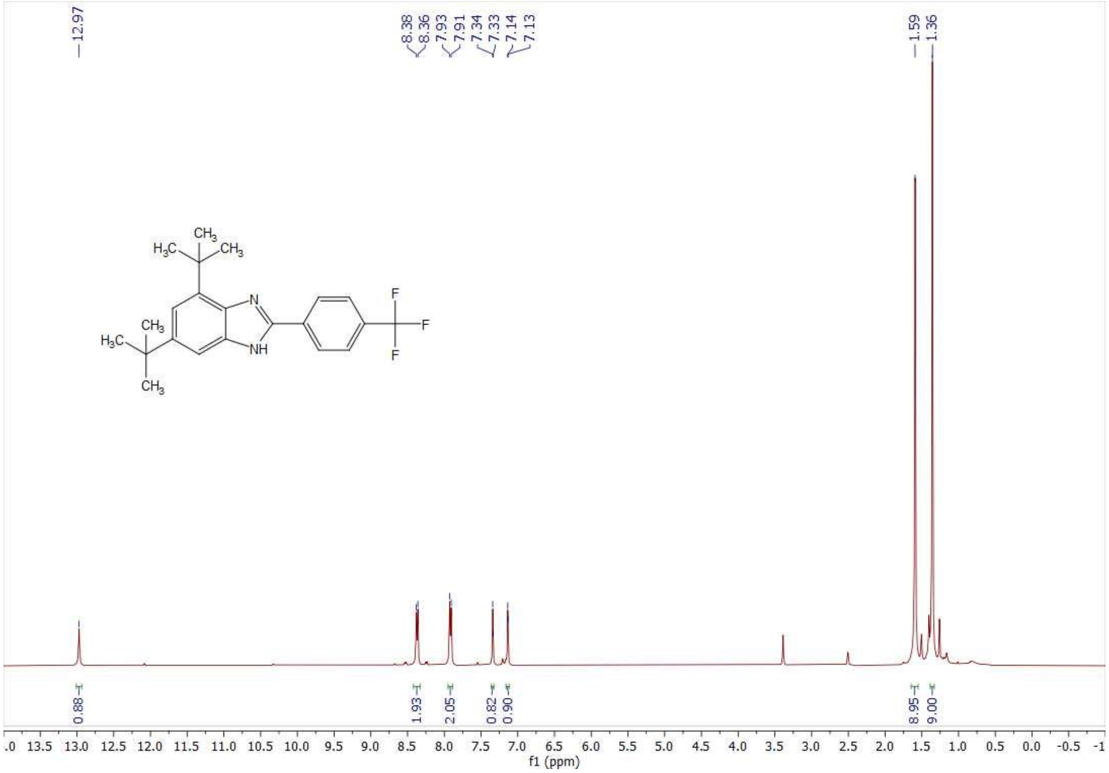

Figure S15. ^{1}H NMR spectrum of compound 4h in DMSO-d_6 (400 MHz).

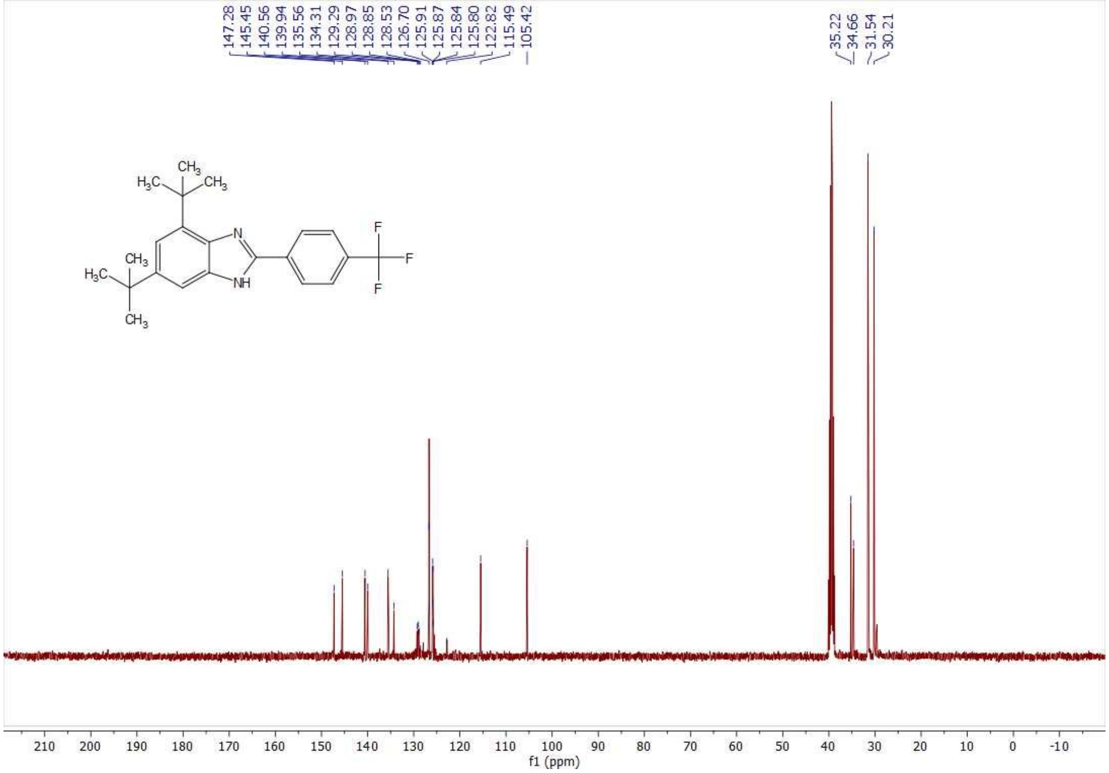

Figure S16. ^{13}C NMR spectrum of compound 4h in DMSO-d_6 (100 MHz).

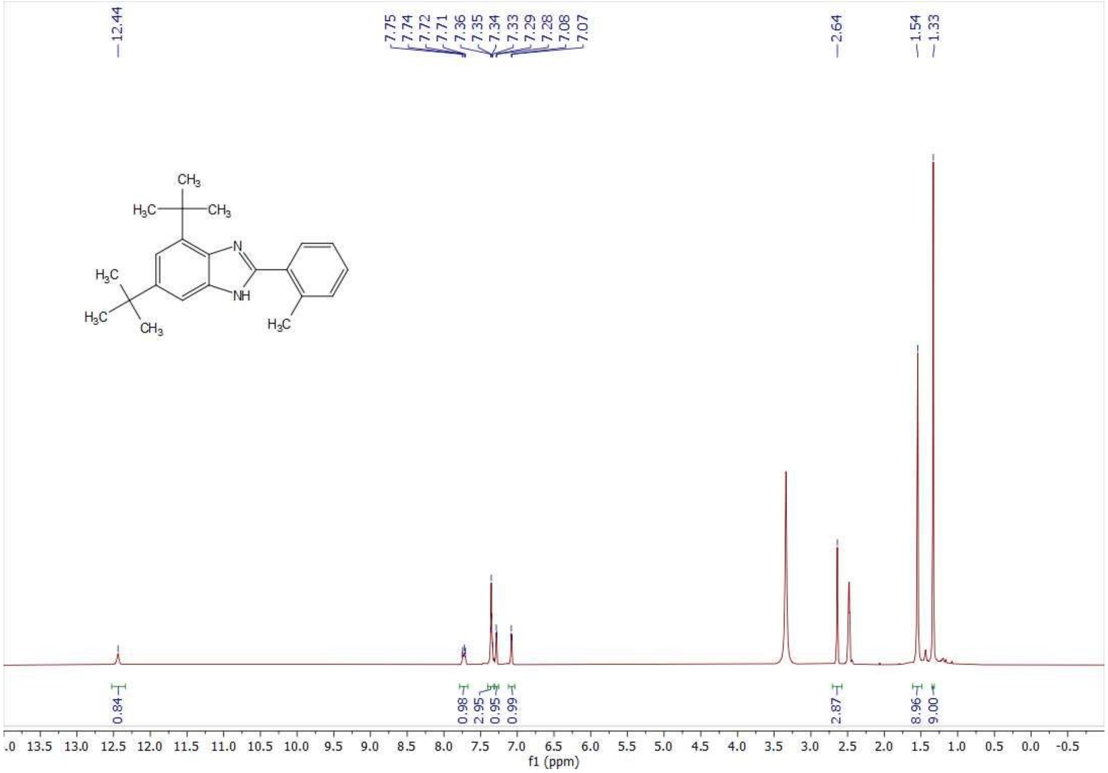

Figure S17. ^{1}**H NMR spectrum of compound 4i in DMSO-*d$_6$* (250 MHz).**

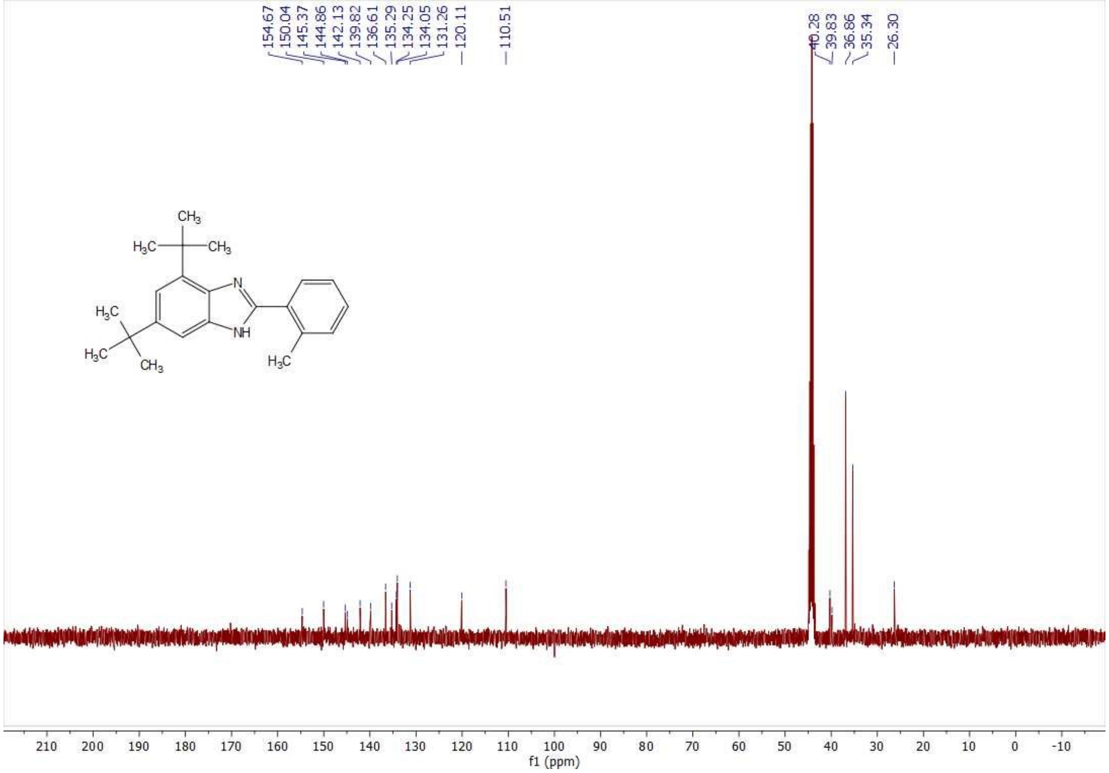

Figure S18. ^{13}**C NMR spectrum of compound 4i in DMSO-*d$_6$* (100 MHz).**

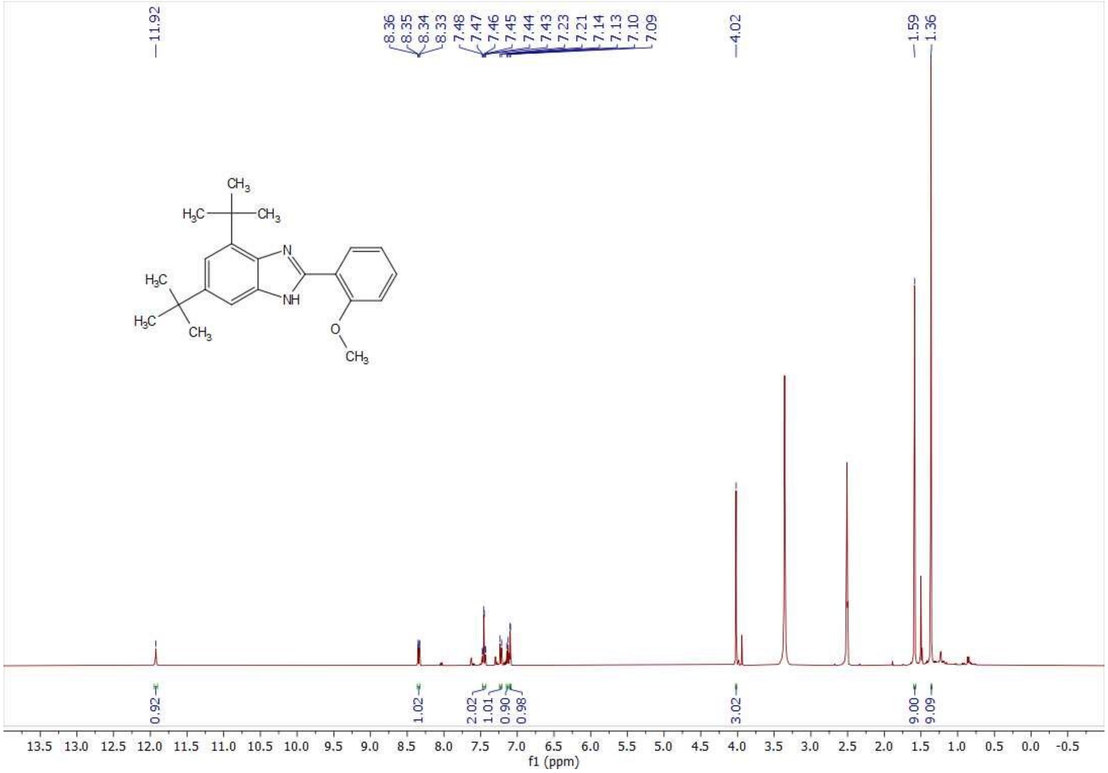

Figure S19. ^{1}H NMR spectrum of compound 4j in DMSO-d_6 (400 MHz).

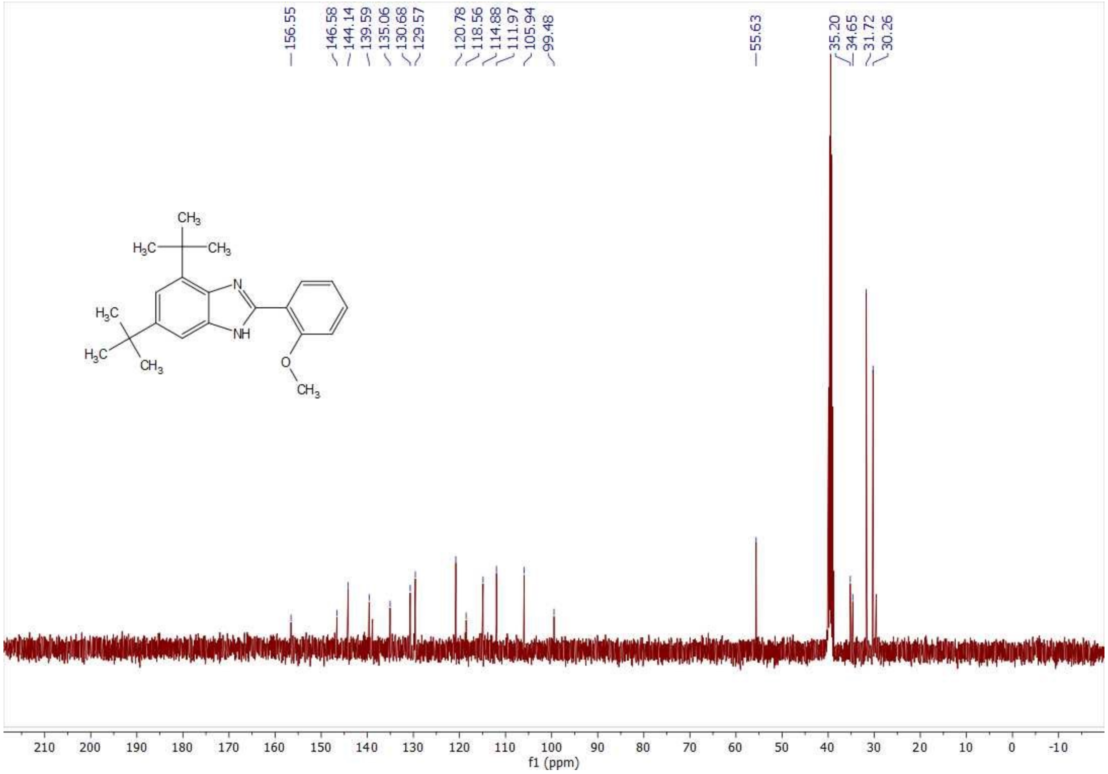

Figure S20. ^{13}C NMR spectrum of compound 4j in DMSO-d_6 (100 MHz).

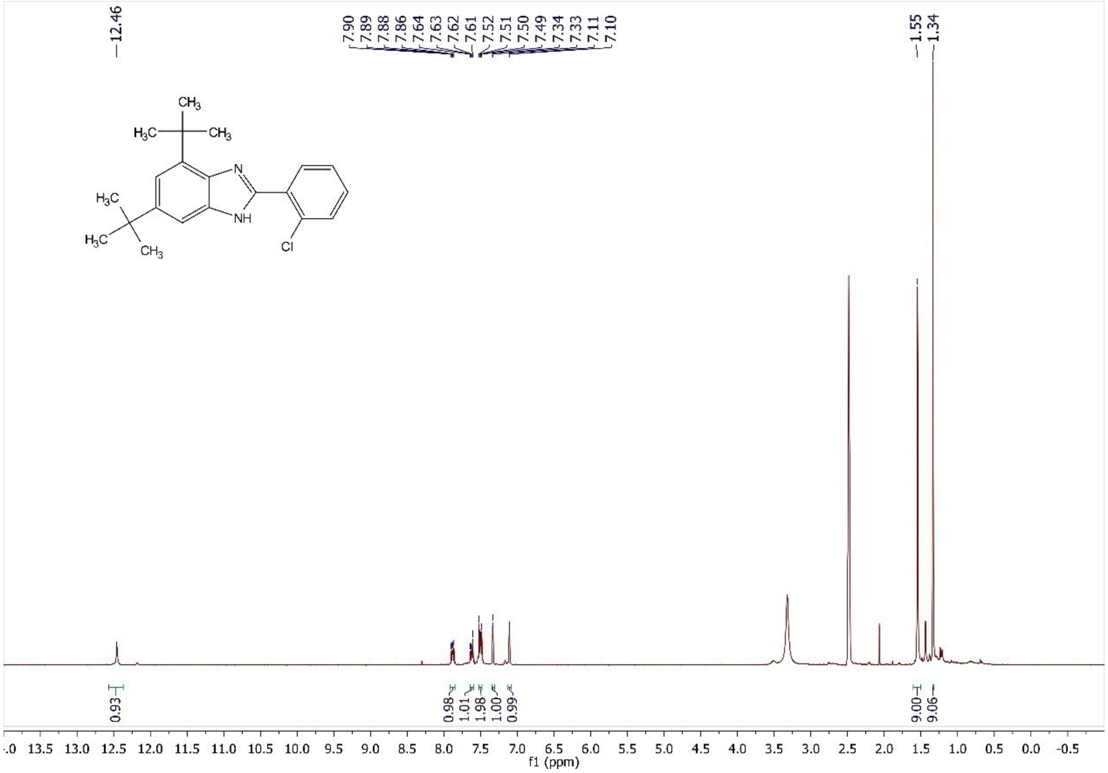

Figure S21. ^{1}**H NMR spectrum of compound 4k in DMSO-*d***$_6$ **(250 MHz).**

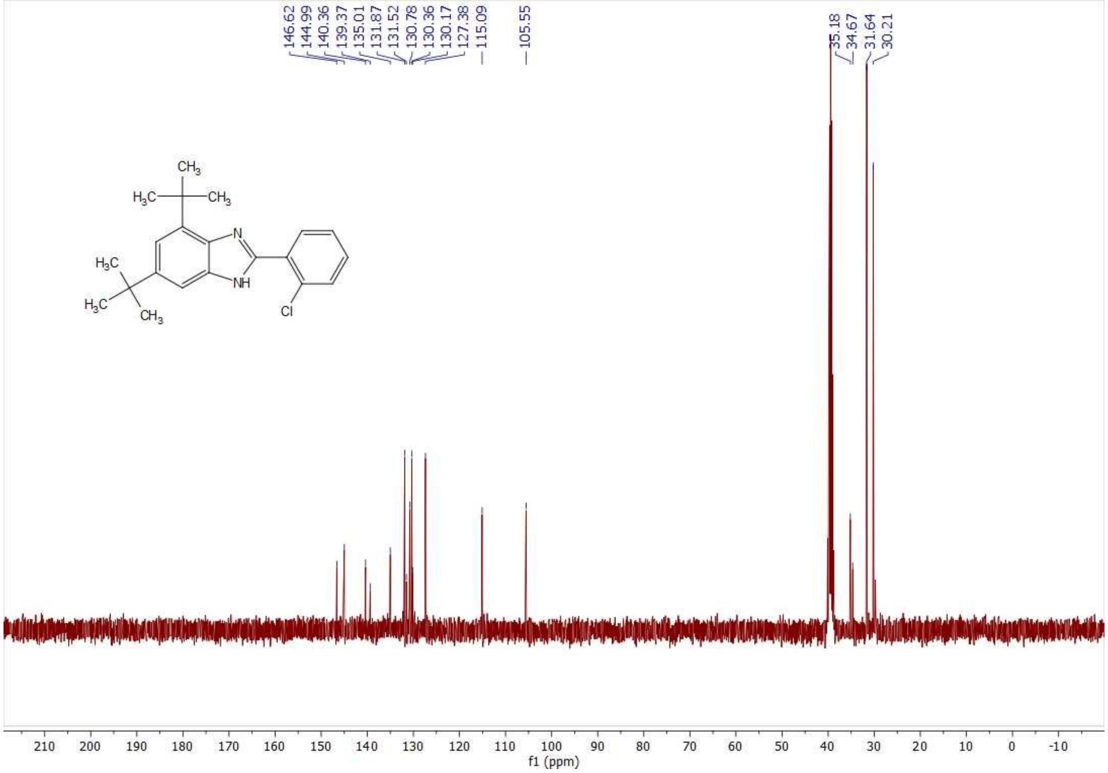

Figure S22. ^{13}**C NMR spectrum of compound 4k in DMSO-*d***$_6$ **(100 MHz).**

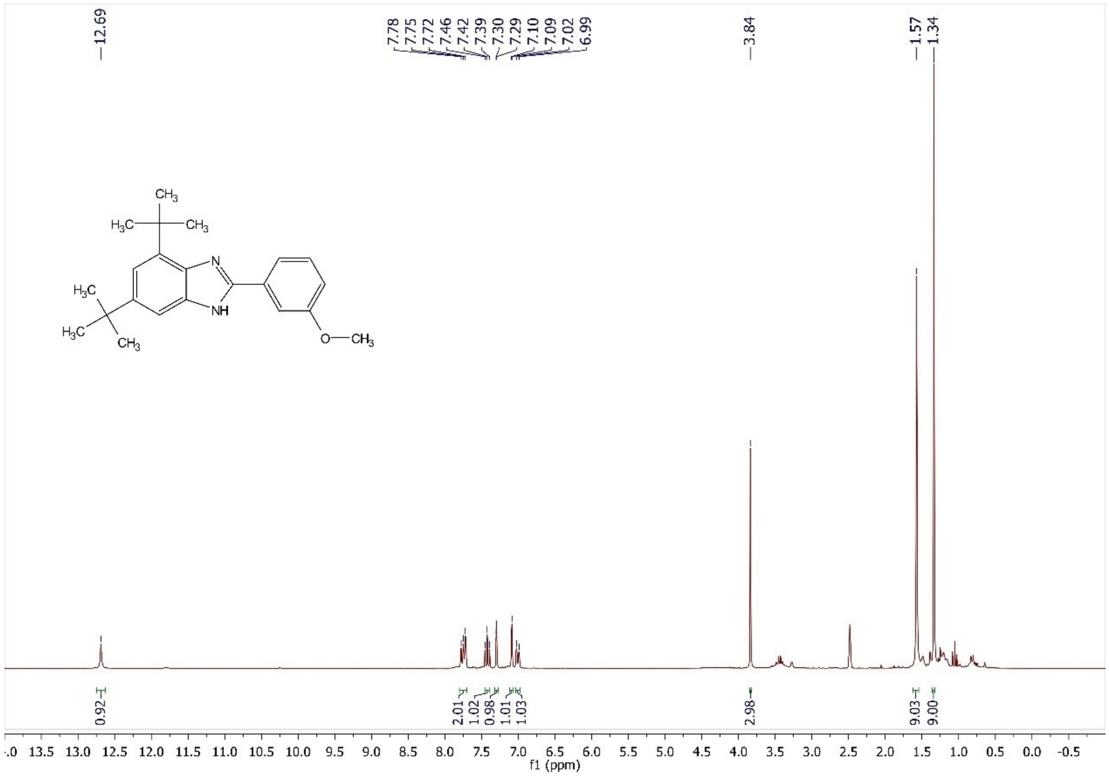

Figure S23. ¹H NMR spectrum of compound 4l in DMSO-d_6 (250 MHz).

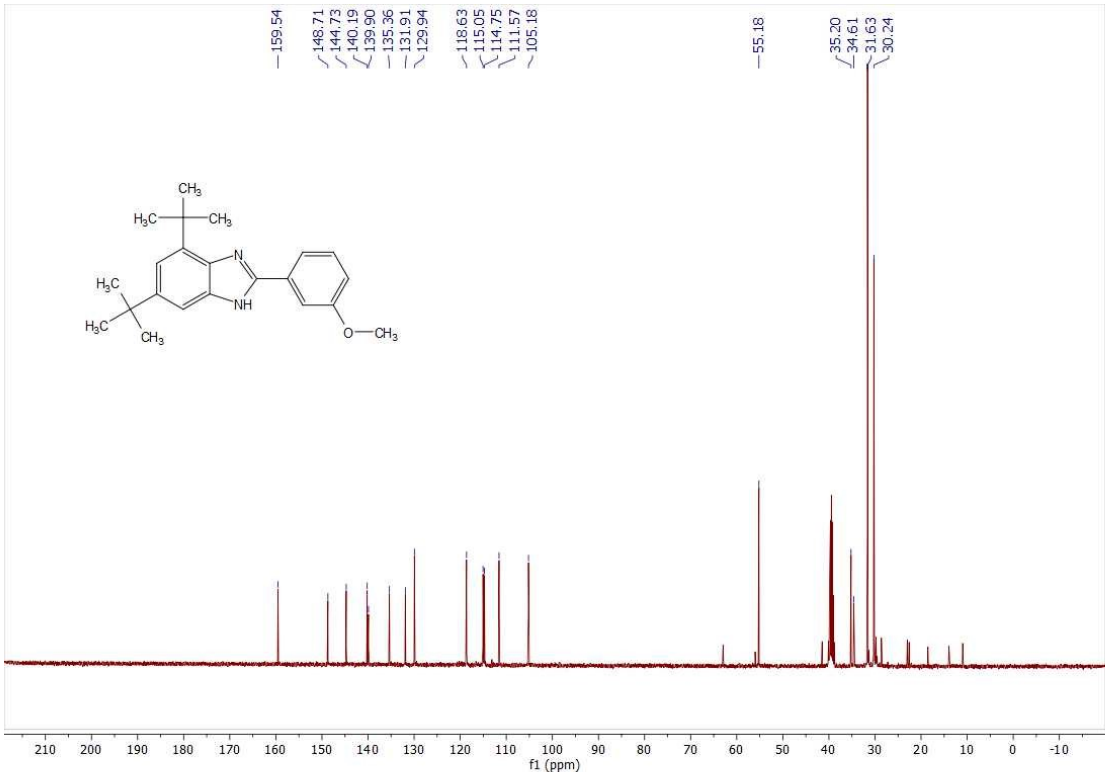

Figure S24. ¹³C NMR spectrum of compound 4l in DMSO-d_6 (100 MHz).

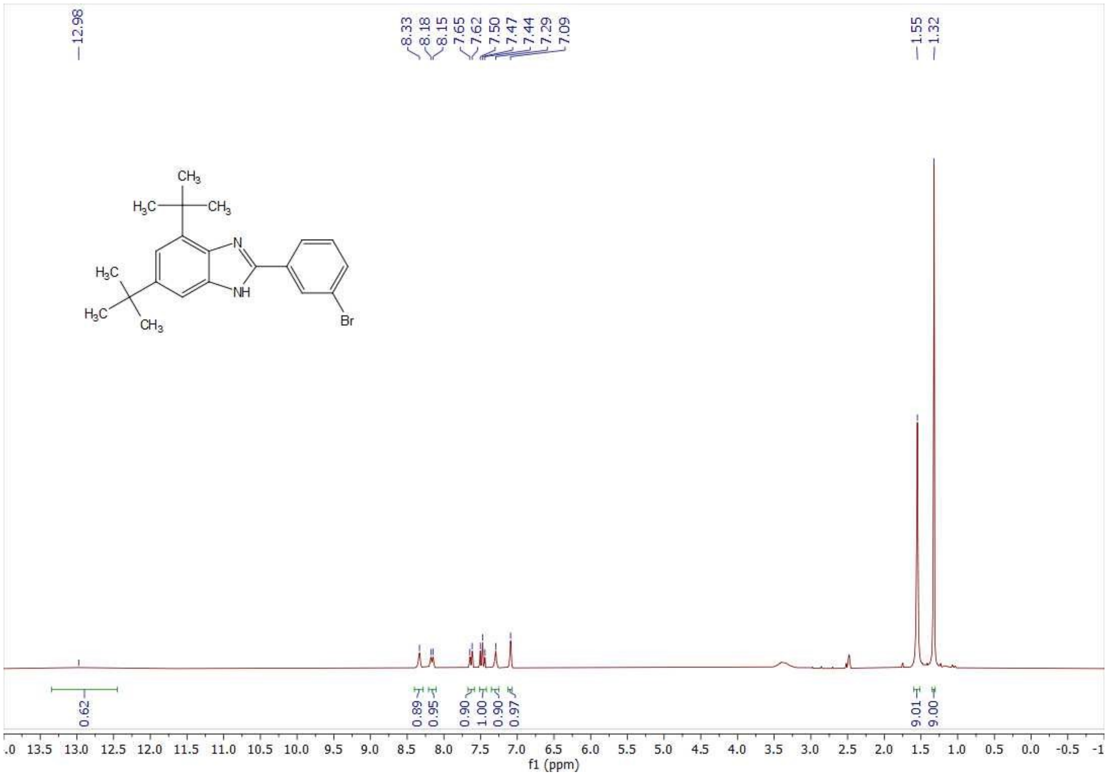

Figure S25. ^{1}H NMR spectrum of compound 4m in DMSO-d_6 (250 MHz).

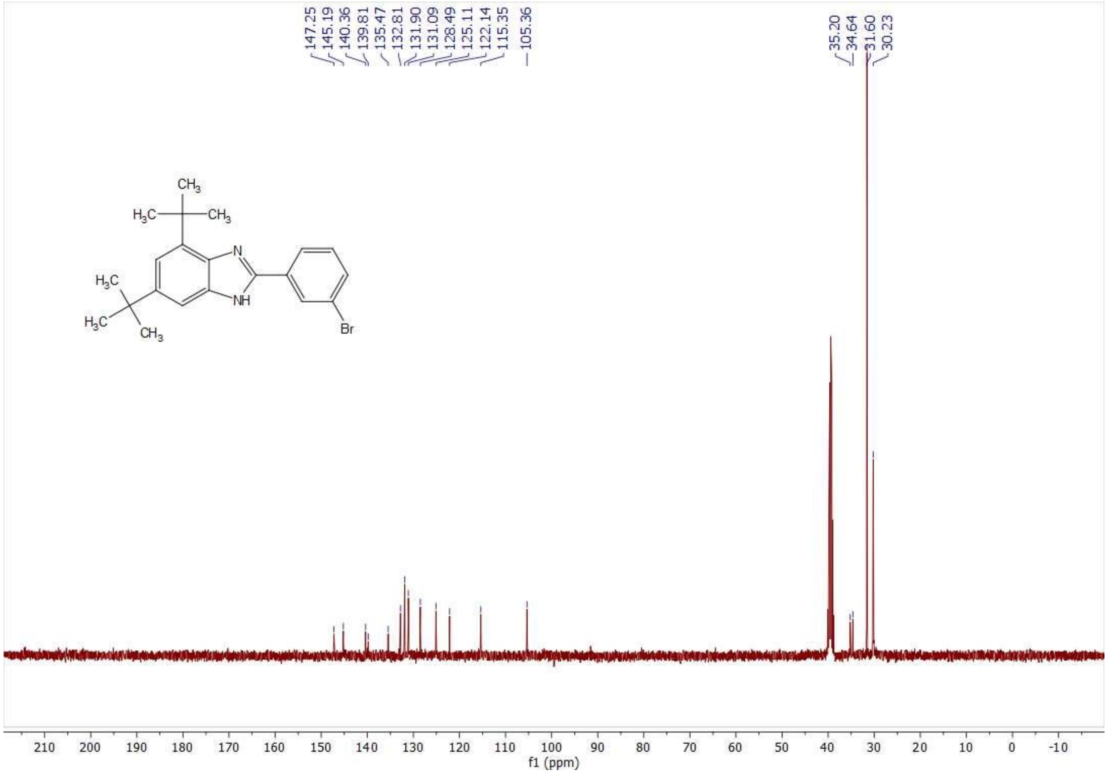

Figure S26. ^{13}C NMR spectrum of compound 4m in DMSO-d_6 (100 MHz).

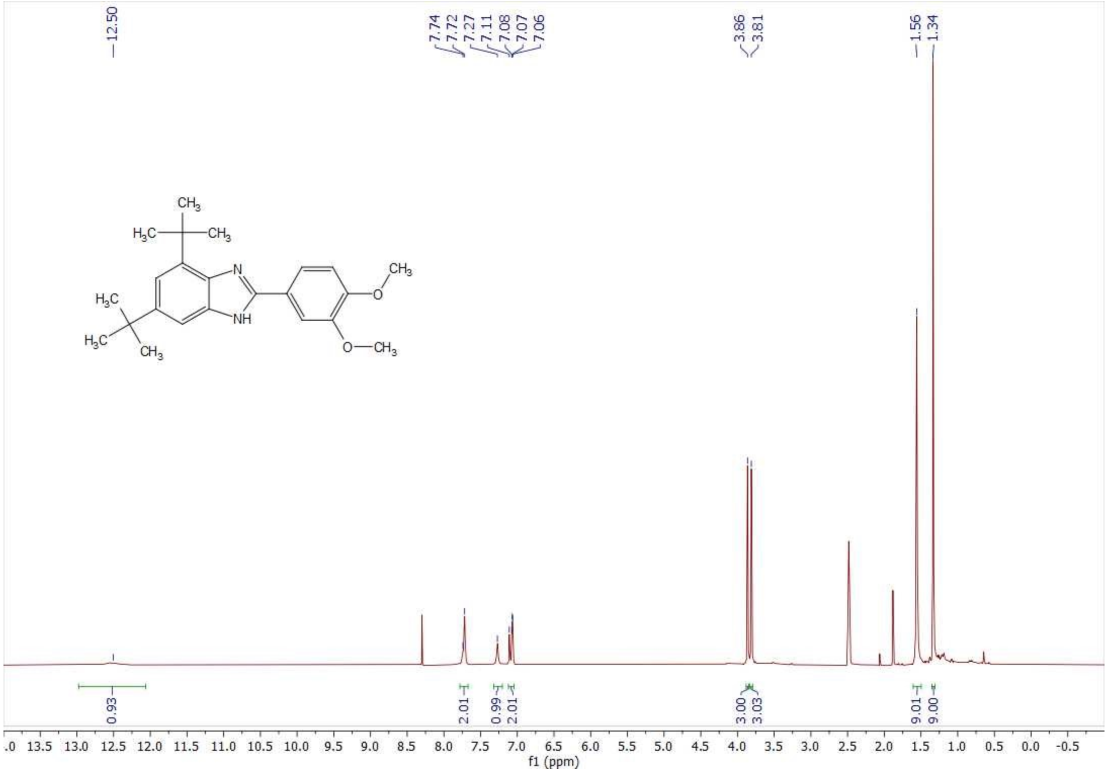

Figure S27. **^{1}H NMR spectrum of compound 4n in DMSO-d_6 (250 MHz).**

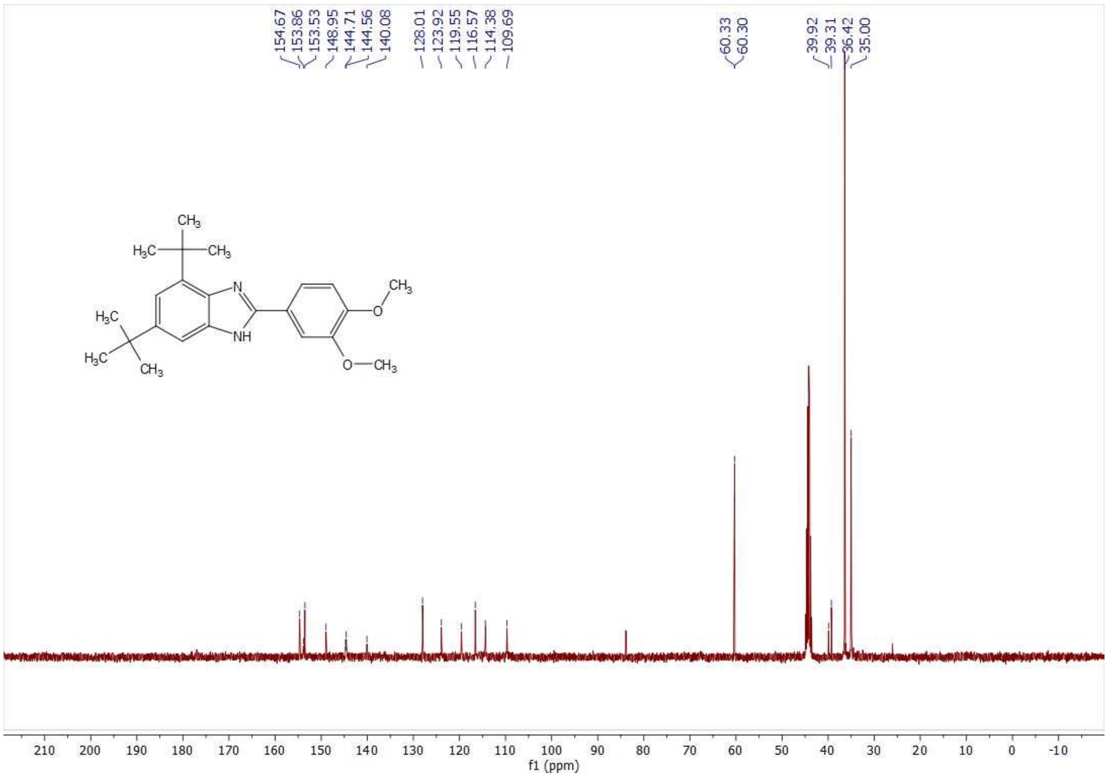

Figure S28. **^{13}C NMR spectrum of compound 4n in DMSO-d_6 (100 MHz).**

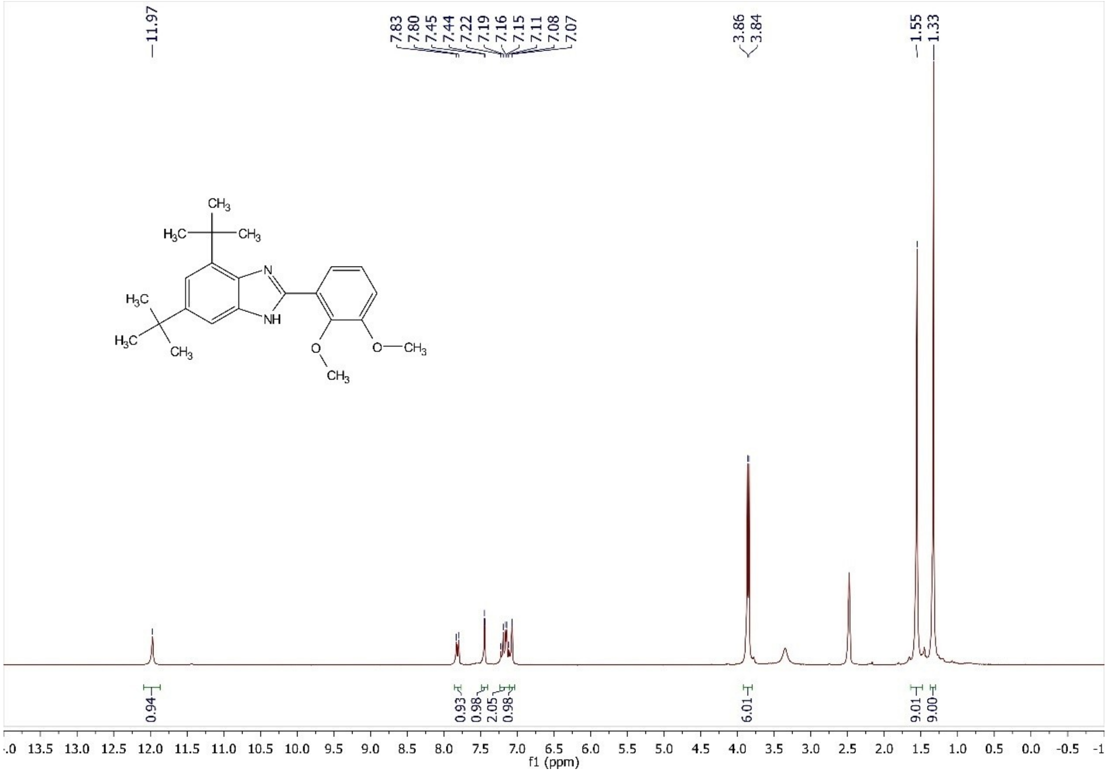

Figure S29. ^{1}H NMR spectrum of compound 4o in DMSO-*d₆* (250 MHz).

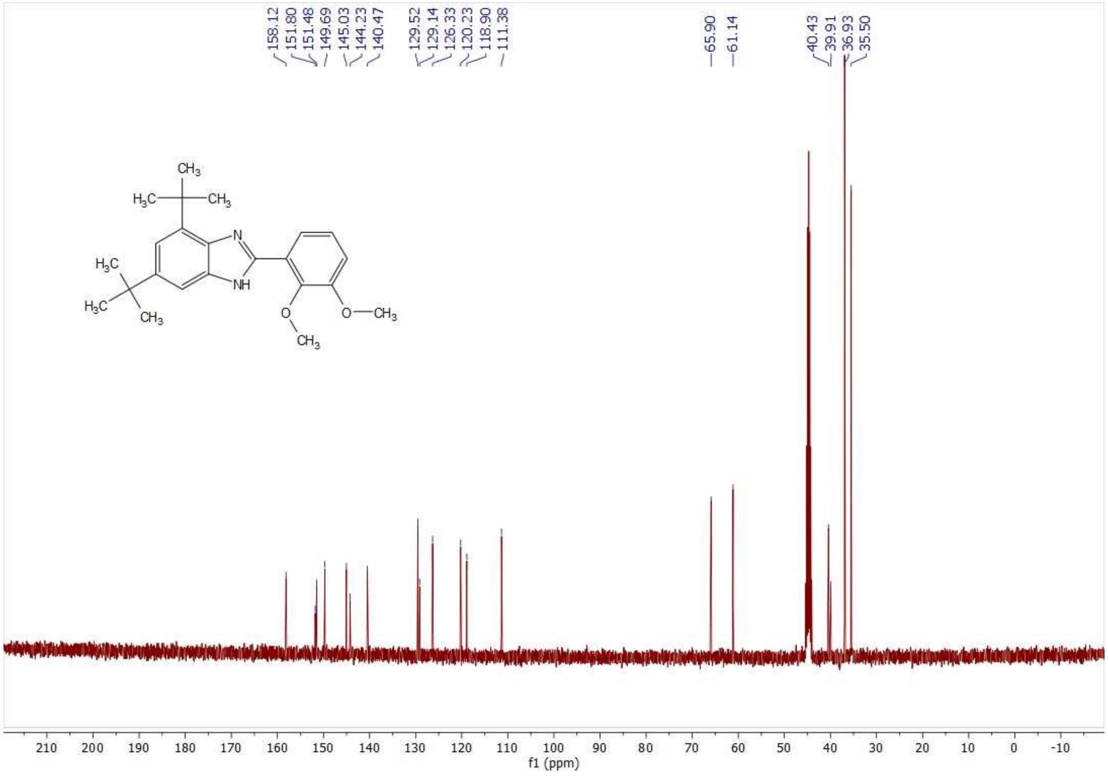

Figure S30. ^{13}C NMR spectrum of compound 4o in DMSO-*d₆* (100 MHz).

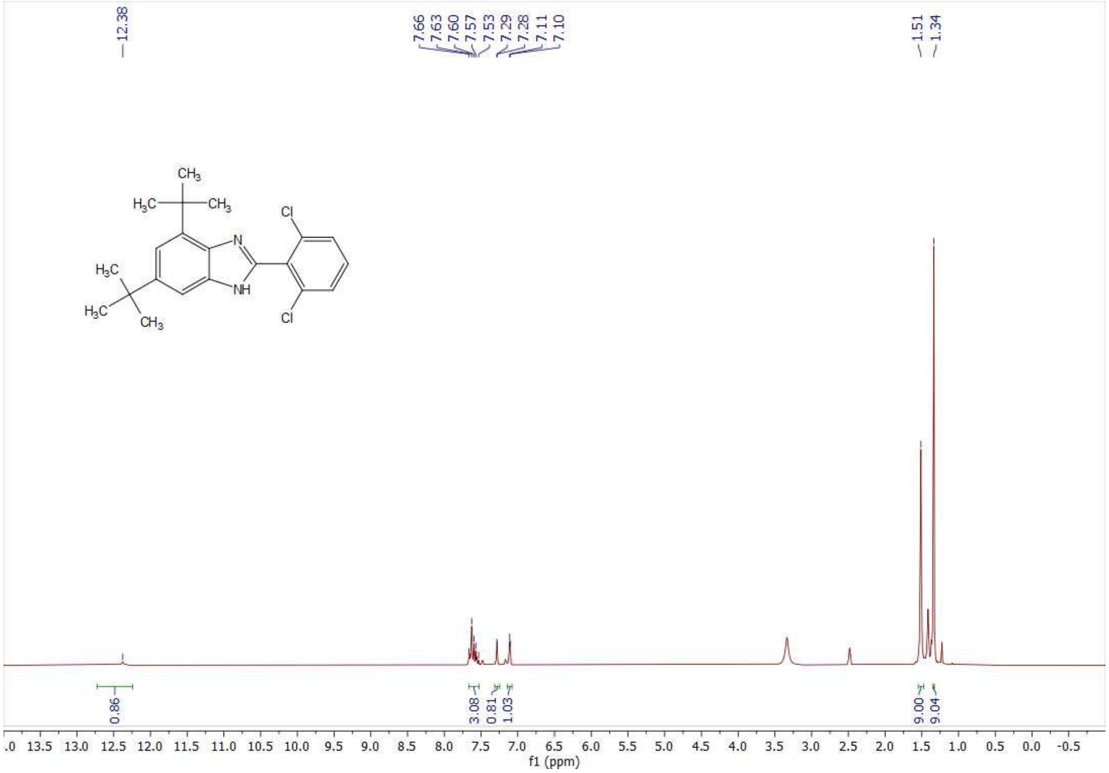

Figure S31. ¹H NMR spectrum of compound 4p in DMSO-*d₆* (250 MHz).

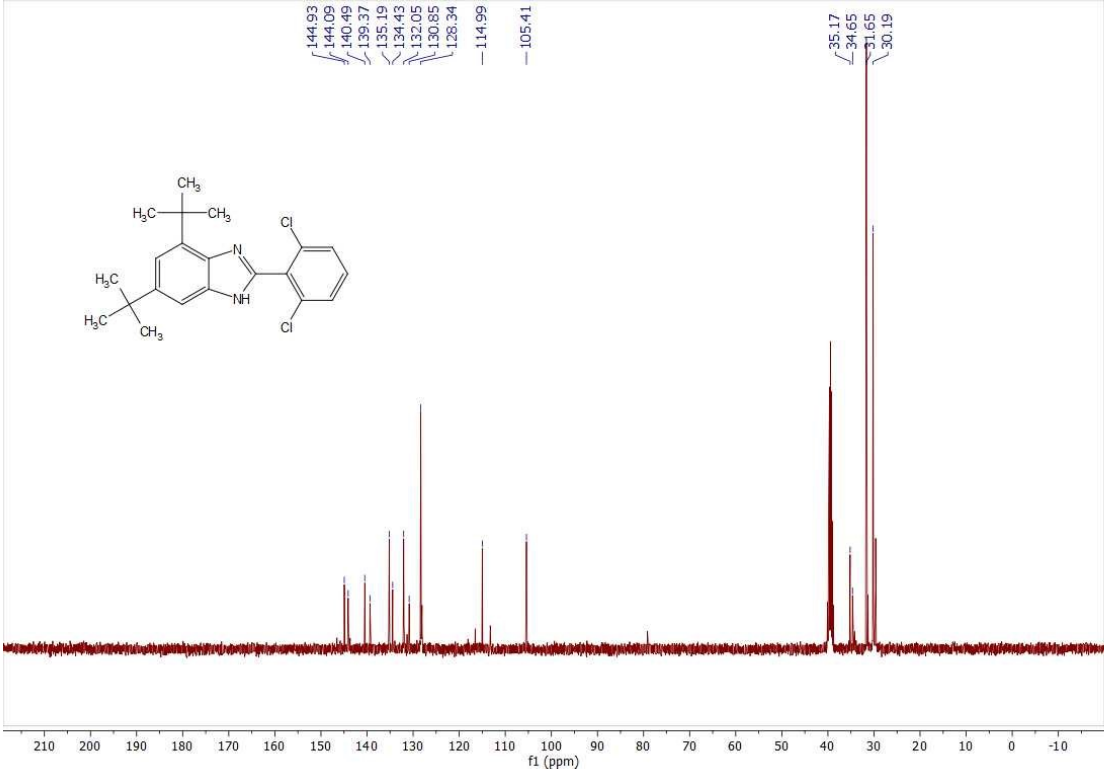

Figure S32. ¹³C NMR spectrum of compound 4p in DMSO-*d₆* (100 MHz).

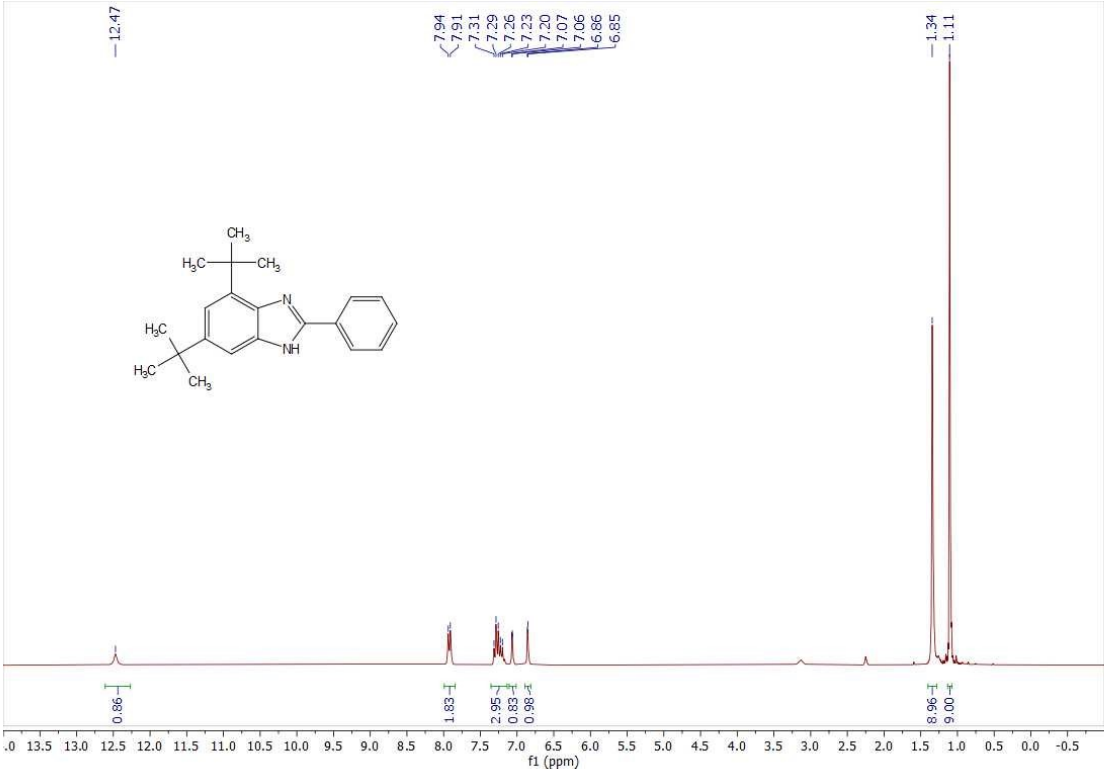

Figure S33. ^{1}H NMR spectrum of compound 4q in DMSO-d_6 (250 MHz).

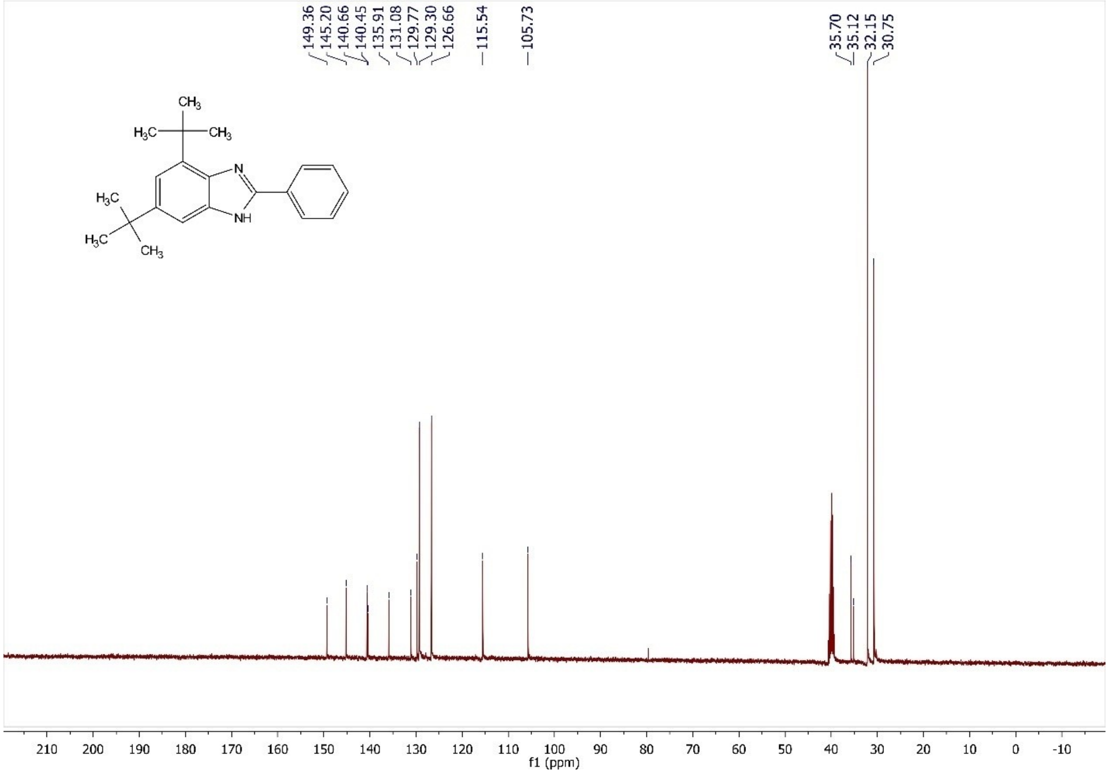

Figure S34. ^{13}C NMR spectrum of compound 4q in DMSO-d_6 (100 MHz).

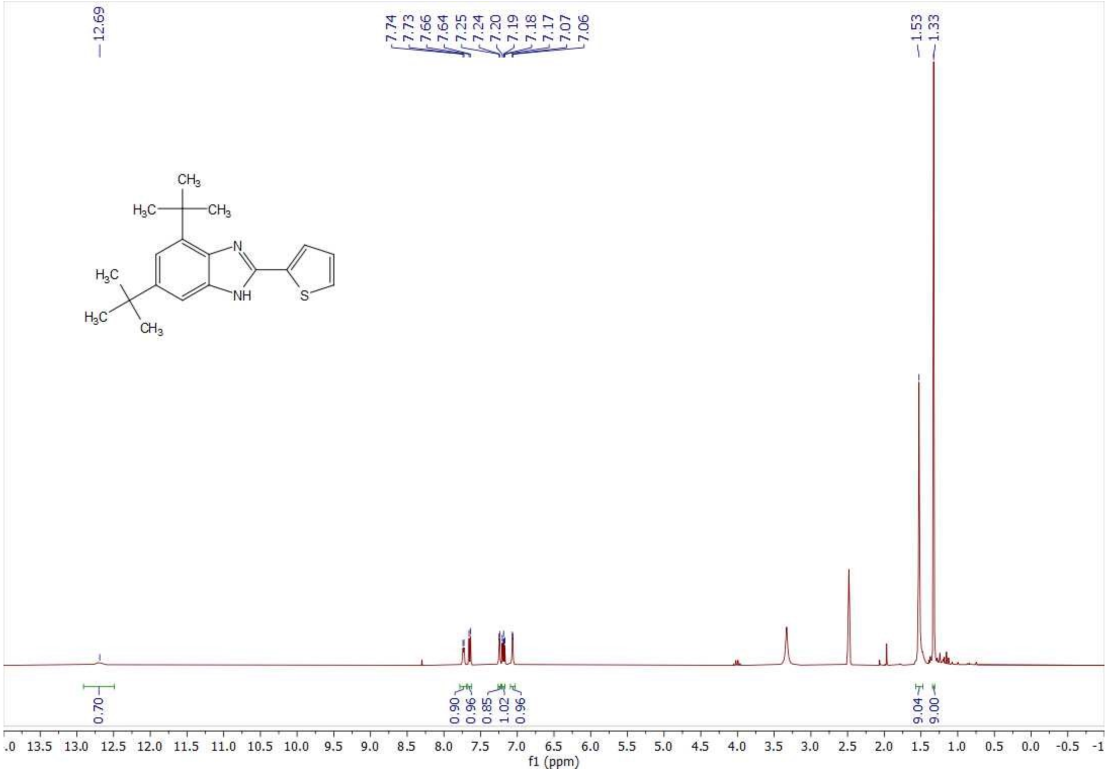

Figure S35. ^{1}H NMR spectrum of compound 4r in DMSO-d_6 (250 MHz).

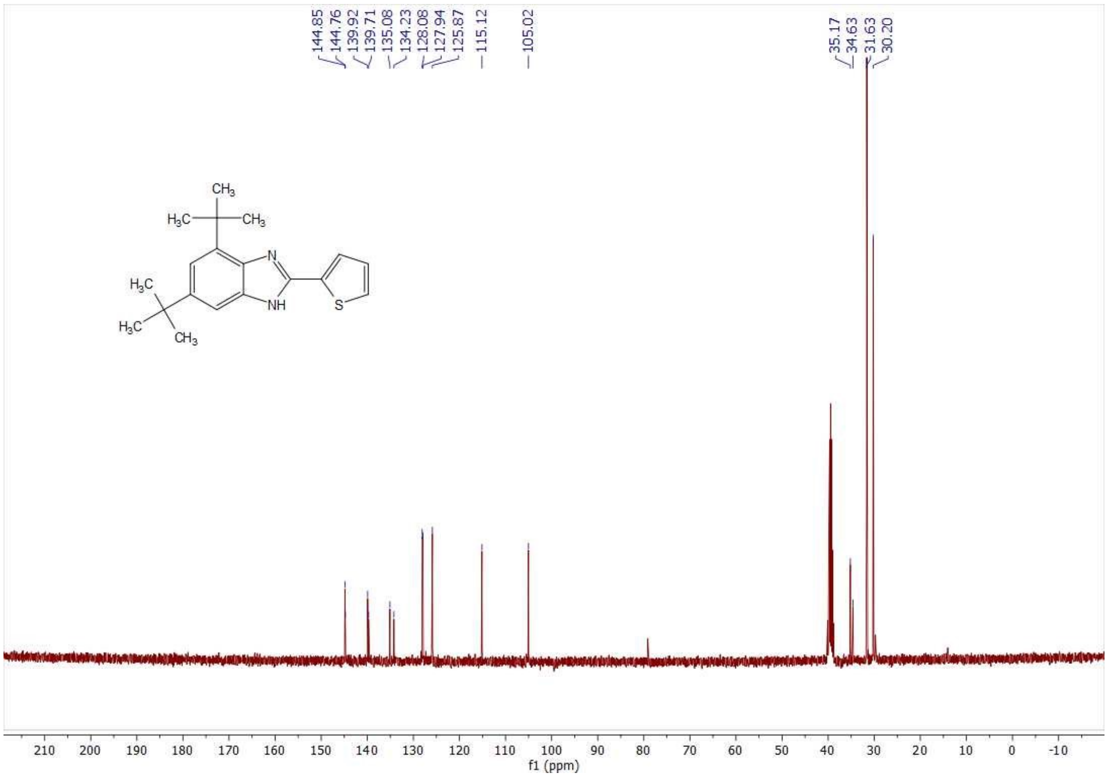

Figure S36. ^{13}C NMR spectrum of compound 4r in DMSO-d_6 (100 MHz).

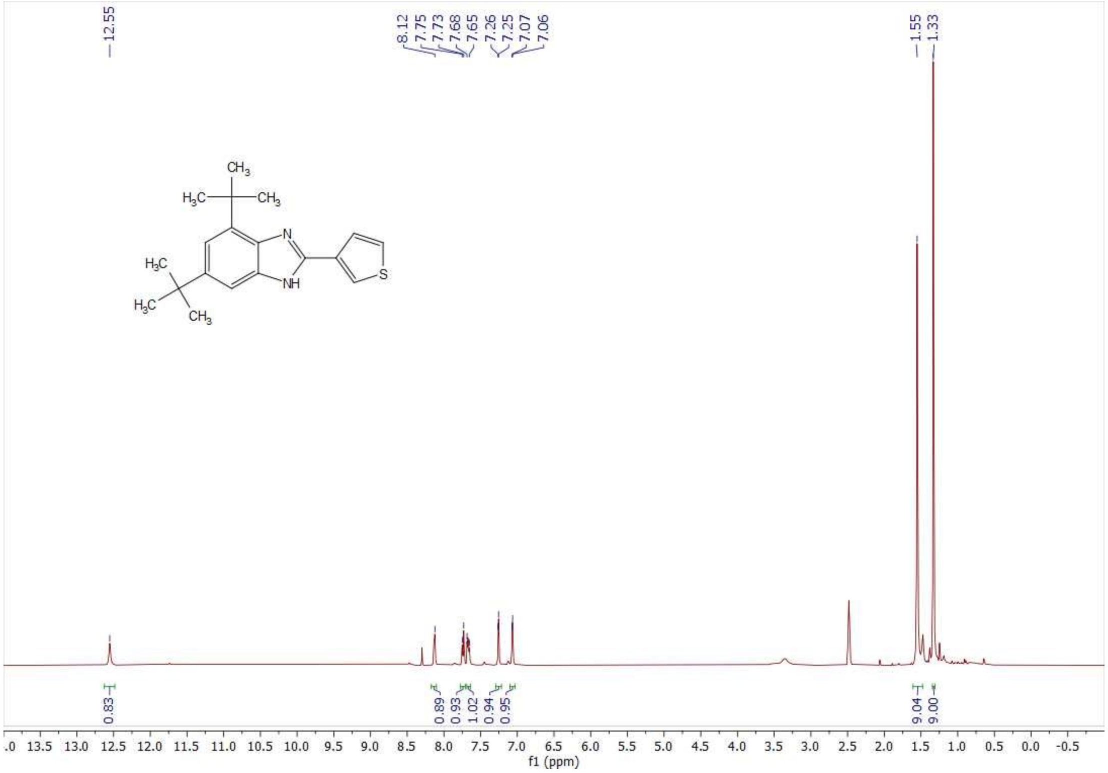

Figure S37. ^{1}H NMR spectrum of compound 4s in DMSO-d_6 (250 MHz).

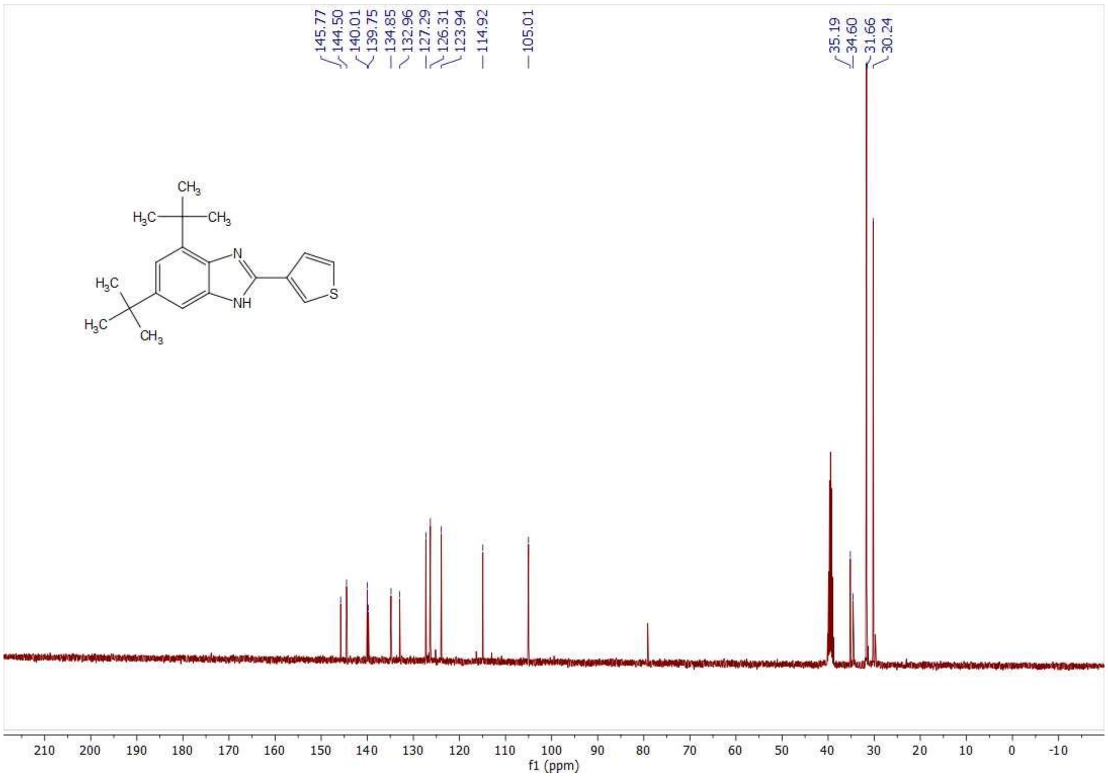

Figure S38. ^{13}C NMR spectrum of compound 4s in DMSO-d_6 (100 MHz).

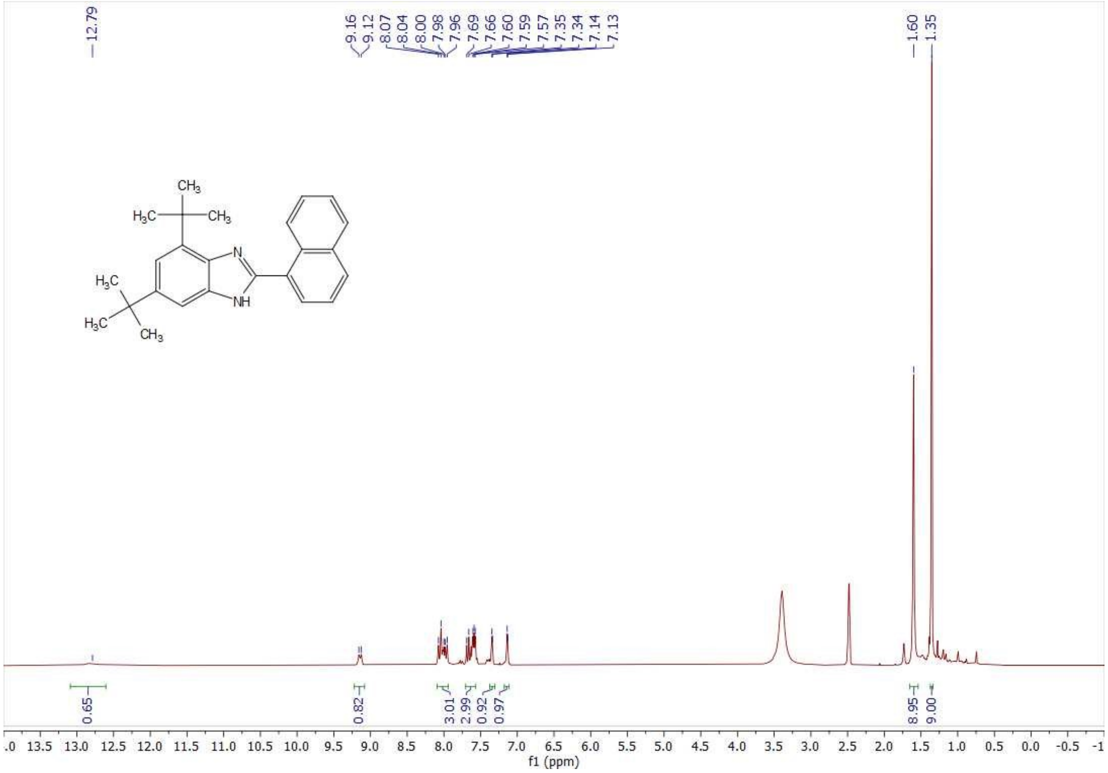

Figure S39. ^{1}H **NMR spectrum of compound 4t in DMSO-d_6 (250 MHz).**

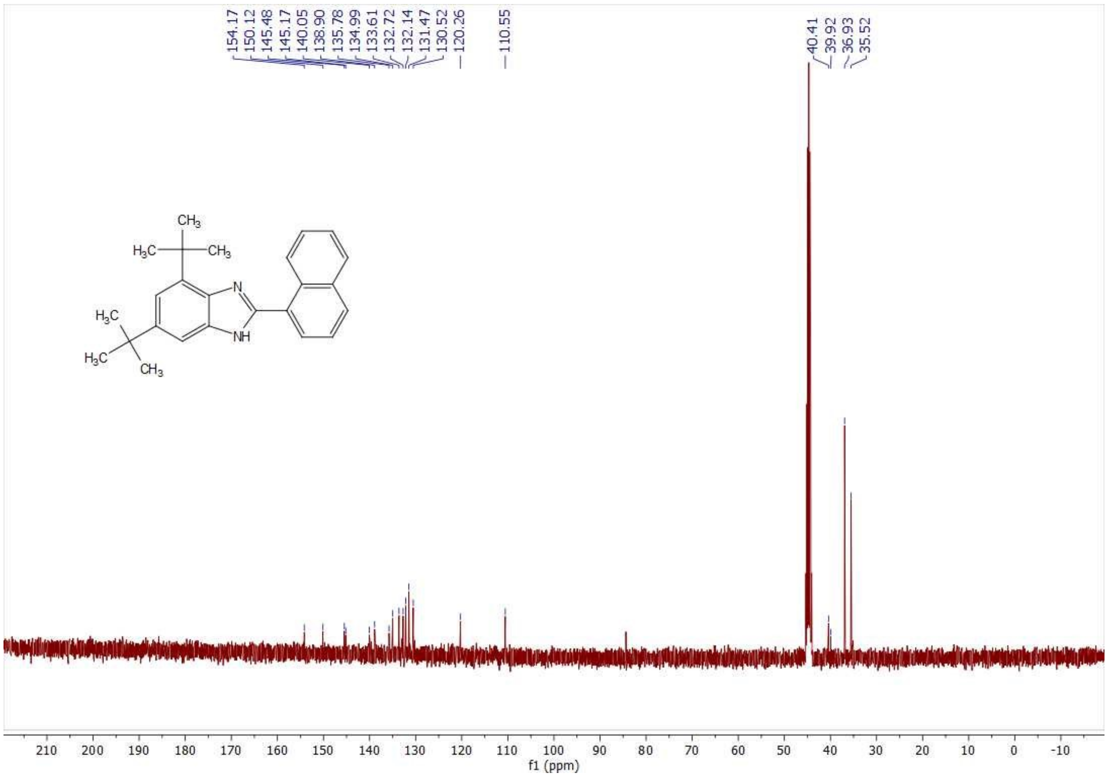

Figure S40. ^{13}C **NMR spectrum of compound 4t in DMSO-d_6 (100 MHz).**

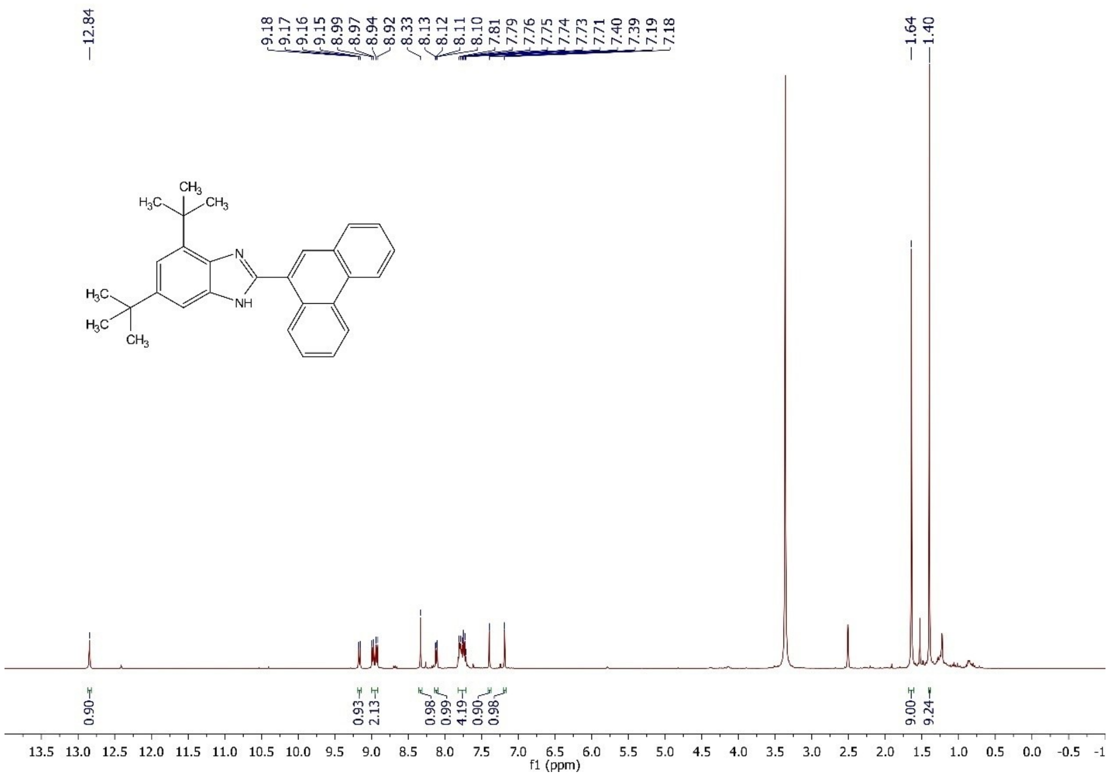

Figure S41. ^{1}H NMR spectrum of compound 4u in DMSO-d_6 (400MHz).

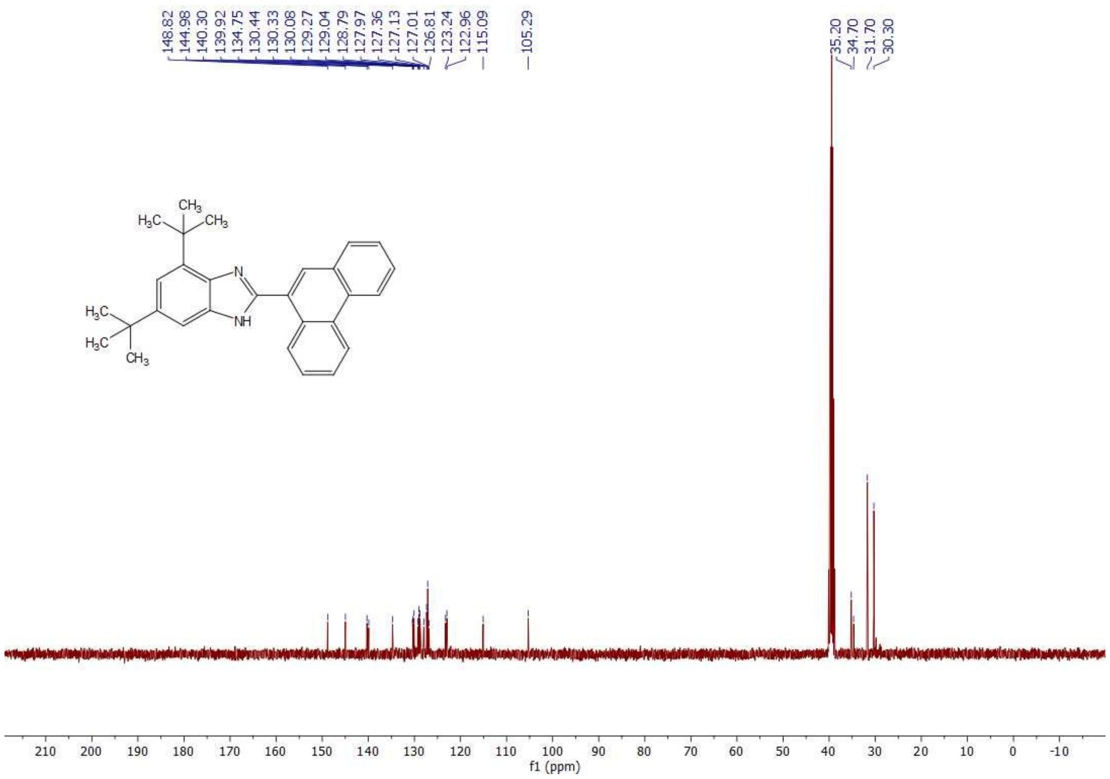

Figure S42. ^{13}C NMR spectrum of compound 4u in DMSO-d_6 (100 MHz).

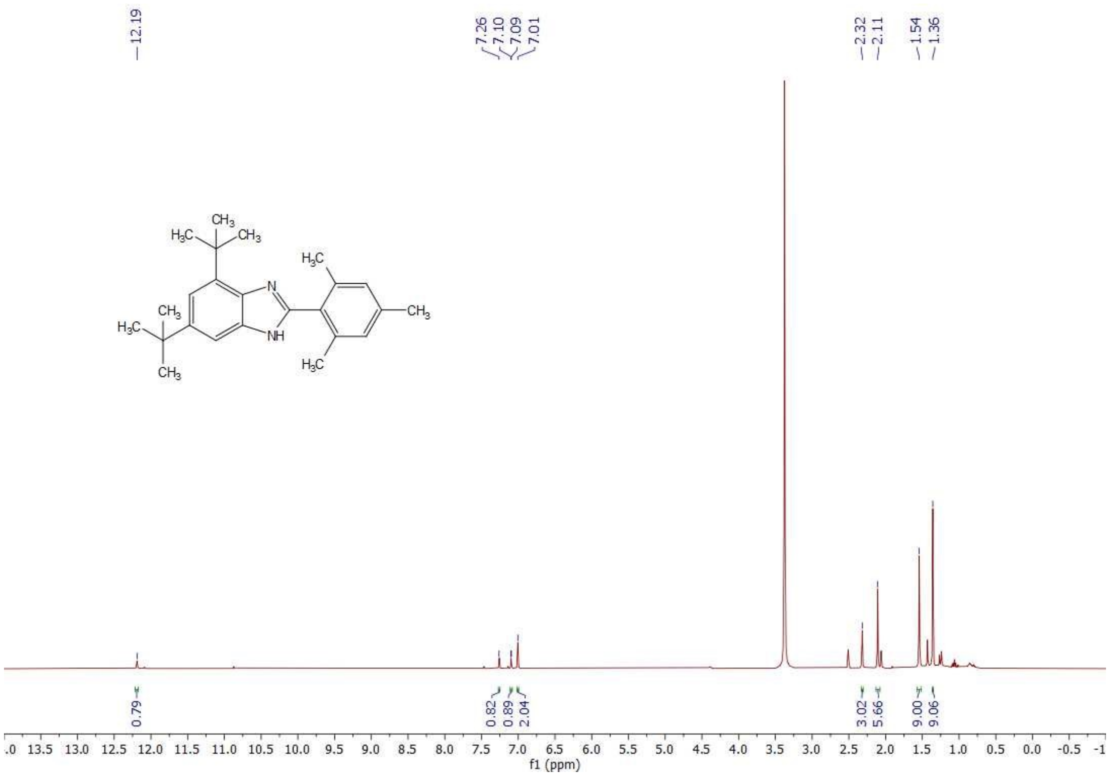

Figure S43. ^{1}H NMR spectrum of compound 4vin DMSO-d_6 (400MHz).

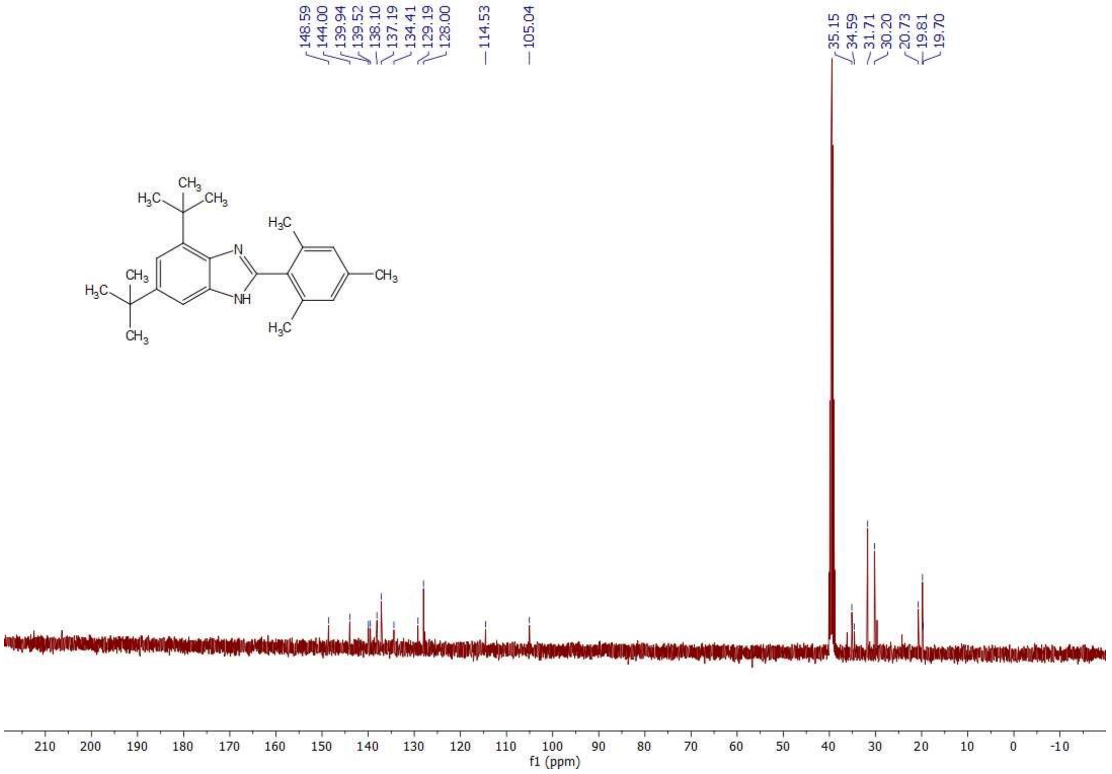

Figure S44. ^{13}C NMR spectrum of compound 4v in DMSO-d_6 (100 MHz).

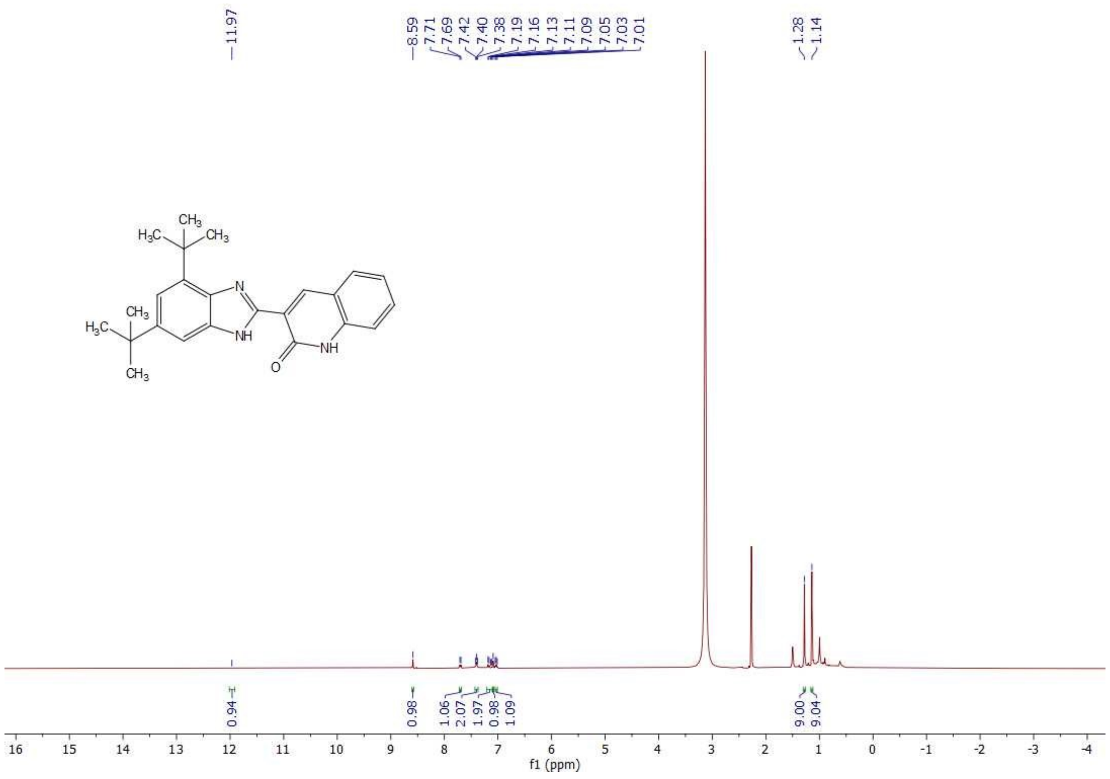

Figure S45. ^{1}H NMR spectrum of compound 4w in DMSO-d_6 (400MHz).

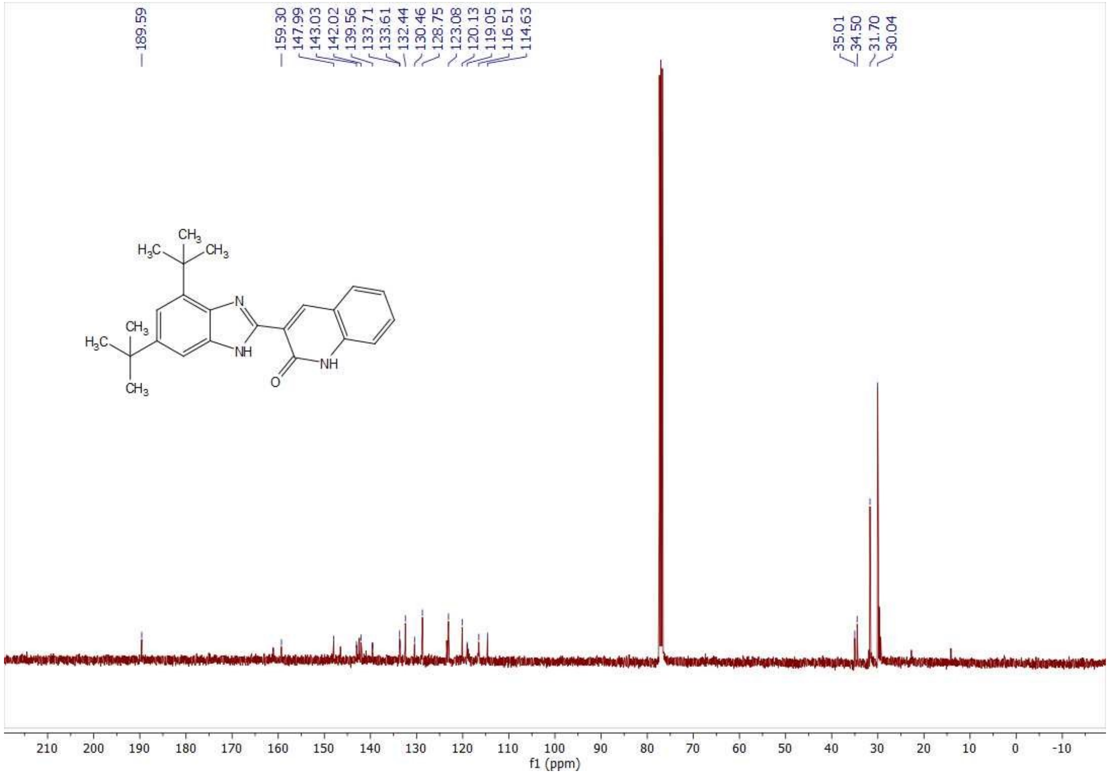

Figure S46. ^{13}C NMR spectrum of compound 4w in $CDCl_3$(100 MHz).

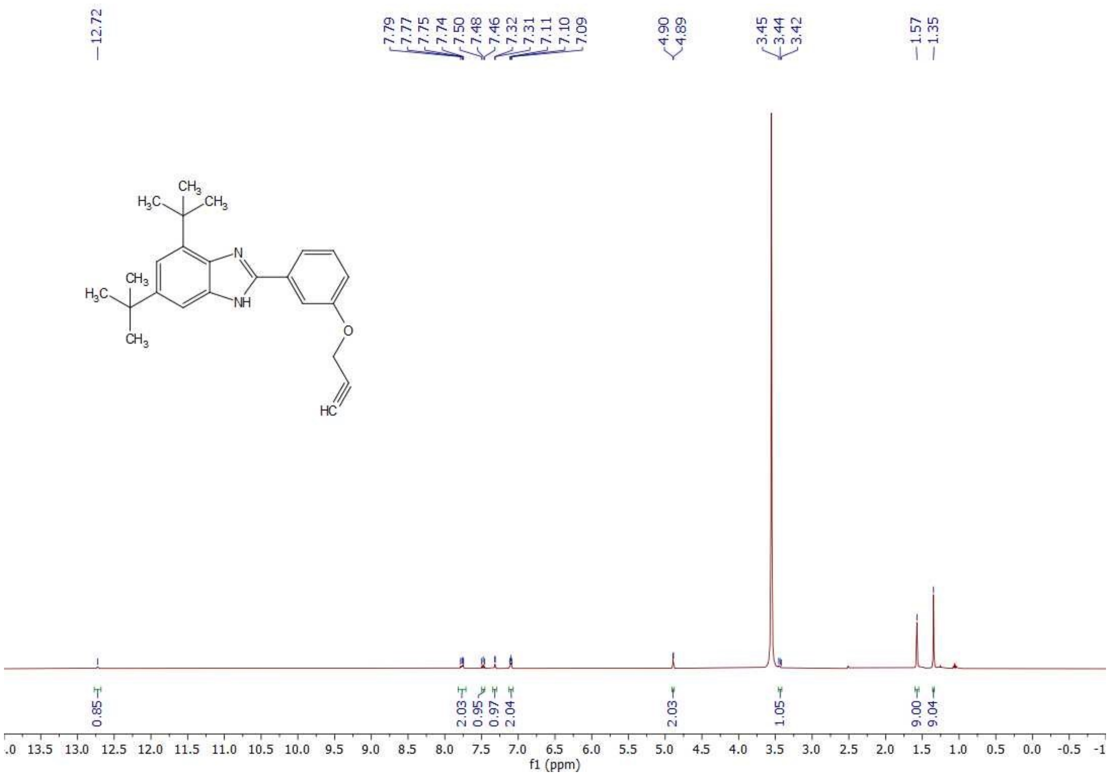

Figure S47. ^{1}**H NMR spectrum of compound 4x in DMSO-d_6 (400MHz).**

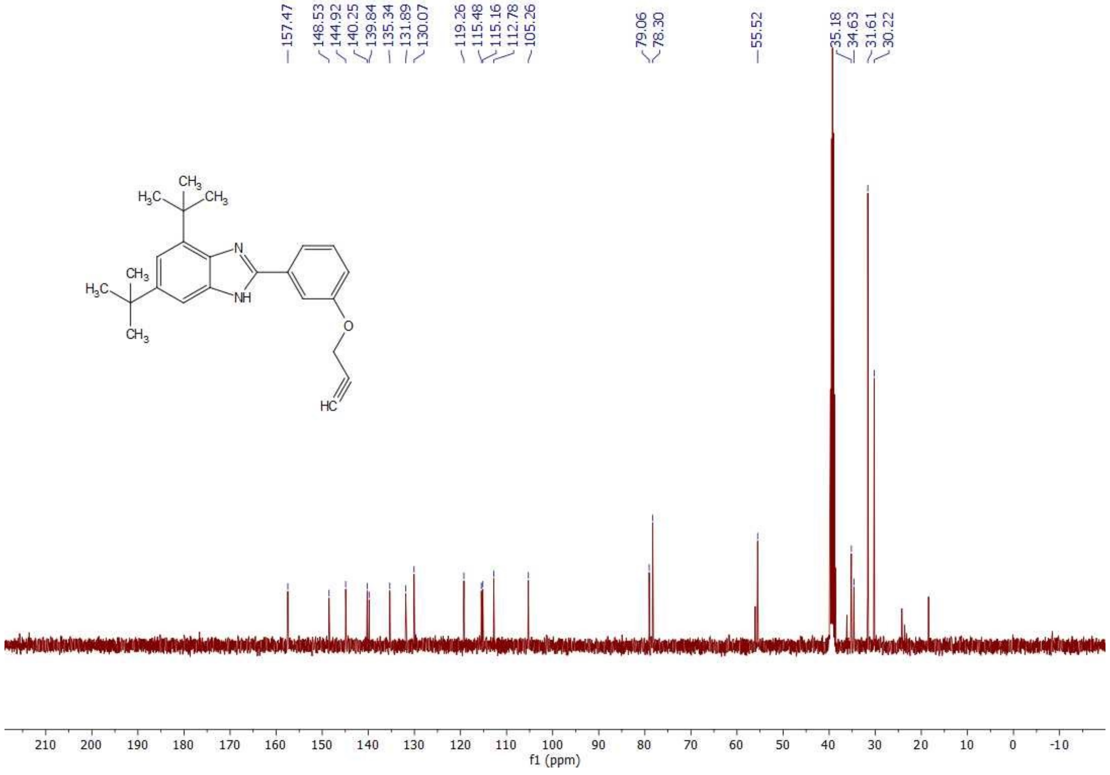

Figure S48. ^{13}**C NMR spectrum of compound 4x in DMSO-d_6 (100 MHz).**

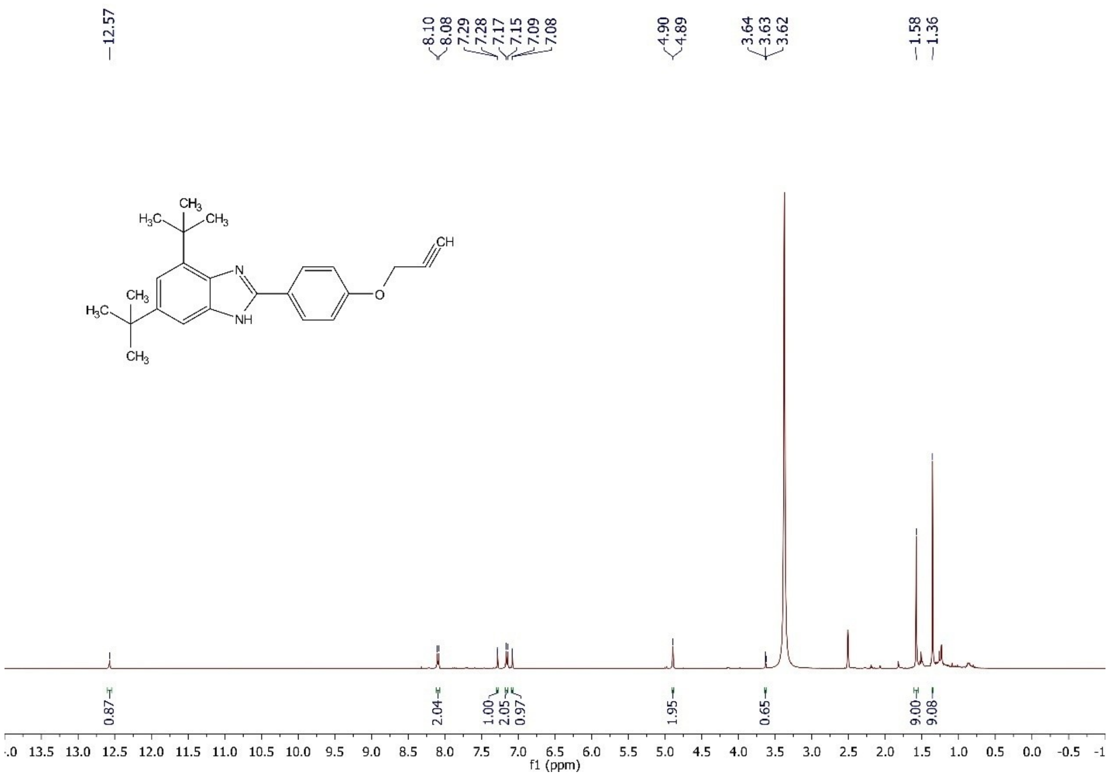

Figure S49. ^{1}H NMR spectrum of compound 4y in DMSO-d_6 (400MHz).

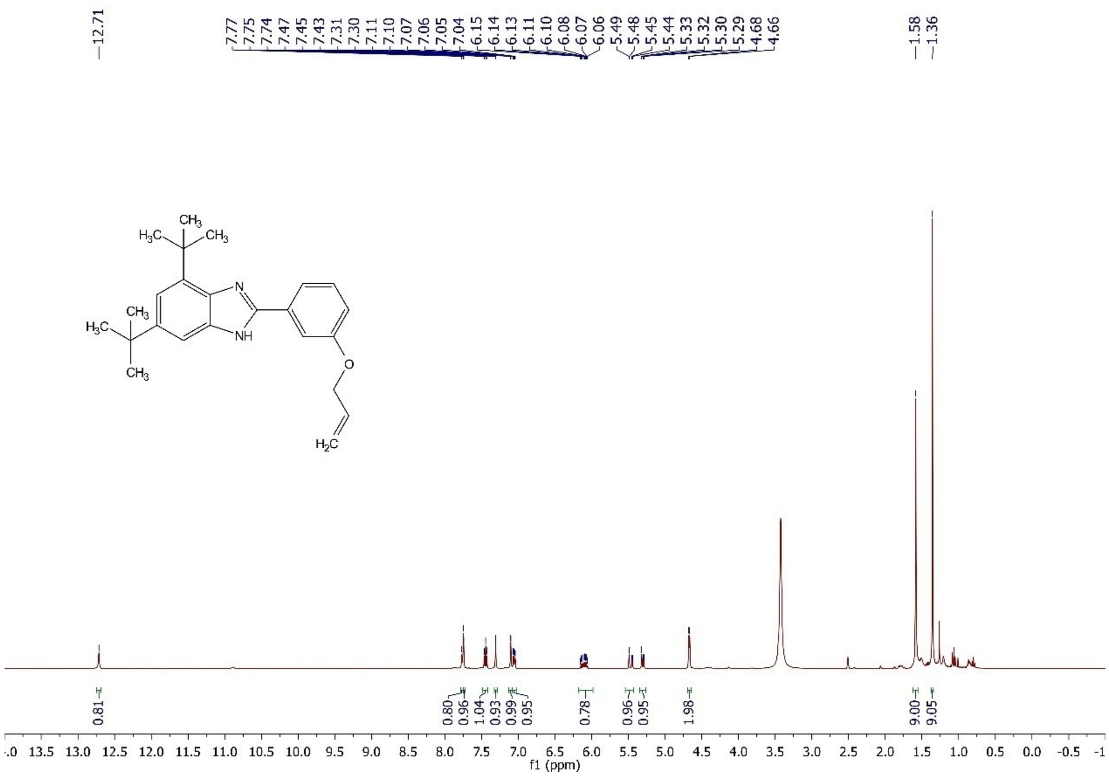

Figure S50. ^{1}H NMR spectrum of compound 4z in DMSO-d_6 (400MHz).

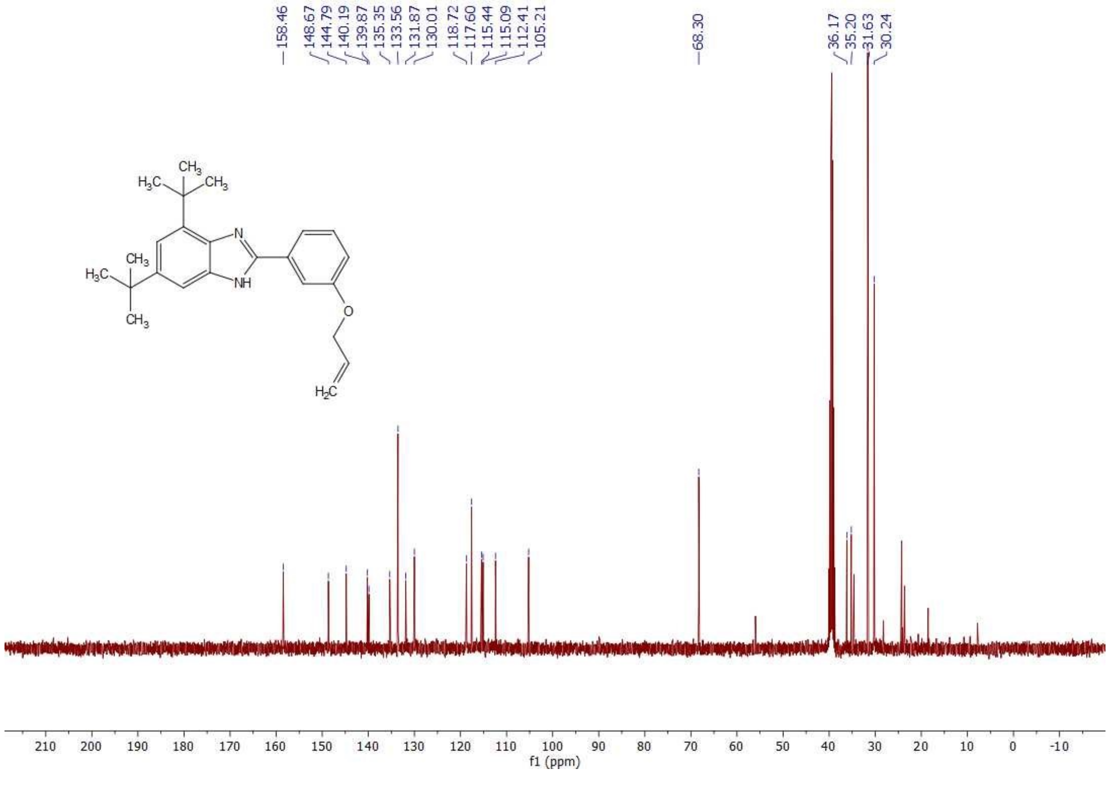

Figure S51. ^{13}C NMR spectrum of compound 4z in DMSO-d_6 (100 MHz).

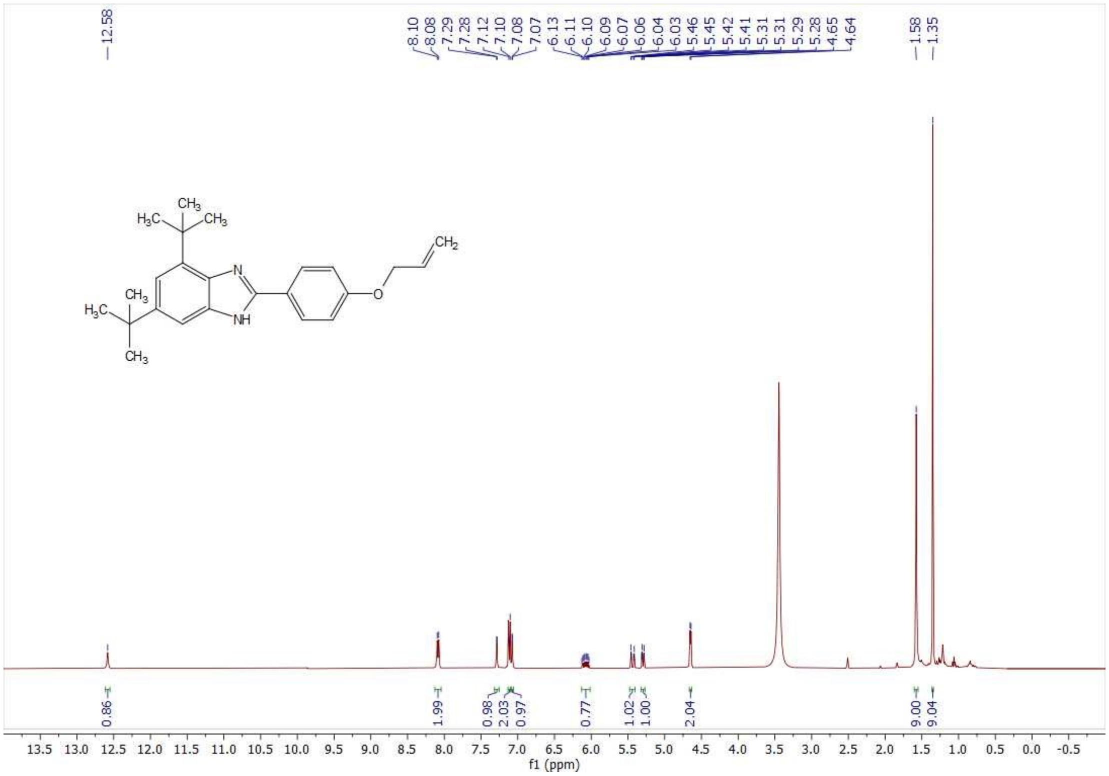

Figure S52. ^{1}H NMR spectrum of compound 4aa in DMSO-d_6 (400 MHz).

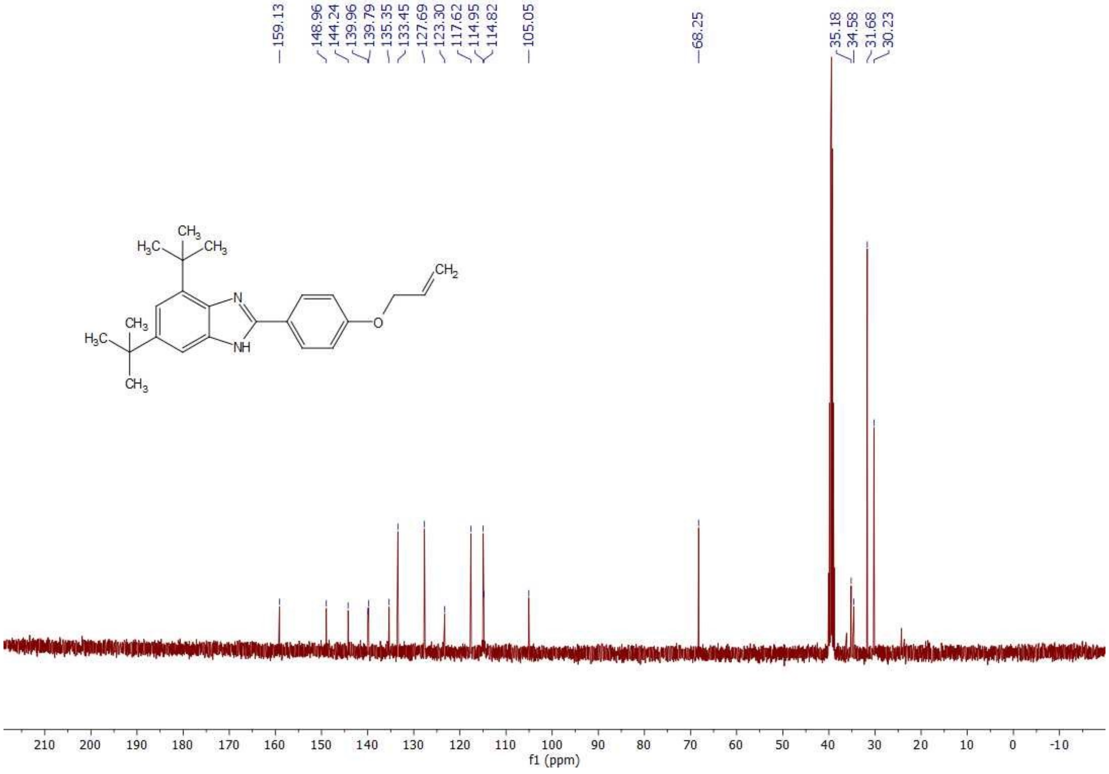

Figure S53. ^{13}C NMR spectrum of compound 4aa in DMSO-d_6 (100 MHz).

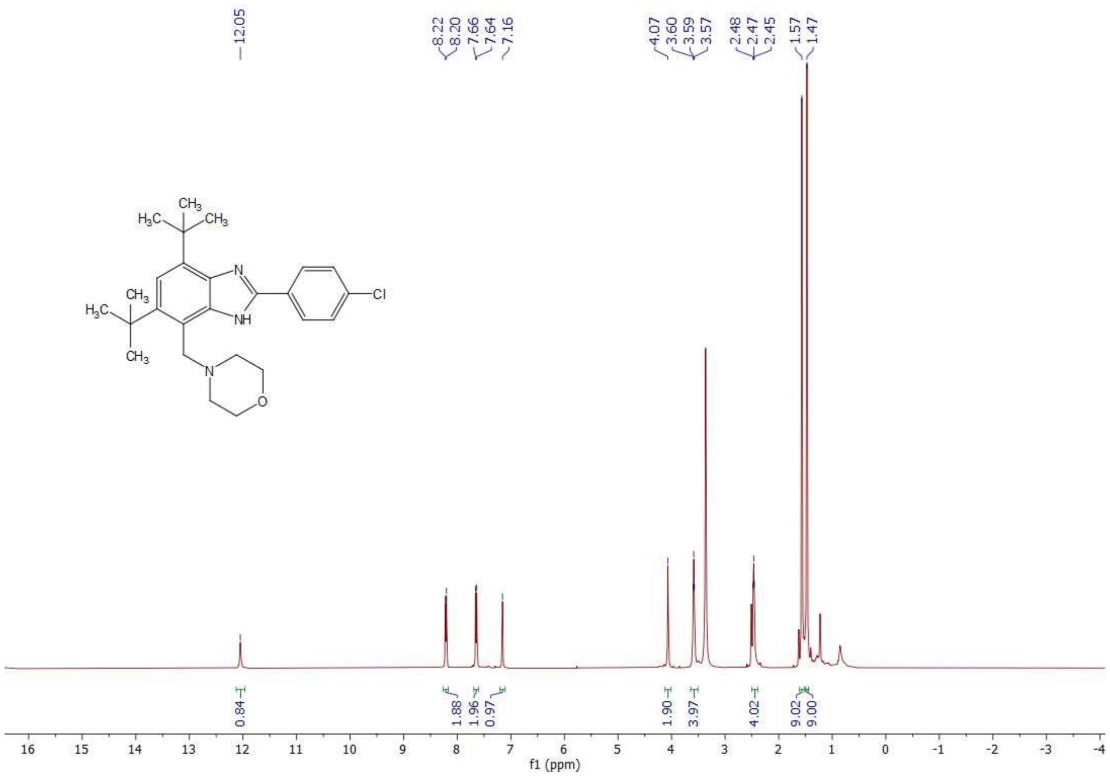

Figure S54. **^{1}H NMR spectrum of compound 4ab in DMSO-d_6 (400MHz).**

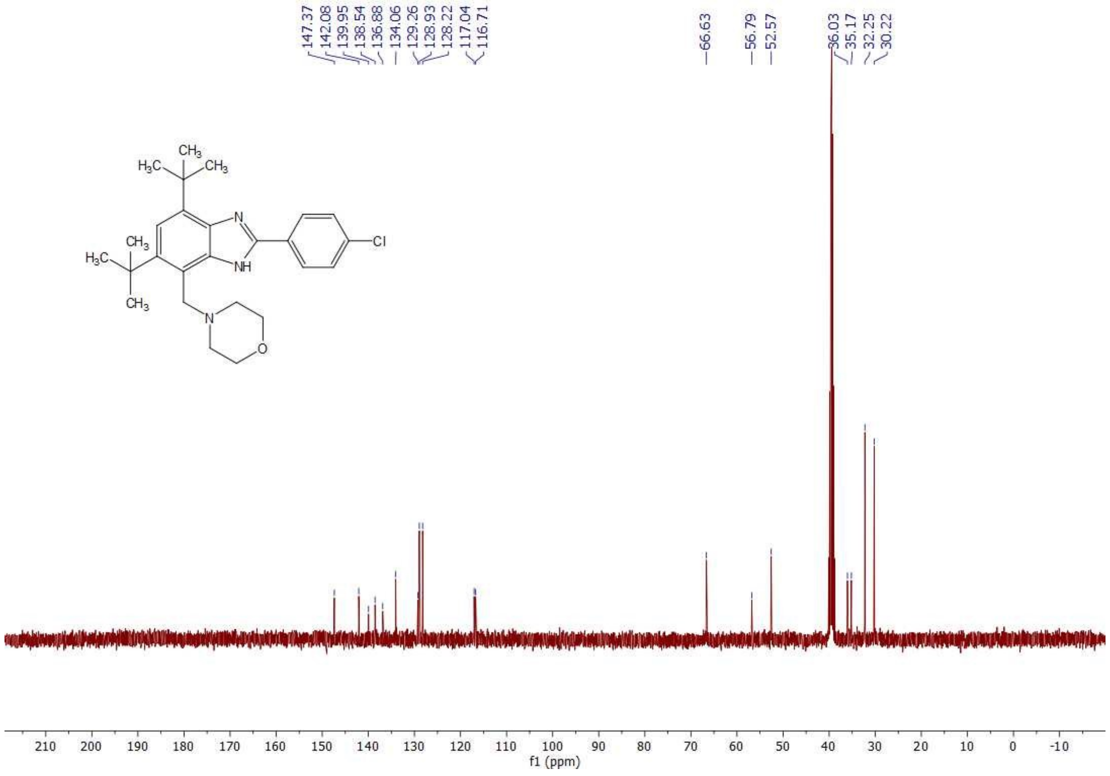

Figure S55. **^{13}C NMR spectrum of compound 4ab in DMSO-d_6 (100 MHz).**

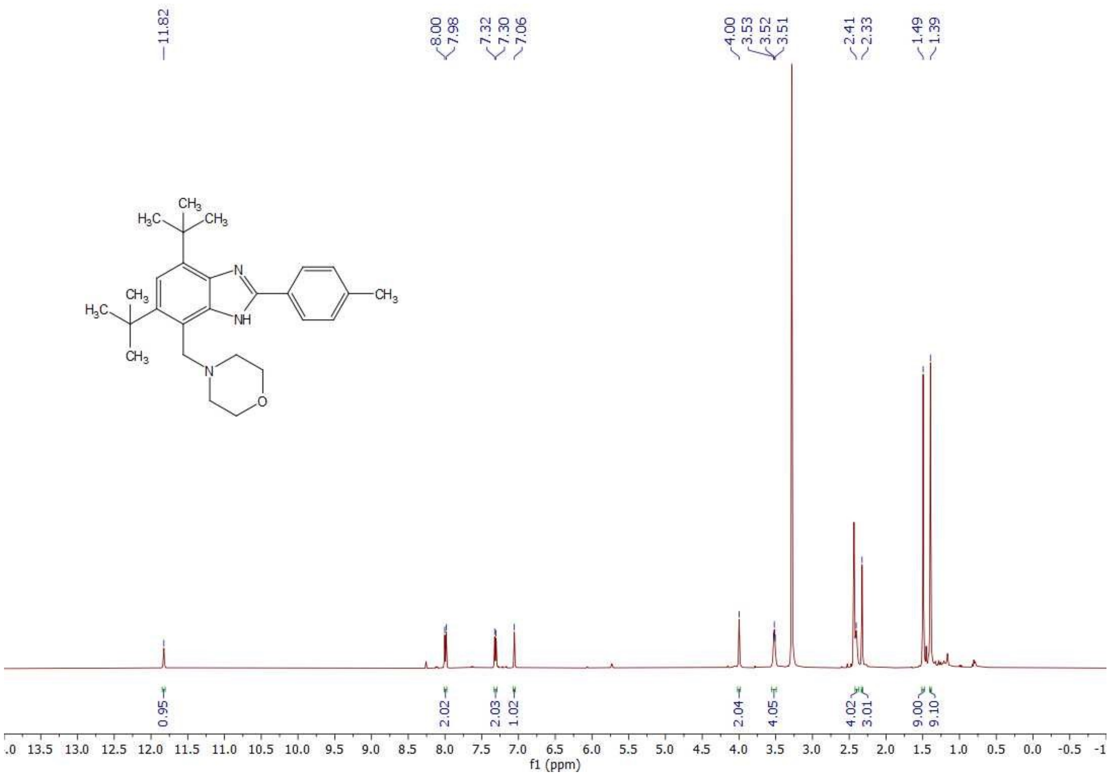

Figure S56. ^{1}**H NMR spectrum of compound 4ac in DMSO-*d*$_6$ (400MHz).**

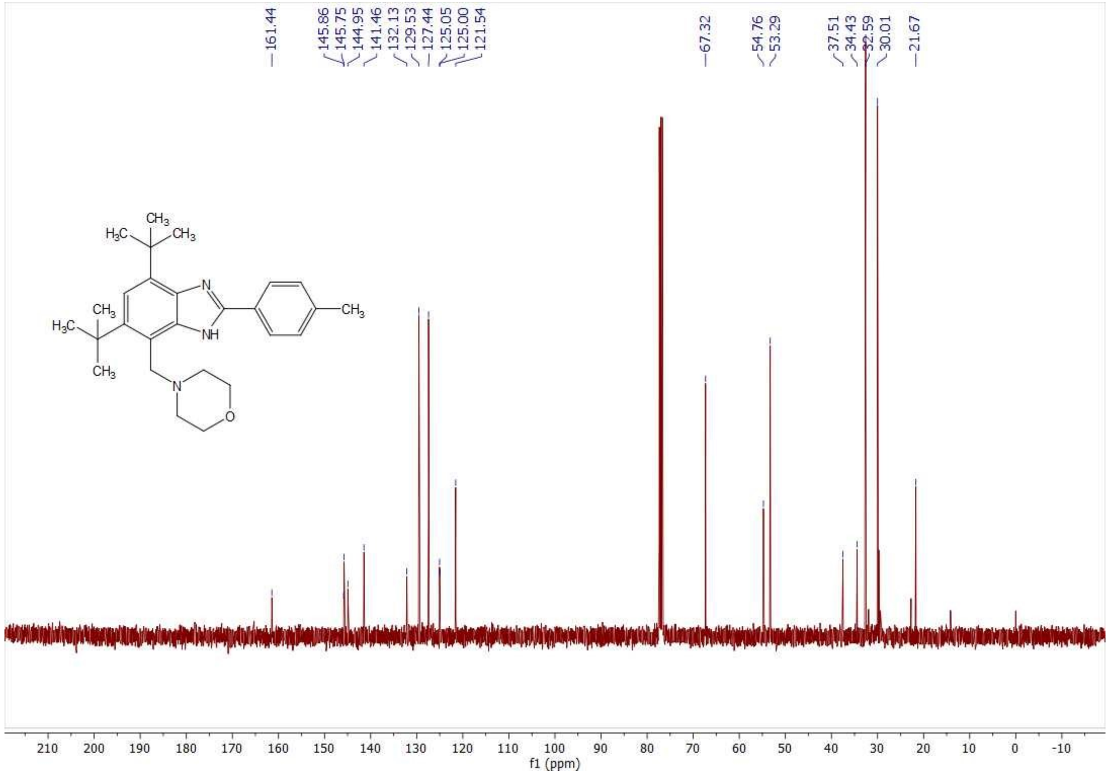

Figure S57. ^{13}**C NMR spectrum of compound 4ac in CDCl$_3$ (100 MHz).**

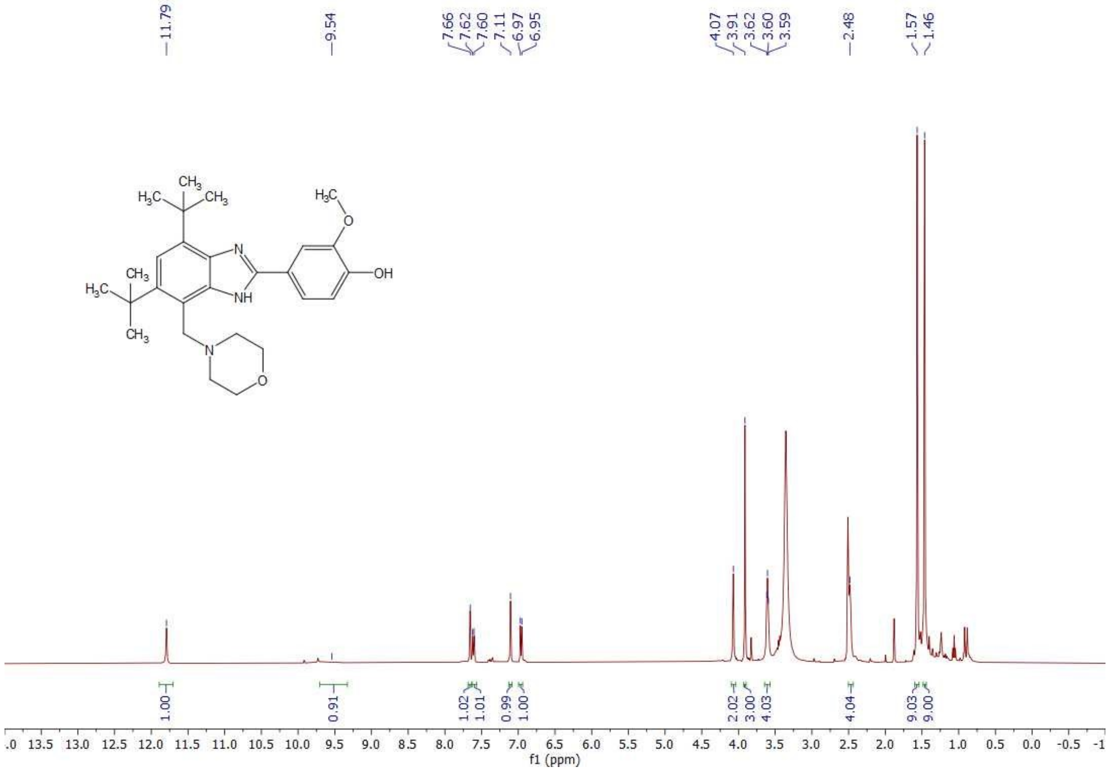

Figure S58. ^{1}H NMR spectrum of compound 4ad in DMSO-d_6 (400MHz).

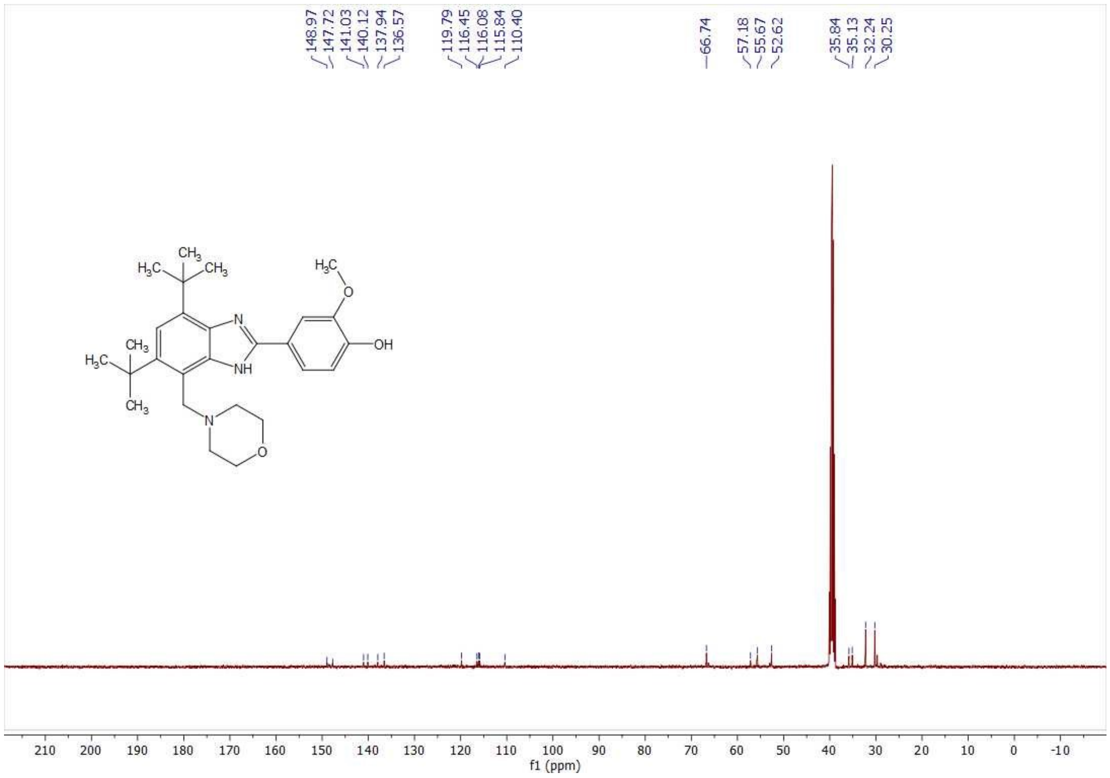

Figure S59. ^{13}C NMR spectrum of compound 4ad in DMSO-d_6 (100 MHz).

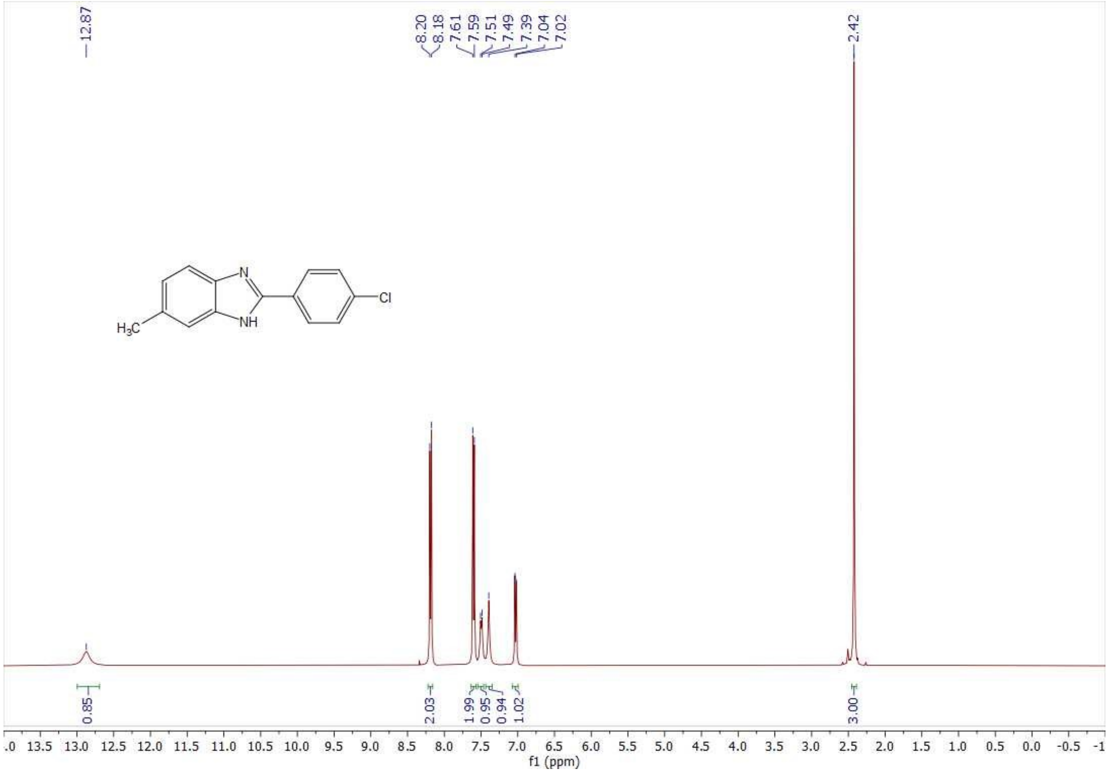

Figure S60. ^{1}**H NMR spectrum of compound 4ae in DMSO-*d*$_6$ (400MHz).**

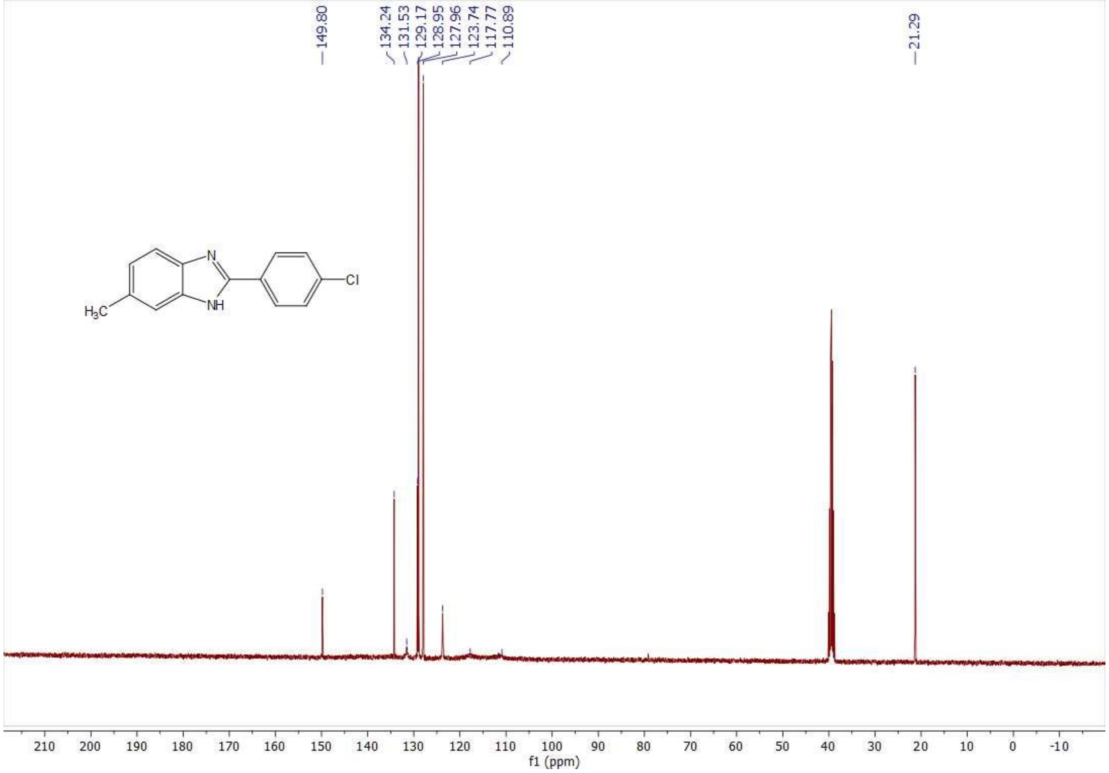

Figure S61. ^{13}**C NMR spectrum of compound 4ae in DMSO-*d*$_6$ (100 MHz).**

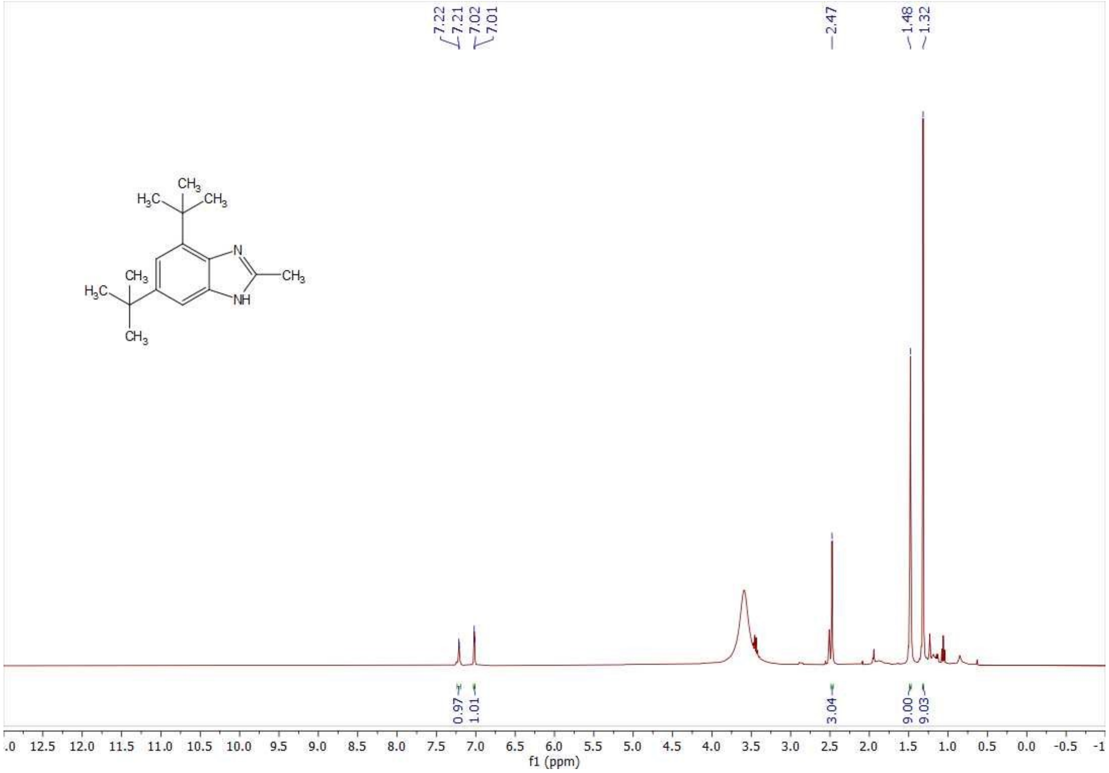

Figure S62. ^{1}**H NMR spectrum of compound 4af in DMSO-d_6 (400MHz).**

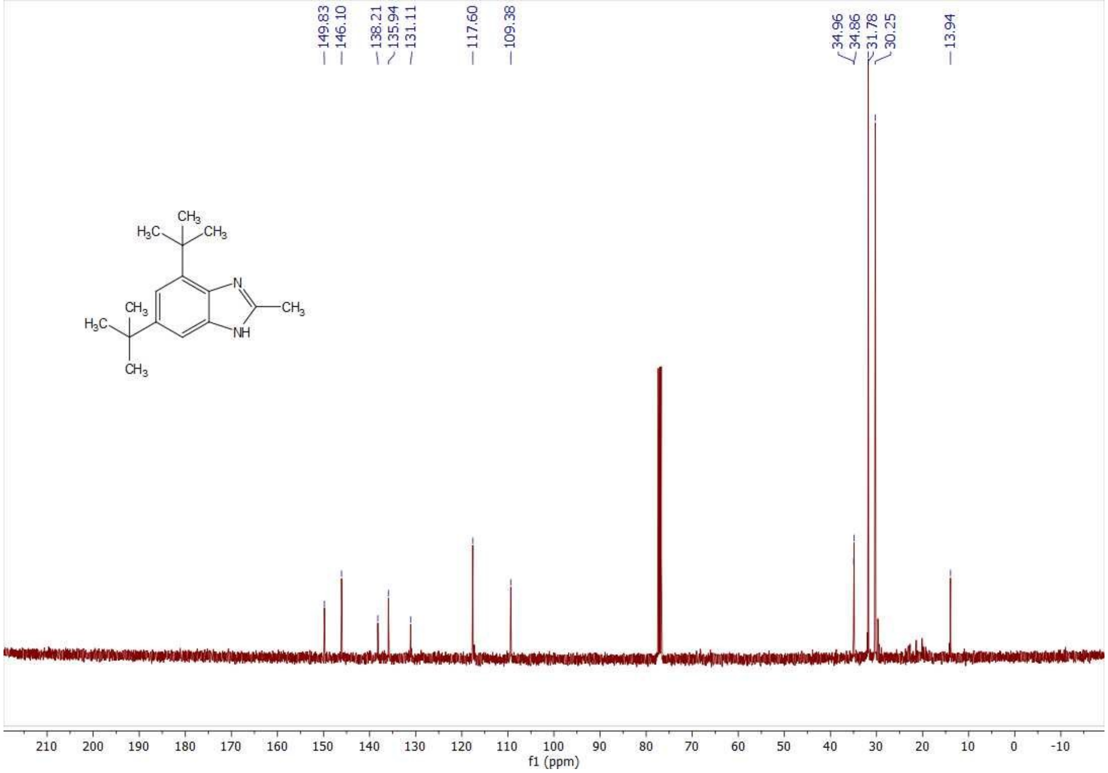

Figure S63. ^{13}**C NMR spectrum of compound 4af in DMSO-d_6 (100 MHz).**
